# Discovery of a sushi domain-containing protein 2-positive phenotype in circulating tumor cells of metastatic breast cancer patients

**DOI:** 10.1038/s41598-025-87122-4

**Published:** 2025-01-31

**Authors:** Kai Bartkowiak, Parinaz Mossahebi Mohammadi, Paula Nissen, Stefan Werner, David Agorku, Antje Andreas, Maria Geffken, Sven Peine, Karl Verpoort, Thomas M. Deutsch, Laura L. Michel, Andreas Schneeweiss, Verena Thewes, Andreas Trumpp, Olaf Hardt, Volkmar Müller, Sabine Riethdorf, Hartmut Schlüter, Klaus Pantel

**Affiliations:** 1 Department for Tumour Biology, University Medical Centre Hamburg-Eppendorf, Martinistraße 52, 20246 Hamburg, Germany; 2 Section Mass Spectrometry and Proteomics, University Medical Centre Hamburg-Eppendorf, Martinistraße 52, 20246 Hamburg, Germany; 3 Mildred Scheel Cancer Career Center HaTriCS4, University Medical Center Hamburg- Eppendorf, Martinistraße 52, 20246 Hamburg, Germany; 4 Miltenyi Biotec B.V. & Co. KG, Friedrich-Ebert-Straße 68, 51429 Bergisch Gladbach, Germany; 5 Department of Transfusion Medicine, University Medical Centre Hamburg-Eppendorf, Martinistraße 52, 20246 Hamburg, Germany; 6 Superregional group practice for hematology and oncology, Hohe Weide 17 b, 20295 Hamburg, Germany; 7 Department of Obstetrics and Gynecology, University of Heidelberg, Im Neuenheimer Feld 440, 69120 Heidelberg, Germany; 8 National Center for Tumor Diseases, Heidelberg University Hospital and German Cancer Research Center, Im Neuenheimer Feld 460, 69120 Heidelberg, Germany; 9 Division of Molecular Genetics, German Cancer Research Center (DKFZ), Im Neuenheimer Feld 280, 69120 Heidelberg, Germany; 10 Division of Stem Cells and Cancer, German Cancer Research Center (DKFZ) and DKFZ- ZMBH Alliance, Im Neuenheimer Feld 280, 69120 Heidelberg, Germany; 11 Department of Gynecology, University Medical Center Hamburg-Eppendorf, Martinistraße 52, 20246 Hamburg, Germany

**Keywords:** SUSD2, Breast cancer, Circulating tumor cells, Estrogen receptor positive, Metastasis, PDCD4, Breast cancer, Cancer

## Abstract

Cell lines derived from circulating tumor cells (CTCs) in the blood provide important biological information on cancer metastasis. CTC-ITB-01 is a CTC cell line derived from a patient with metastatic estrogen receptor-alpha (ER-alpha) positive breast cancer two months before the death of the patient. After a LC-MC/MS based proteomics analysis of CTC-ITB-01, we found extraordinary high levels of the poorly characterized protein SUSD2 (sushi domain-containing protein 2) in CTC-ITB-01. Expression of SUSD2 on subsets of CTCs was validated on clinical blood samples of patients with metastatic breast cancer. SUSD2-positive CTCs could be captured specifically by a MACS-based approach. We overexpressed SUSD2 in the poorly-metastatic cell line MCF-7. This resulted in upregulation of ER-alpha, the tumor progression protein GRP78 (78-kDa glucose-regulated protein) and downregulation of the tumor suppressor protein PDCD4 (programmed cell death protein 4). We observed downregulation of SUSD2 and PDCD4 after hypoxia and simulation of re-oxygenation in the blood in MCF-7 and MDA-MB-468, while in CTC-ITB-01 SUSD2 levels remained unchanged, and only PDCD4 was downregulated under hypoxia. In conclusion, we show, for the first time, that SUSD2 is expressed in CTCs and appears to affect key proteins in tumor progression and survival.

**Supplementary Information:**

The online version contains supplementary material available at 10.1038/s41598-025-87122-4.

## Introduction

Around 60-70% of the primary breast tumors are estrogen receptor positive^1^. Despite a good prognosis of more than 99% 5-year breast cancer-specific survival, estrogen receptor-α (ER-α) positive breast cancer is a major cause of cancer-related death in women due to its high frequency and constant risk of distant recurrence^2–4^. Estrogen receptor positive circulating tumor cells (CTCs) can be detected in the blood more than ten years after mastectomy^5^. Approximately 10% of the ER-α positive breast cancers even with T1, node-negative and low-grade show a recurrence during years 5 to 20 and endocrine resistance inevitably occurs in ER-α positive metastatic breast cancer^6,7^.

These findings rise the question by which means such cells are able to survive at secondary sites or become tolerant to therapeutic approaches. On cellular level, one possibility is the activation of the epidermal growth factor receptor (EGFR) or HER2. In such cases, the downstream activation of growth factor-driven pathways like the mammalian target of rapamycin (mTOR) pathway can activate the ER-mediated transcription in the absence of estradiol^7^. With regard to the tumor cell population as a whole, clonal evolution may provide a driving force for the evolvement of well-adapted cells in cancer^8^. This implies the selection of viable CTC clones from heterogeneous CTC pool for survival at secondary sites^9^.

From that point, CTCs are not only an attractive source to explore the mechanism of the dissemination process, but they have also prognostic value in breast cancer^10^. Practically, the analysis of CTCs may provide insights into the current status of the tumor cell genotypes and phenotypes without disturbing intervention^11^. However, only 1–10 CTCs are generally present in 10 ml blood of patients with early or non-progressive metastatic breast cancer, which limits the spectrum of analytical methods, and therefore the biological insights^12^.

We have recently established a cell line from CTC of a patient with metastatic estrogen receptor positive breast cancer, partially resistant to endocrine therapy^13,14^. The patient first received paclitaxel, followed by letrozole/denosumab and tamoxifen/denosumab. This CTC cell line – CTC-ITB-01 – remained ER-α positive in cell culture and the copy number alteration profile resembled to CTCs from the patient at the time of blood draw. CTC-ITB-01 was isolated 2 years and 3 months after diagnosis of breast cancer with metastases in the lymph nodes, bone marrow, spleen and liver. About at the time point of the isolation of CTC-ITB-01, metastases in the vagina were detected, and 2 months later the patient died. Therefore, CTC-ITB-01 offers the possibility to gain unique insights into the biology of CTCs from a very late stage of the disease.

In this work, we report the proteome analysis of CTC-ITB-01 by mass spectrometry, quantifying 7954 different proteins and using MCF-7 as a reference cell line. We found extraordinarily high levels of the protein sushi domain-containing protein 2 (SUSD2) in CTC-ITB-01. SUSD2 is a single-pass type I transmembrane protein with an extracellular domain of 757 amino acids^15^.

Even though few is known about its function, SUSD2 has been implicated in breast cancer before^16^. On a breast cancer tissue microarray, 82% of the samples were positive for SUSD2, including 41 of 47 estrogen receptor positive samples. While artificial SUSD2 overexpression in MDA-MB-231 had no effect to proliferation or anchorage-dependent growth, SUSD2 increased invasion by almost 7-fold. After injecting SUSD2 overexpressing 66CL4 cells into mice, an accelerated tumor formation and decreased survival of the mice compared with the control experiment was observed^16^.

Here we explored the proteome of CTC-ITB-01 to gain deeper insights into the biology of late-stage ER-α positive tumor cells. We found that SUSD2 overexpression leads to downregulation of programmed cell death protein 4 (PDCD4) and to upregulation of 78 kDa glucose-regulated protein (GRP78). Since SUSD2 is a type I transmembrane protein with a large extracellular domain, this allowed the establishment of tools for the specific detection and isolation of SUSD2 positive CTCs.

## Materials and methods

All uncropped X-ray film images are available in Supplementary Information.

### Cell lines and culture conditions

The cell lines were cultured in a humidified environment at 37 °C. The CTC cell line CTC-ITB-01 was derived in the Institute of Tumor Biology from the blood of a breast cancer patient^13^. CTC-ITB-01 was cultured in Roswell Park Memorial Institute (RPMI) 1640 (Pan-Biotech, Aidenbach, Germany) with 10% fetal calf serum (FCS, Capricorn, Ebsdorfergrund, Germany), 2 mM L-glutamine (Life Technologies, Darmstadt, Germany), 50 ng/ml epidermal growth factor, 10 ng/ml human basic fibroblast growth factor (both from Miltenyi Biotec, Bergisch Gladbach, Germany), 1% Insulin-Transferrin-Selenium-A Supplement, 200 U/mL Penicillin/Streptomycin (both from Gibco/Thermo Fisher Scientific, Waltham, USA), 100 ng/ml hydrocortisone and 100 ng/ml cholera toxin (both from Sigma-Aldrich, St. Louis, USA). The disseminated tumor cell (DTC) cell line from the bone marrow of a breast cancer patient BC-M1 was cultivated as described^17^. The cell line T47D (RRID: CVCL_0553) was cultured in RPMI 1640 with 10% FCS, 200 U/mL Penicillin/Streptomycin, and 2 mM L-glutamine. The cell lines MDA-MB-468 (RRID: CVCL_0419), MCF-7 (RRID: CVCL_0031), Cama-1 (RRID: CVCL_1115), and KPL-1 (RRID: CVCL_2094) were cultured in DMEM high glucose medium (Dulbecco’s Modified Eagle’s Medium) from Pan-Biotech with 10% FCS, 2 mM L-glutamine and 100 U/mL Penicillin/Streptomycin. Cells that were cultured in DMEM were maintained in presence of 10% CO_2_; in case of RPMI 1640, 5% CO_2_ was used. CTC-ITB-01 and BC-M1 were generated in the Institute of Tumor Biology^13,18^. T47D was purchased from Cell Lines Service (Eppelheim, Germany). MDA-MB-468 (09/2016) and KPL-1 were purchased from the Leibniz Institut-Deutsche Sammlung von Mikroorganismen und Zellkulturen (Braunschweig, Germany). MCF-7 and Cama-1 were purchased from the American Type Culture Collection (Manassas, USA). Authenticated cell lines (last test): MCF-7 and T47D (08/2019), Cama-1 and KPL-1 (02/2014), by Multiplexion (Heidelberg, Germany) using single nucleotide polymorphism profiling.

For the experiments, the cell lines were transiently resuscitated from liquid nitrogen stored cryo-cultures. Samples derived from the cell lines were created within six months after resuscitation of the cell lines using the passages from six to maximally twenty. After revitalization of the cells, a routine mycoplasma test was performed after five passages using the VenorGeM Classic mycoplasma detection kit (Minerva Biolabs, Berlin, Germany). After negative mycoplasma result, new cyro-cultures were generated. Mycoplasma infected cells were expunged and the incubator was disinfected.

## Determination of cell growth rates

Defined cell numbers were seeded into wells of a 6-well plate (t = 0). The number of the cells was determined after 75–150 h. For cell counting, the cells were detached from the well plate by trypsinization (Trypsin-EDTA solution 0.25% (w/v) from Gibco, Eggenstein, Germany) at 37 °C for 5 min. Trypsinization was stopped by adding 1 ml of FCS containing medium. The cell suspension was transferred into a reaction tube and centrifuged for 3 min at 1,200 × g. The cell pellet was resuspended in 500 µl of phosphate-buffered saline (PBS, Thermo Fisher Scientific) and cells were counted using a Neubauer counting chamber.

## Cell harvest and determination of protein concentration

For isolation of the protein extract, the cells were washed for three times with 37 °C warm PBS. Then, 300 µl–150 µl of lysis buffer (9.8 M urea, 30 mM tris-base and 15 mM EDTA) was added to the cells for a 75 cm^2^ or 25 cm^2^ cell culture flask, respectively. Adherent cells were collected using a cell scraper. Suspension cells were centrifuged for 3 min at 800 × g and washed three times with PBS before adding lysis buffer to the cells. Cell lysates were incubated for 2 h at room temperature and homogenized by ultrasonic treatment every 30 min. After centrifugation at 12,000 × g for 3 min, supernatant was transferred into a new reaction tube and stored at – 20 °C.

In case of the transfected MCF-7 cells (see below), cell pellets were lysed in 1 M triethylammonium hydrogen carbonate buffer (TEAB, Thermo Fisher) with 1% w/w sodium deoxycholate (SDC, Sigma Aldrich) and heated at 95 °C for 10 min. To destroy the DNA/RNA, the lysates were sonicated five times at 30% using a probe sonicator. Twenty micrograms of protein were used for tryptic digestion. Disulfide bonds were reduced through incubation in 10mM dithiothreitol (DTT, Sigma Aldrich) at 60 °C for 30 min followed by alkylation with 20 mM iodoacetamide (IAA, Sigma Aldrich) for 30 min at 37 °C protected from light. For digestion of proteins, trypsin sequencing grade (Promega, Madison, USA) was added in a 1:100 ratio (enzyme to protein) and incubated overnight at 37 °C. SDC was precipitated using 1% formic acid (FA, Fluka) with subsequent centrifugation for 10 min at 16,000 x g. The supernatant was transferred into a new tube and lyophilized using a SpeedVacTM vacuum concentrator.

The protein concentration was determined using the Pierce BCA Protein Assay Kit (Pierce, Rockford, USA) according to the instruction manual using bovine serum albumin as the standard.

For samples that were subjected to tryptic digestion, the 2D Quant Kit (GE Healthcare, Munich, Germany) was used. The calibration series of the BSA standard was prepared with BSA amounts of 0, 5, 10, 15 and 25 µg. The extinction was determined with the Ultrospec 2000 UV-VIS Spectrophotometer (Pharmacia, Uppsala, Sweden) at a wavelength of 480 nm.

## SDS-Page and western blot

For size-based separation of proteins, SDS PAGE was performed using the Novex XCell Sure-Lock mini system (Invitrogen, Groningen, Netherlands) or the Protean II xi cell (Bio-Rad, Hercules, USA) with a Laemmli buffer system^19^. Polyacrylamide gels were produced manually by casting separation and stacking gel into an empty gel cassette (Thermo Fisher Scientific) or Invitrogen Novex Tris-Glycine Mini Gradient Gels with gel percentage of 8–16% were used. Acrylamide concentrations of the separation gel were chosen depending on the molecular weight of the protein of interest and contained either 8%, 10% or 12%. Polyacrylamide gels used for separation of proteins that were subsequently analyzed by LC-MS/MS were casted as described, with casting the separation gel one day before electrophoresis to ensure complete reaction of the acrylamide monomers prior to loading the protein sample. Proteins were denaturized under reducing conditions with SDS sample buffer (0.225 M Tris·Cl, pH 6.8, 50% glycerol, 5% SDS, 0.05% bromophenol blue, 0.25 M DTT) and incubation at 95 °C for 5 min. Twenty, 30–40 µg of protein and the molecular size standard PageRuler Plus prestained protein ladder 10–250 kDa (Thermo Fisher Scientific) were loaded onto the gel. After SDS-PAGE, the proteins were transferred onto a PVDF membrane (VWR, Radnor, USA) using the mini VE vertical electrophoresis system equipped with tank blot transfer units (GE Healthcare, Uppsala, Sweden). The gel was equilibrated in transfer buffer (43.6 mM Tris base, 39 mM glycine, 10% ethanol) for 30 min. The PVDF membrane was moistened for 15 min in methanol and then equilibrated for 15 min in transfer buffer. Gel and membrane were assembled with transfer buffer soaked Western blotting filter papers into the tank blot transfer unit. After transfer, the membrane was washed for 5 min with tris-buffered saline with Tween20 (TBST) followed by an incubation step with blocking buffer for 1 h on a tube roller. Depending on the primary antibody, either 5% low fat powdered milk or 5% BSA in TBS-T was used as blocking buffer. Before each step, described as follows, the membrane was washed three times for 5 min with TBST. The membrane was incubated with primary antibody diluted in either 5% low fat powdered milk (Roth, Karlsruhe, Germany) or 5% BSA (Sigma-Aldrich) in TBST at 4 °C on a tube roller overnight. The applied primary antibodies are specified in Supplementary Table S1. On the following day, the incubation with a corresponding horseradish peroxidase (HRP)-conjugated secondary antibody (DAKO, Glostrup, Denmark), diluted in 5% low fat powdered milk in TBST, was conducted at room temperature for 1 h. Protein bands were visualized with Signal Fire Plus ECL reagent (Cell Signaling Technology, Danvers, USA) and X-ray films (Agfa Healthcare, Mortsel, Belgium) according to the supplied instruction manuals. The X-ray films were digitized using the GS-700 imaging densitometer (Bio-Rad). For the analysis, the Quantity one software (Bio-Rad) was used. To re-use the membrane, bound antibodies were removed by incubation with stripping buffer (62.4 mM Tris-base, 69.4 mM SDS, 100 mM 2-mercaptoethanol, 20 mM DTT, pH 9.5) for 1 h, followed by careful washing with TBST. After blocking with the corresponding buffer, another primary antibody incubation was performed.

## Colloidal coomassie staining of polyacrylamide gels

SDS-polyacrylamide gels were stained by colloidal coomassie staining according to Neuhoff^20^ to validate protein concentrations or to visualize protein expression patterns.

### Stable isotope labeling by amino acids in cell culture (SILAC)

For the comparison of the protein profiles of CTC-ITB-01 and MCF-7, the labelling approach SILAC was used applying the SILAC Protein Quantitation Kit (Lys C) RPMI 1640 (Thermo Fisher Scientific) according to the manufacturer’s instructions. MCF-7 was cultured in DMEM for SILAC with 10% dialyzed fetal bovine serum. The proteins of MCF-7 were labelled with 0.46 mM^13^C_6_ lysine × 2 HCl and 0.47 mM^13^C_6_ arginine × HCl (heavy). CTC-ITB-01 was cultured in RPMI 1640 for SILAC with 10% dialyzed fetal bovine serum and 0.46 mM^12^C_6_ lysine × 2 HCl and 0.47 mM^12^C_6_ arginine × HCl (light). To minimize potential false labeling of proline, the cell culture media were supplemented with^12^C_5_ proline (Thermo Fisher Scientific) to a final proline concentration of 0.48 mM in the media. The other supplements were the same as described above. Cells were passaged three times before protein isolation to ensure complete incorporation of the heavy amino acids.

For the determination of complete labelling of the MCF-7 proteins with^13^C_6_ lysine and^13^C_6_ arginine, the cells were cultured for five passages in heavy SILAC medium. Twenty micrograms of protein extracts of each passage were applied to SDS-PAGE and the gel was stained with colloidal coomassie. Gel slices were excised at approximately 40 kDa and 100 kDa. In-gel reduction, alkylation with iodoacetamide (IAA), tryptic digest, and extraction of the peptides were performed as described^21^. The mass spectrometry was performed as described below. The results showed that cultivation until passage 3 led to complete incorporation of heavy labeled amino acids. Cultivation to passage 4 or 5 showed no improvement in incorporation of heavy arginine and lysine and in some cases even re-appearance of a weak light peptide ion signal was observed. Therefore, cultivation of MCF-7 in heavy SILAC medium for three passages was performed for the preparative assay.

For the identification of SUSD2 peptides at approximately 55 kDa, forty micrograms of CTC-ITB-01 total cell extract was loaded on a polyacrylamide gel followed by a Coomassie blue staining. The gel region of about 55 kDa was excised, and processed as described^21^ using trypsin as protease. The obtained tryptic peptides were subjected to LC-MS/MS as described below.

The cell harvest of the SILAC labelled cells and the processing of the protein extracts was performed as described^22^. For one biological sample to be compared, 400 µg of protein extract each of MCF-7 (heavy) and CTC-ITB-01 (light) were combined in 1:1 ratio and subjected to tryptic digestion. Five biological samples of each cell line were analyzed.

## Peptide desalting and OFFGEL fractionation

To remove unwanted components from the tryptic peptides prior to isoelectric focusing, a purification step was performed. The sample was adjusted to 5% methanol and 0.2% formic acid to a volume of 1 ml. The Sep-Pak C18 1 cc Vac Cartridge (Waters, Manchester, UK) column was conditioned with 6 ml methanol and equilibrated with 3 ml 5% methanol/0.2% formic acid in water. After application of the sample to the column, unwanted components were removed by adding 3 ml of 5% methanol/0.2% formic acid in water to the column. Peptides were eluted with 1 ml of 50% acetonitrile, followed by a further elution step with 0.5 ml of 75% acetonitrile. The desalted sample was evaporated to complete dryness using a vacuum-centrifuge. The OFFGEL fractionation was performed as described^22^.

## LC-MS/MS and data analysis

The LC-MS/MS and data analyses were essentially performed as described^8^. The tryptic peptides of the transfected MCF-7 cells (MCF-7 control, MCF-7 OE2) were applied without prior fractionation. As mobile phase A: 0.1% (v/v) formic acid in water, and as mobile phase B: 0.1% (v/v) formic acid in acetonitrile was used. Samples were dissolved in 20 µl of mobile phase A to achieve a concentration of 1 µg/µL. The samples were injected on a nano UHPLC (Dionex Ultimate 3000 UHPLC system, Thermo Fisher Scientific) coupled to a tribrid quadrupole-ion-trap-orbitrap MS (Orbitrap Fusion, Thermo Fisher, Bremen, Germany). A half microliter of sample was loaded on a peptide trap column (100 μm x 20 mm, 100 Å pore size, 5 μm particle size, C18, Nano Viper, Thermo Fisher) for online desalting and purification. Peptides were eluted onto a 25 cm C18 reversed-phase column (75 μm x 250 mm, 130 Å pore size, 1.7 μm particle size, peptide BEH C18, nanoEase, Waters) and were separated using an 80 min method with linearly increasing ACN concentration from 2 to 30% ACN over 60 min. Ionization of the eluted peptides was performed using a nano electrospray ionization source (nano ESI) with a spray voltage of 1,800 V. For each MS1 scan ions were accumulated for a maximum time of 120 ms or until a charge density of 2 × 10^5^ ions (AGC target) was reached. Fourier-transformation based mass analysis of the data from the orbitrap mass analyzer was performed over a mass range of m/z 400 to 1,300 with a resolution of 120,000 at m/z = 200. Peptides carrying charges between 2 + and 5 + and above an intensity threshold of 1,000 were isolated within a m/z 1.6 isolation window in Top Speed mode for 3 s from each precursor scan. The isolated peptides were fragmented with a normalized collision energy of 30% using higher energy collisional dissociation (HCD). MS2 scanning was achieved using an ion trap mass analyzer at a rapid scan rate, covering a mass range starting at m/z 120 and accumulated for 60 ms or to an AGC target of 1 × 10^5^.

The raw data obtained from the MCF-7 transfected cells were searched with the Sequest HT algorithm integrated in Proteome Discoverer software (v 2.4–1.13, Thermo Fisher Scientific) against a reviewed human Swissprot database, obtained in April 2020, containing 20,365 entries. Carbamidomethylation was set as fixed modification and methionine oxidation as variable modification. Only peptides between 6 and 144 amino acids and with a maximum of 2 missing tryptic cleavages were considered. A false discovery rate (FDR) of < 0.01 was set for proteins and peptide identification. The data analysis of the relative protein abundances was performed using the Perseus software (Max Plank Institute for Biochemistry, Munich, Germany; v 1.6.15.0). The values were log2 transformed and column-median normalized at the protein level. The data was filtered for proteins present in at least three samples. Principal component analysis was performed to estimate distinguishability of compared groups and was achieved by using proteins only present in all samples.

The raw data files from the other experiments were processed by the MaxQuant software (version 1.5.8.3)^23^ which is a quantitative proteomics software for analysis of high-resolution MS data. Proteins were identified with the peptide search engine Andromeda against a reviewed human data base obtained from UniProtKB (www.uniprot.org, downloaded November 2017; 20,239 entries) and a contaminant database (provided by MaxQuant, 245 entries). Searches were performed with a mass tolerance of 20 ppm on MS level and a mass tolerance of 0.5 Da on MS/MS level. The number of MS_1_ labels was two, with^13^C_6_ arginine and^13^C_6_ lysine as heavy labels. A maximum of two missed cleavages was allowed for protein identification. Cysteine carbamidomethylation was set as fixed modification and the oxidation on methionine, acetylation on protein N-terminus and deamidation on asparagine and glutamine were set as variable modifications. Not more than 5 modifications per peptide were allowed. The search was performed with a FDR of < 0.01 on both peptide and protein level. For SILAC quantification, only unique peptides were considered (unmodified and peptides acetylated on protein N-terminus). Student’s *t*-test was used for statistical analysis of all differential quantitative proteomics data. A *p*-value below 0.05 was considered as significant. The expression ratio values for MCF-7 were converted into reciprocal values to facilitate the evaluation and the negative value was used to distinguish these values from those from CTC-ITB-01.

For the gene set enrichment analysis (GSEA), obtained relative protein abundances or normalized heavy-to-light ratios for SILAC data were loaded into Perseus software (Max Plank Institute for Biochemistry, v 1.6.15.0), log2 transformed and column median normalized at the protein level, for label-free data. For SILAC data Student’s one-sample test was performed, and Student’s two-sample test was applied for the comparison of the SUSD2 overexpressed group and the control. For the GSEA of the SILAC data from CTC-ITB-01 vs. MCF-7, protein values were filtered for at least three valid values in the individual biological replicates. Proteins with an *p*-value of < 0.05 or a *q*-value of < 0.05 (FDR-corrected), as well as with a fold change of > 2 were considered significant. The GSEA data from the comparison of SUSD2 OE vs. control were performed in a similar way, with the difference that a fold change of > 1.5 was used for the analysis. The sample F3 Ctrl (see Fig. 4A of the main text) was excluded from the analysis. GSEA with gene ontology (GO) terms in molecular functions (MF), biological process (BP) and cellular component (CC) was performed and visualized with the *p*-value and fold change significantly abundant proteins in R studio^24–26^.

### Immunocytochemical staining

#### Isolation of mononuclear cells from peripheral blood

Peripheral blood mononuclear cells (PBMC) from healthy women were isolated by density gradient centrifugation using Ficoll-Paque (Cytiva, Marlborough, USA). Whole blood (7.5 ml) was diluted with PBS to a volume of 15 ml. Diluted blood was gently layered on top of 20 ml of Ficoll Paque density gradient medium. After centrifugation for 30 min at 400 × g without using the centrifuge breaks, the upper layer containing plasma and the mononuclear cell layer at the interface were transferred to a new centrifuge tube. Diluted with PBS to a volume of 40 ml, mononuclear cells were pelletized by centrifugation at 400 × g for 10 min. For the immunocytochemical detection, cell line cells were spiked into the obtained PBMC fraction. The sample preparation of the PBMC for Western Blot analysis was performed as described^27^.

### Preparation of cytospins

For immunocytochemical analysis of cell line cells, cytospins were prepared by generating a cell suspension in PBS. An aliquot of the cell suspension was transferred into a big funnel chamber fixed on a glass slide. Cells were centrifuged for 3 min at 244 × *g* onto the glass slide. In most cases, an aliquot of the cell line suspension was first spiked into healthy donor blood to mimic a patient sample containing CTCs. Density gradient centrifugation was performed as described to isolate mononuclear cells and tumor cells. The obtained cell suspension was centrifuged onto a glass slide as described before. In case of the detection of PDCD4 in CTC of breast cancer patients, the PBMC fraction from the whole blood of breast cancer patients was obtained as described. Aliquots of the PBMC fractions were spun on cytospins and analyzed by immunocytochemical staining. Cytospins were dried overnight and stored at – 80 °C.

### Immunocytochemical staining

For immunocytochemical staining of proteins, prepared cytospins were used. After each step, except the blocking step, cytospins were washed with PBS 3 times for 2 min each. All antibodies were diluted in blocking solution consisting of 10% human AB serum (Bio-Rad) in Dulbecco´s Phosphate Buffered Saline (DPBS, Life Technologies, Carlsbad, USA). First, cells were fixed by incubation with 2% paraformaldehyde in DPBS for 10 min. Cells were permeabilized with 0.1% Triton X 100 (Sigma-Aldrich, St. Louis, USA) in DPBS for 10 min. Blocking was carried out with blocking solution for 30 min. Then, the cytospin was incubated with the primary antibody against PDCD4 for 1 h. This was followed by application of the Alexa 546 donkey anti-rabbit secondary antibody (Thermo Fisher Scientific) at a dilution of 1:200 in blocking buffer for 45 min. In case of the detection of the SUSD2 MicroBeads (see below) after magnetic-activated cell sorting (MACS), a goat anti mouse IgG Alexa Fluor 546 (Thermo Fisher Scientific) secondary antibody was used at a dilution of 1:200 in blocking buffer. The anti-PDCD4 antibody (Cell Signaling Technology, clone D29C6) was applied 1:100 in blocking solution. The anti-SUSD2-Vio Bright FITC antibody (Miltenyi Biotec, Bergisch Gladbach, Germany) was applied 1:10 in blocking solution.

For the detection and exclusion of CD45-positive leukocytes, the APC-conjugated anti-CD45 antibody (Miltenyi Biotec, clone REA737) was used in a dilution of 1:100. Nuclei were stained with 4′,6-diamidino-2-phenylindole (DAPI; Roth), in which DAPI was applied at a dilution of 1:1000. When a prior staining against PDCD4 or the SUSD2 microbeads was performed, keratin was detected using the Pan Cytokeratin Monoclonal Antibody, Alexa Fluor 488 (Thermo Fisher Scientific, clones AE1/AE3) in a dilution of 1:300. When the anti- SUSD2-Vio Bright FITC was first applied, keratin was detected using the Pan Cytokeratin Monoclonal Antibody, eFluor 570 (Thermo Fisher Scientific, clones AE1/AE3).

The slides were covered with one drop of mounting medium Prolong Gold Antifade (Thermo Fisher Scientific), and the cover slip was fixed with fixogum (Marabu, Ludwigsburg, Germany). The microscope slides were analyzed manually using the microscope Axioplan2 (Carl Zeiss, Oberkochen, Germany) with the software AxioVision 4.8 (Carl Zeiss, Oberkochen, Germany).

### Cell transfection

MCF-7 cells were stably transfected with the plasmid pEZ-M68 containing the mRNA sequence of the human SUSD2 gene (product# EX-T2052-M68, Accession No.: NM_019601, GeneCopoeia, Rockville, USA; MCF-7 OE1, MCF-7 OE2). As a control, the plasmid without an insert was used (product# EX-NEG-M68, GeneCopoeia; MCF-7 control). For the transfection of the cells, the Helix-IN transfection reagent (OZ Biosciences, Marseille, France) was used according to the manufacturer’s instructions. Two days after transfection, the DMEM cell culture medium was supplemented with 1 µg/ml puromycin (Sigma-Aldrich) and after selection of individual clones, the cells were cultivated in presence of 3 µg/ml puromycin. The treatment of the cells with 17-β-estradiol (E2) was performed as described^13^. 17-β-estradiol was purchased from Sigma-Aldrich, and the final concentration in the cell culture medium was 100 nmol/ml.

### Patients

Written informed consent was obtained from all patients. The human investigations were carried out in accordance with the Helsinki rules after approval was obtained by the ethics committee of the Medical Association Hamburg (reference number PV5392). Blood samples from women with metastatic breast cancer were obtained from University Hospital Heidelberg, Heidelberg, Germany, for the determination of SUSD2 expression on CTCs by the CellSearch system. Blood samples of women with metastatic breast cancer from the Practice for Hematology and Oncology, Hamburg, Germany were used for the isolation of SUSD2 positive CTCs with the MACS system, or for the detection of PDCD4 in CTC. Blood samples from healthy individuals were collected from the Institute of Transfusion Medicine, UKE.

### SUSD2 detection in the blood of patients by cellSearch

Seven and a half milliliters of blood were drawn from patients with metastatic breast cancer into CellSave Preservative tubes (Menarini Silicon Biosystems, Bologna, Italy). The isolation, detection and enumeration of CTCs was performed using the CellSearch system applying the CellSearch Epithelial Kit (Menarini Silicon Biosystems).

The processing of the samples was performed as described^28^. In brief, the samples were automatically processed by the CellTracks Autoprep system. EpCAM-expressing cells were immunomagnetically captured and stained for immunofluorescence analysis with antibodies specific to keratins conjugated to phycoerythrin (PE) and an antibody specific to CD45 conjugated to allophycocyanin (APC), while nuclei were stained with DAPI. Additionally, anti-SUSD2-Vio Bright FITC antibody (Miltenyi Biotec) was applied at a dilution of 1:5 to investigate the SUSD2 expression on the enriched cells. The cells were automatically transferred to a cartridge, which was placed to the CellTracks Analyzer II for automatic fluorescence analysis. Captured images of cells that fulfill previously determined criteria were automatically presented in a gallery. Final classification and evaluation of the cells was performed by Sabine Riethdorf.

For the establishment of the SUSD2 detection in CTCs, the anti-SUSD2-Vio Bright FITC antibody was used. Five hundred cells from CTC-ITB-01, MDA-MB-468 (positive controls) and T47D (negative control) were spiked into 7.5 ml EDTA-blood of a healthy female donor. The cells were detached from the cell culture flask with 3 ml DPBS containing 10 mM EDTA for five minutes at 37 °C. The EDTA-blood containing spiked tumor cells was transferred to CellSave preservative tube (Menarini Silicon Biosystems) and processed as described above.

### Magnetic cell separation (MACS)

For the isolation of SUSD2 positive cells, SUSD2 MicroBeads were used, which are 50-nm superparamagnetic particles that are conjugated to the antibody against SUSD2 (Miltenyi Biotec). For the estimation of the recovery rate of the MACS approach, PBMC from healthy women were isolated as described, and 50 cells of CTC-ITB-01 or T47D were added to the PBMC fraction. Cell pellets were resuspended in 70 µl MACS buffer (autoMACS Rinsing Solution supplemented with MACS BSA Stock Solution, both Miltenyi Biotec) and 10 µl of FcR Blocking Reagent (Miltenyi Biotec). The sample was then incubated with 20 µl of SUSD2-MicroBeads for 30 min at 4 °C in the dark. In the meantime, a LS Column (Miltenyi Biotec), containing ferromagnetic spheres, was placed in the VarioMACS Seperator (Miltenyi Biotec), which is a permanent magnet that causes a high-gradient magnetic field within the column. The column was conditioned with 3 ml of MACS buffer. The sample was diluted with 1 ml of MACS buffer and then applied onto the column. The following washing step was performed three times with 3 ml of MACS buffer. For the elution of labeled cells, the column was removed from the separator and cells were eluted with 3 ml of MACS buffer and another 2 ml of MACS buffer using the plunger to flush out all cells. One cytospin was generated from the combined flow-through and first washing fraction and another cytospin was obtained from the elution fraction. Patient samples were subjected to the same steps after PBMCs and tumor cells of patient blood samples were isolated with Ficoll Paque. Volumes given above are for up to 10^7^ total cells. When working with more than 10^7^ cells, volumes were adjusted accordingly. The detection of SUSD2 positive cells was performed as described above using the goat anti mouse IgG Alexa Fluor 546 secondary antibody together with keratin and CD45 detection and DAPI staining.

### In silico validation

For in silico validation, pre-processed gene expression data along with clinical information from the TCGA Breast Invasive Carcinoma dataset^29^ consisting of 1084 breast cancer patients were retrieved from the cBio cancer genomics portal^30^. To compare PDCD4 gene expression in different groups, samples were grouped according to their molecular subtype, tumor, or metastatic stage. Statistical tests were performed by Kruskal-Wallis test for comparison PDCD4 gene expression in molecular subtypes and tumor stages or by two-tailed t-test in metastatic stages. Differences in overall survival between these groups were determined by Kaplan-Meier analysis using the log-rank test.

## Results

### Characterization of CTC-ITB-01 for breast cancer relevant marker proteins

**Fig. 1:**
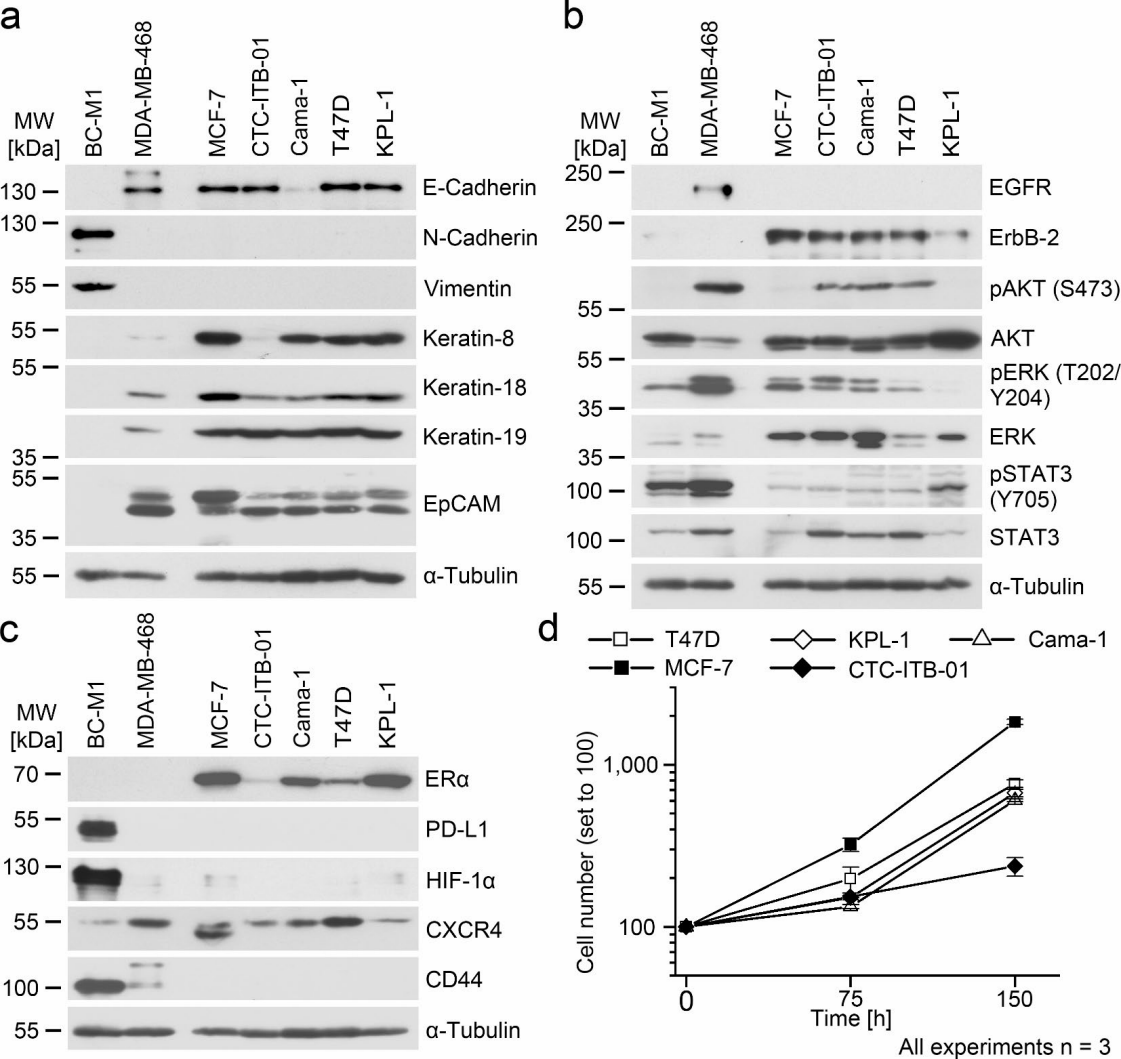
Characterization of the CTC cell line CTC-ITB-01, analyses by Western Blot using three biological replicates.**A**: Determination of the epithelial phenotype.**B**: Analysis of receptor tyrosine kinase signaling.**C**: Other breast cancer relevant proteins.**D**: Growth curves of the denoted cell lines. At the time point 0 h, 40,000 cells of each cell lines were counted and were set to 100. Vertical error bars show the standard deviation of three independent measurements. Uncropped X-ray film images are shown in supplementary information.

The CTC cell line CTC-ITB-01 reflects key features of in situ CTCs from the breast cancer patient of which CTC-ITB-01 was isolated^13^. To investigate if CTC-ITB-01 maintained its phenotype in cell culture, we analyzed here a set of phenotypic marker proteins. We confirmed that CTC-ITB-01 maintained its epithelial (Fig. 1A), ErbB-2 weakly positive and EGFR negative phenotype (Fig. 1B), as well as the low expression of estrogen receptor-α (ER-α), and the absence of CD44 (Fig. 1C) in cell culture. CTC-ITB-01 is positive for activated AKT (S473) and ERK (T202/Y204), whereas only weak phosphorylation of STAT3 (Y705) was detected (Fig. 1B). Both the cell surface protein PD-L1, which regulates T cell activation and the master regulator of the metabolic adaptation to hypoxia – hypoxia-inducible factor 1 – were not detected in CTC-ITB-01. CTC-ITB-01 is positive for the chemokine receptor CXCR4, which supports the settlement in the bone marrow (Fig. 1C). CTC-ITB-01 appears to be a representative of the luminal breast cancer subtype, similar to MCF-7, Cama-1, T47D, or KPL-1. Yet, CTC-ITB-01 growth slower than the cell lines derived from solid tumors (Fig. 1D).

### In-depth analysis of the CTC-ITB-01 protein profile by LC-MS/MS

Since few phenotypic attributes of CTCs are known, we analyzed the global protein profile of CTC-ITB-01 by LC-MS/MS. As a reference cell line for CTC-ITB-01 we selected a breast cancer cell line of the same subtype (ER-α positive). We assumed that the proteome a cell line of the same subtype is similar to CTC-ITB-01, so that we can detect a large amount of proteins in both cell lines enabling the quantitative representation of as many proteins as possible. From the analyzed ER-α positive cell lines we selected MCF-7, since this cell line is easy to cultivate, which increases the chance of a successful incorporation of the “heavy” amino acids using SILAC. CTC-ITB-01 was cultured in presence of “light” amino acids. In total, we analyzed 7954 different proteins by LC-MS/MS in five biological replicates. To increase the reliability of the findings, only proteins that were detected by at least four unique peptides were considered further (5833 proteins). From these proteins, 3945 proteins showed a fold change of 2 or higher between CTC-ITB-01 and MCF-7. A distribution pattern of the differentially expressed proteins in relation to their statistical significance is shown in Supplementary Figure S1. To get an overview about the architecture of the protein expression of CTC-ITB-01 in comparison with MCF-7, we performed a gene set enrichment analysis (GSEA). The GSEA was divided into the subcategories molecular functions, biological process and cellular component (Supplementary Figure S2). We noted a significant deviation of the structural constituent of the ribosome in favor of MCF-7 compared with CTC-ITB-01, suggesting certain alterations in the protein synthesis machinery of CTC-ITB-01.

To test the quality of the obtained protein quantification, we selected three proteins for which we already established the western blot (Table 1, Supplementary Table S2, Supplementary Figure S3). Despite applying two entirely different protein quantification methods (Western Blot vs. SILAC), the direction of the differential expression was confirmed for all three proteins.

**Table 1:** Protein identification details by SILAC and LC-MS/MS. protein samples of CTC-ITB-01 and MCF-7 were compared. A positive value of the arithmetic mean signifies an increased protein expression in CTC-ITB-01, and a negative value signifies an increased protein expression in MCF-7. *p*-value: Student’s two-sided *t*-test, with *p* < 0.05 was considered significant. See supplementary table S2 for details.

Swiss-Prot acc no.	Recommended protein name (UniProtKB/Swiss-Prot) (short name)	Total number of peptides analyzed	Number of unique peptides analyzed	Detected in biological replicates	Arithmetic mean ± standard deviation	Sequence coverage [%]	*p*-value
P18084	Integrin beta-5 (ITGB5)	35	13	5	7.01 ± 2.57	27.7	1.53 × 10^− 9^
P06396	Gelsolin (GSN)	56	16	5	2.63 ± 0.53	47.6	7.83 × 10^− 9^
P09382	Galectin-1 (LGALS1)	37	9	5	-7.87 ± 1.78	92.6	3.56 × 10^− 7^
Q9UGT4	Sushi domain-containing protein 2 (SUSD2)	112	27	5	CTC-ITB-01 only	69.2	-
P22413	Ectonucleotide pyrophosphatase/phosphodiesterase family member 1 (ENPP1)	23	8	5	2.94 ± 0.54	17.7	1.27 × 10^− 7^
Q9UIW2	Plexin-A1 (PLXNA1)	20	12	5	2.32 ± 0.56	9.8	4.30 × 10^− 4^

### Identification of transmembrane proteins overexpressed in CTC-ITB-01

**Fig. 2:**
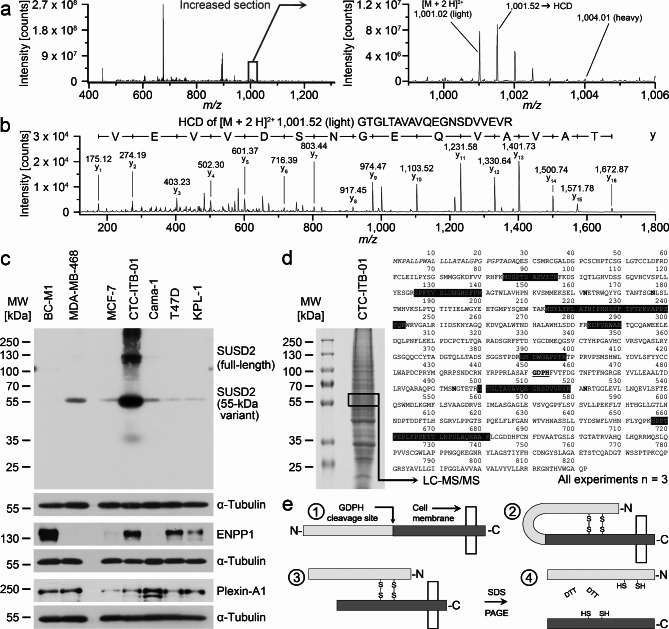
Detection and validation of differentially expressed proteins from the SILAC approach. A: Detection of SUSD2 by SILAC LC-MS/MS. Left image: MS survey scan containing the peptides around *m/z* 1001. Right: enlarged section of the SUSD2 peptides. CTC-ITB-01 was cultured in presence of light amino acids (light);^13^C_6_-labelled peptides from MCF-7 were not detected in this analysis. B: Positive ion mode HCD (higher-energy collisional dissociation) fragment spectrum of *m/z* 1001.52 [M + 2 H]^2+^ from the SILAC approach. Relevant fragments of the y-ion series are assigned with their masses. C: Validation of the denoted proteins by Western Blot. Alpha-tubulin served as loading controls. D: SDS-PAGE of protein extract from CTC-ITB-01 and marking of the excised gel area analyzed by LC-MS/MS (left). Right: Assignment of the identified peptides by LC-MS/MS in the amino acid sequence of SUSD2 (black background). Underlined and bold: GDPH cleavage sentence. Bold: predicted N-glycosylation sites. Italic: signal peptide. E: schematic representation of the posttranslational processing of SUSD2. (1) Full-length 130-kDa SUSD2 is anchored with the C-terminus in the cell membrane and contains a GDPH cleavage site at amino acid 452. (2) Disulfide-bridges are formed so that the N-terminal end is bent back towards the C-terminus of SUSD2. (3) Cleavage at the GDPD sequence results in a separate polypeptide chain that is linked to the N-terminal fragment by disulfide bridges. (4) Application of a reducing agent like DTT cleaves the disulfide bridges. Both the N-terminal (light grey) and the C-terminal fragment (dark gray) migrate at about 55 kDa by SDS-PAGE. The processing of SUSD2 was resolved by Patrick and Egland^15^. Uncropped X-ray film images are shown in supplementary file S1.

Due to the consistent results of the test proteins we next focused on transmembrane proteins with large extracellular domains. SUSD2 was strongly overexpressed in CTC-ITB-01 compared to MCF-7 in SILAC-based LC-MS/MS analysis (Fig. 2A and B; Table 1, Supplementary Table S2). CTC-ITB-01 showed by far the highest SUSD2 levels in the ER-α positive analyzed breast cancer cell lines (Fig. 2C). Further, we confirmed the elevated expression levels of two other transmembrane proteins in CTC-ITB-01 compared with MCF-7 (Fig. 2C; Table 1, Supplementary Table S2). These two proteins were ectonucleotide pyrophosphatase/phosphodiesterase family member 1 (ENPP1) and plexin-A1 (PLXNA1). Focusing on SUSD2, we noticed the dominant signal was at approx. 55 kDa by western blot instead of expected mass of approx. 130 kDa for the full-length protein (Fig. 2C). To clarify this, we separated protein extract of CTC-ITB-01 by SDS-PAGE, excised the gel region of around 55 kDa, and analyzed the extracted proteins by mass spectrometry (Fig. 2D). Within this gel piece we identified SUSD2 peptides distributed all over the protein sequence of SUSD2, suggesting the presence of the entire polypeptide chain at approx. 55 kDa. The nature of this observation is a special kind of posttranslational processing of SUSD2, which was resolved by Patrick and Egland before^15^ and is summarized in Fig. 2E.

#### Specifically overexpressed proteins in the mTOR pathway

**Fig. 3:**
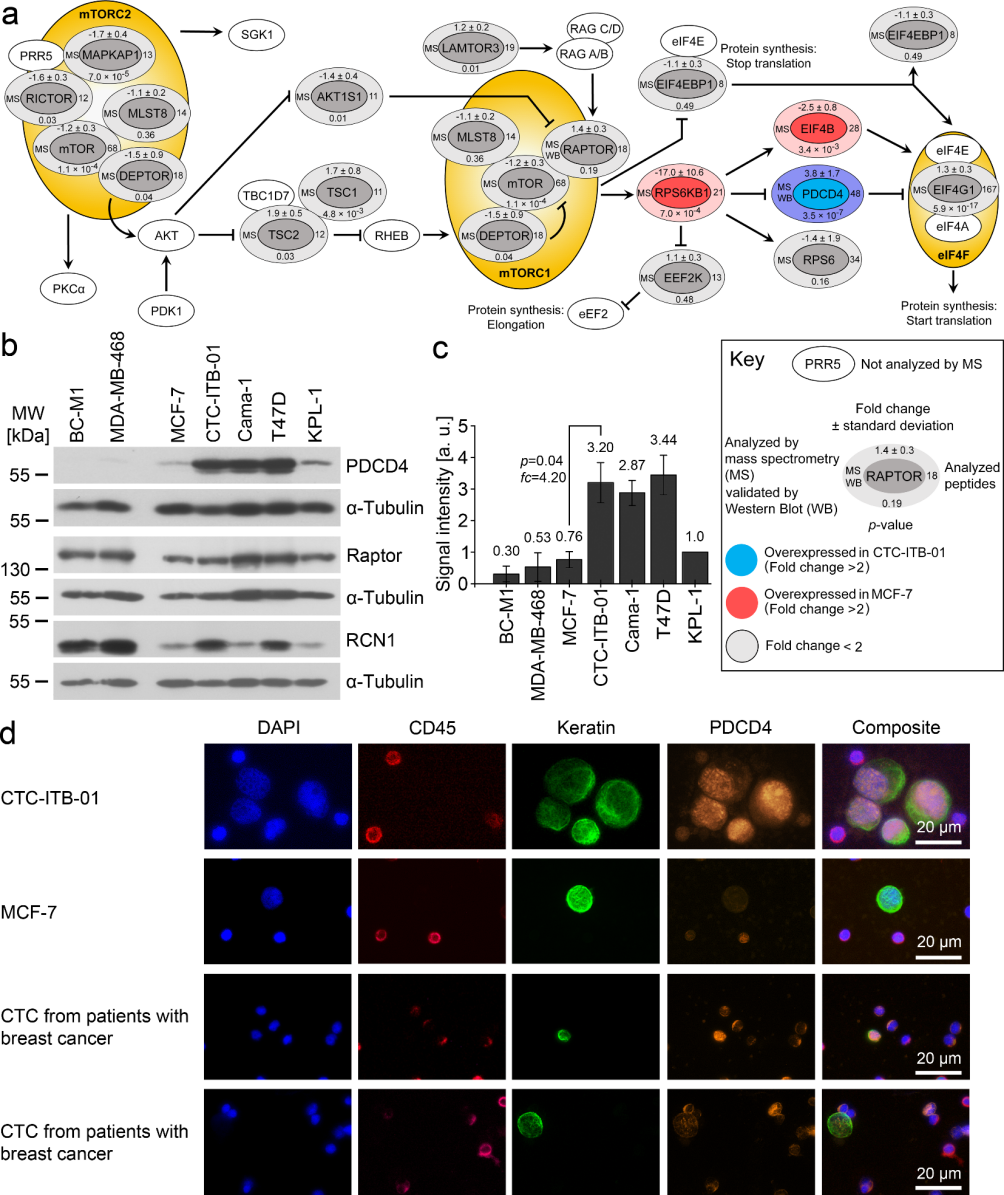
Detection and validation of differentially expressed proteins between CTC-ITB-01 vs. MCF-7.**A**: Analysis of the mTOR pathway based on SILAC-based LC-MS/MS proteomic data. Synonyms: MAPKAP1: SIN1, MLST8: GβL, AKT1S1: PRAS40, RPS6KB1: P70S6K1. For detailed values of the protein identification and quantification, see Supplementary Table S3.**B**: Western Blot analysis of the denoted proteins (*n* = 3). BC-M1 was selected as an example of a disseminated tumor cell line from the bone marrow of a breast cancer patient. MDA-MB-468 was selected as an example of a triple negative breast cell line.**C**: Quantitative Western Blot analysis of PDCD4 (*n* = 3). The average value is shown as numbers and the standard deviation as vertical error bars. The *p*-value was calculated using Student’s two-sided t-test with *p* < 0.05 considered significant.**D**: Detection of PDCD4 in tumor cells of breast cancer patients (*n* = 3). The composite images show overlays of the DAPI, CD45, keratin and PDCD4 signals. Uncropped X-ray film images are shown in supplementary file F1.

Unlike the targeted search for transmembrane proteins, a pathway analysis may embed differentially expressed proteins into a larger functional context. We could compile the majority of proteins of the mTOR pathway for CTC-ITB-01 and MCF-7 (Fig. 3A, Supplementary Table S3). Only RPS6KB1 (ribosomal protein S6 kinase beta-1, also known as P70S6K1, p70 S6 kinase) and eukaryotic translation initiation factor 4B (EIF4B) were significantly higher abundant in MCF-7 compared to CTC-ITB-01. In contrast, programmed cell death protein 4 (PDCD4) was significantly higher abundant in CTC-ITB-01 compared to MCF-7. We confirmed the differential expression of PDCD4 between CTC-ITB-01 and MCF-7 by Western Blot (Fig. 3B and C), and we also detected elevated PDCD4 levels in Cama-1 and T47D. For additional validation of the SILAC results, we selected Raptor as a protein of the mTOR pathway. Finally, we confirmed the differential expression of reticulocalbin-1 (RCN1), which is endoplasmic reticulum resident protein (Fig. 3B, Supplementary Figure S4).

We next investigated the presence of PDCD4 in CTC of breast cancer patients (Fig. 3D). The staining was established using CTC-ITB-01 and MCF-7 as test cell lines. The PDCD4 levels varied in CTC in patients with breast cancer, and we found both PDCD4 positive and PDCD4 negative CTC in the blood of the same patient (*n* = 3).

### Phenotypic changes after modification of SUSD2 levels

**Fig. 4:**
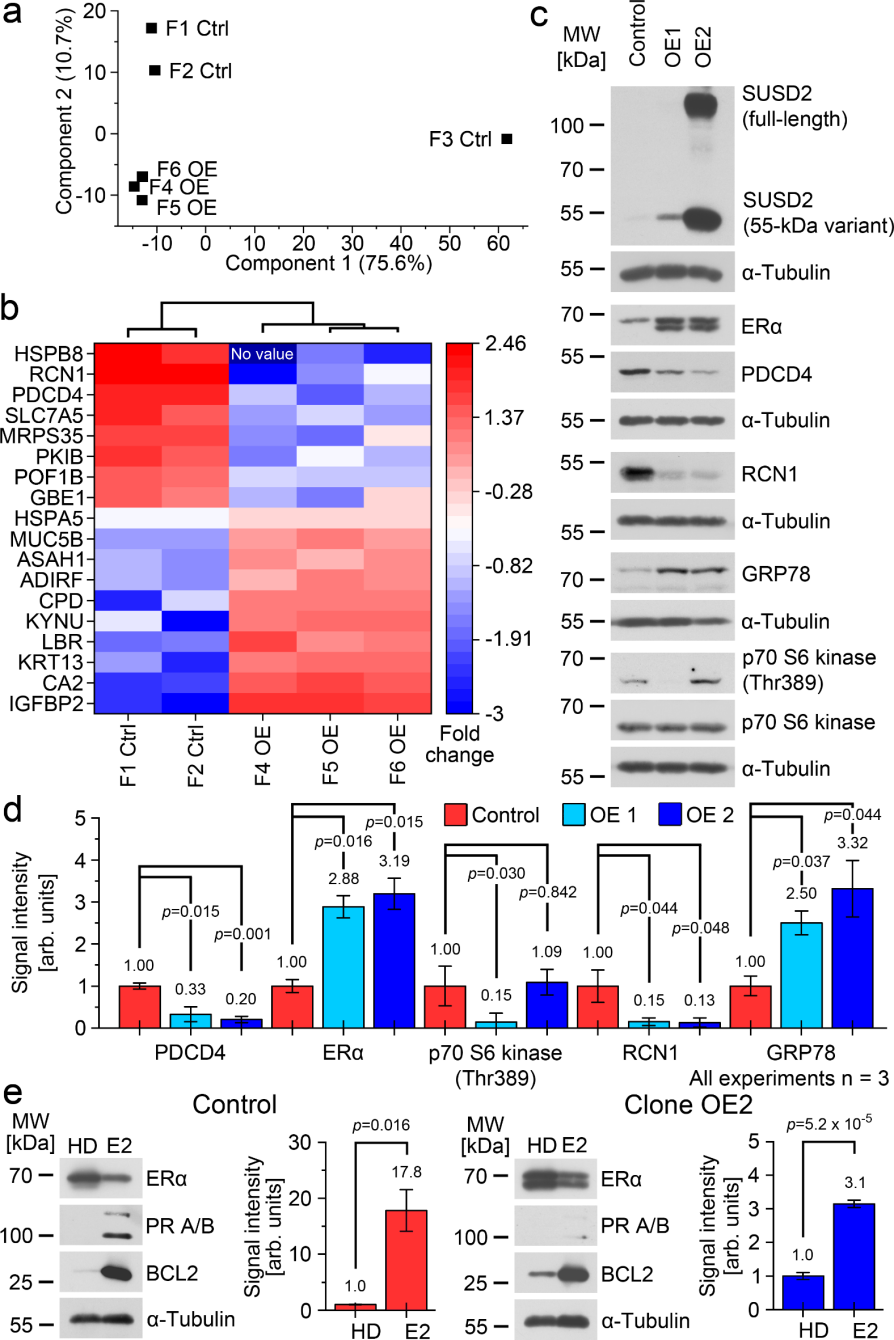
Analysis of the heterologous SUSD2 overexpression in MCF-7.**A**: Principal component analysis of LC-MS/MS data from the three samples without SUSD2-insert (Ctrl) and carrying the SUSD2 gene in the vector (OE, overexpression). Clone OE2 was analyzed.**B**: Visualization of a Pearson-correlation based supervised hierarchical clustering. The color legend displays the log2 foldchanges. Analyzed peptides: 24 (PDCD4), 5 (RCN1), 144 (GRP78, HSPA5). For all proteins, the differential expression was > 2 and significant (*p* < 0.05, Student’s *t*-test).**C**: Western Blot analysis of the control cell line and two SUSD2 overexpressing clones for the denoted proteins.**D**: Quantitative Western Blot analyses of the X-ray films shown in (**C**). The signals were normalized to α-tubulin, in case of p70 S6 kinase (Thr389) the signals were normalized to total p70 S6 kinase. Detailed information is shown in Supplementary Table S4.**E**: Response of the cells to treatment with 17β-Estradiol. Cells were cultured in hormone depleted medium (HD) or in presence of 100 nmol/ml 17β-Estradiol (E2). The diagrams show the quantitative analysis of the BCL2 levels (left: control, right: clone OE2). PR A/B: progesterone receptor A/B). **D**,**E**: Average values are shown as numbers, and the standard deviation as vertical error bars. The *p*-values were calculated using Student’s two-sided t-test where *p* < 0.05 was considered significant. Three biological replicates were analyzed (A-E). Uncropped X-ray film images are shown in supplementary file F1.

Next, we overexpressed SUSD2 in MCF-7 (clones OE). The proteome profile was compared with MFC-7 carrying the vector without insert (MCF-7 control) by label-free quantification based LC-MS/MS analysis. In total, 3102 proteins were analyzed. One biological replicate of MCF-7 control was excluded from further analysis due to its large deviation after principal component analysis (F3 Ctrl; Fig. 4A). The overall protein abundance of F3 Ctrl was lower compared with the other five samples. For the remaining five biological samples the differential proteomic data analysis revealed a set of significantly (2 fold change; *p* < 0.05) differentially abundant proteins between the SUSD2 overexpressed samples and the empty vector control samples (Fig. 4B). The results of the GSEA for this experiment is shown in the Supplementary Figure S5. We used this protein set as a guideline for protein validation by Western Blot (Fig. 4C). The validation comprised two different SUSD2 overexpressing clones. Albeit on low level, clone OE1 is solely positive for the 55-kDa variant suggesting that only the posttranslationally processed SUSD2 is present in OE1, which looks similar to the phenotype observed in MDA-MB-468 (Fig. 2C). The clone that was subjected to LC-MS/MS analysis was OE2. OE2 displays high levels of both the full-length SUSD2 and the 55-kDa variant, which resembles to the SUSD2 phenotype of CTC-ITB-01. Western Blot analysis revealed downregulation of PDCD4 and RCN1 and upregulation of GRP78 (78 kDa glucose-regulated protein) in both clones compared with the vector control (Fig. 4C and D, Supplementary Table S4). Moreover, both clones showed an elevated level of ER-α compared with the control with the formation of an additional band at slightly lower mass. Sensitized by the analysis of the mTOR pathway (Fig. 3A), we analyzed p70 S6 kinase (Fig. 4C and D). We observed reduced phosphorylation of phospho-p70 S6 kinase in clone OE1, but not in OE2 compared with the control. The analysis of other proteins, including AKT and ErbB-2 showed no statistically significant changes (Supplementary Figure S6, Supplementary Table S4).

We investigated how OE2 responds to treatment with 17β-estradiol. To test this, we cultured the control cells and OE2 in hormone depleted medium and treated the cells with 17β-estradiol (Fig. 4E). The control cells displayed the expected normal reaction of MCF-7 cells^13^ by downregulation of ER-α, induction of progesterone receptor A/B and induction of apoptosis regulator BCL2 (fold change 17.8), which is a downstream target of ER-α. OE2 responded considerably weaker to 17β-estradiol by minimal induction of progesterone receptor A/B and induction of BCL2 by a fold change of only 3.1 (Fig. 4E).

**Fig. 5:**
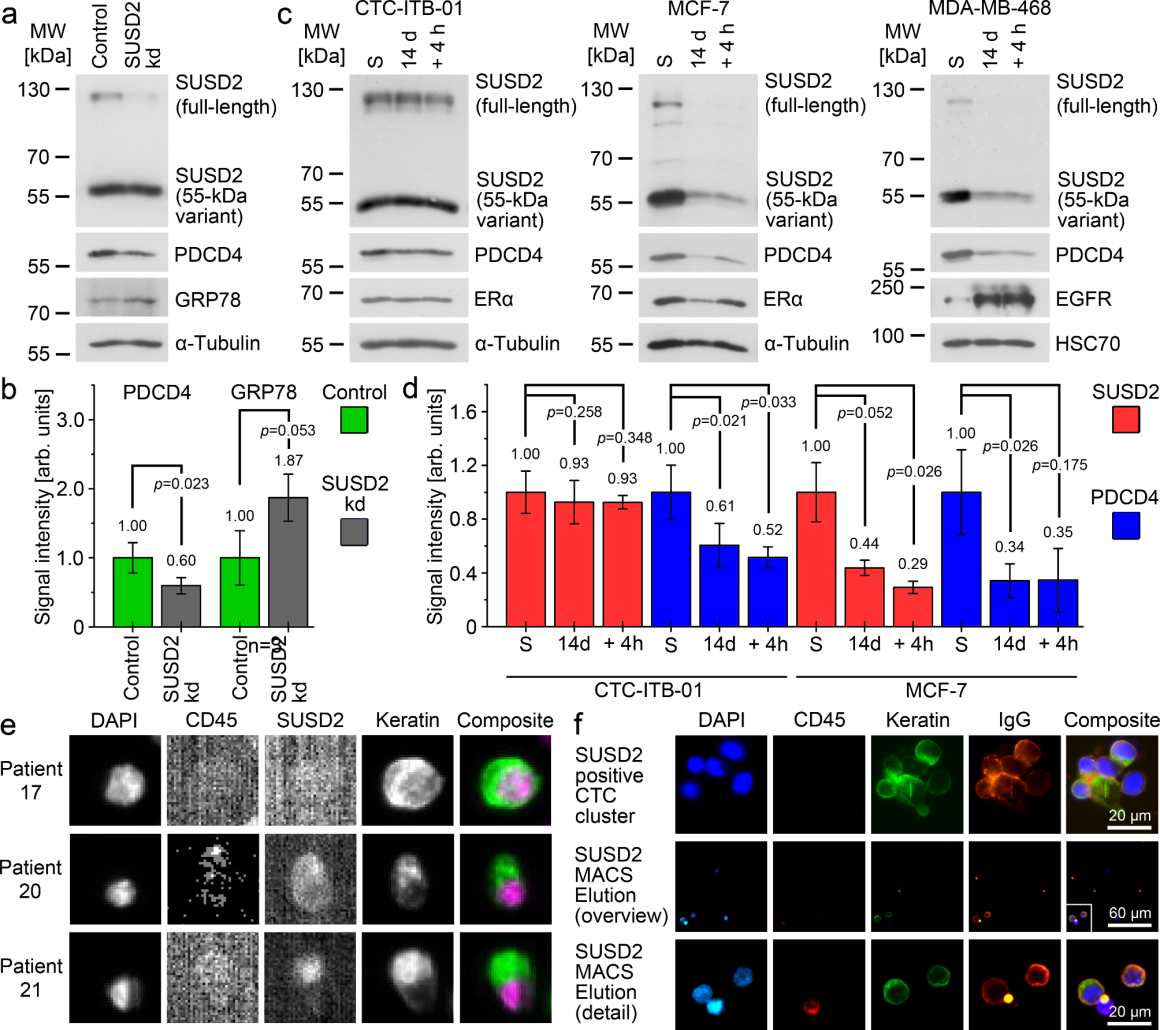
Modification of SUSD2 levels and detection of SUSD2 in CTC of breast cancer patients.**A**: Downregulation of the SUSD2 protein in CTC-ITB-01 by siRNA, analyses by Western Blot.**B**: Quantitative Western Blot analysis of the PDCD4 and GRP78 levels shown in (A).**C**: Response of SUSD2 and PDCD4 to 14 days of hypoxia (1% O_2_; 14 d) and reoxygenation (14 days of hypoxia, followed by 10% O_2_ for 4 h; + 4 h). ERα (MCF-7) and EGFR (MDA-MB-468) were analyzed as an established reaction to hypoxia. S: standard cell culture condition.**D**: Quantitative Western Blot analysis of the SUSD2 and PDCD4 levels shown in (C).**E**: Detection of SUSD2 in CTC of breast cancer patients using the CellSearch system.**F**: Isolation of SUSD2 positive CTCs from the blood of breast cancer patients by MACS. Slides were stained with anti-mouse IgG to detect the MicroBeads bound tumor cells (IgG). The top row shows a SUSD2 positive CTC cluster isolated by MACS. The middle row shows an overview of an elution fraction on a cytospin. The SUSD2 positive CTCs are labelled with the white box and are enlarged in the bottom row.**A**-**D**,**F**: All experiments were performed in biological triplicates. B, D: Average values are shown as numbers, and the standard deviation as vertical error bars. The p-values were calculated using Student’s two-sided t-test where *p* < 0.05 was considered significant.**E**: The CellSearch Results are specified in Supplementary Table S5 (**E**). Uncropped X-ray film images are shown in supplementary file F1.

Moreover, we performed a transient downregulation of SUSD2 expression in CTC-ITB-01 cells by an siRNA approach (Fig. 5A). Herewith, we downregulated mainly the full-length SUSD2 and observed a downregulation of PDCD4 and increased levels of GRP78 in the treated cells (Fig. 5B). After downregulation of the full-length SUSD2 in CTC-ITB-01, the SUSD2 phenotype resembles to phenotype in the triple negative cell line MDA-MB-468 which is mainly positive for the 55-kDa form of SUSD2 (Fig. 2C).

#### Modeling oxygen concentration changes due to intravasation of CTCs

Next, we have used an in vitro model previously introduced to mimic the oxygen changes that tumor cells face when they travel from a hypoxic tissue environment into the oxygenated blood stream^31^. Tumor cell colonies may be confronted with hypoxia (1% O_2_), whereas the blood has higher oxygen concentrations (10% O_2_). Therefore, tumor cells that are released from hypoxic areas into the blood may experience a reoxygenation pulse. We modelled this by subjection of the cells to hypoxia (1% O_2_) for 14 days before cells were re-oxygenated (10% O_2_) for 4 h, simulating the passage of the cells through the blood^31^. We observed a strong downregulation of SUSD2 under hypoxia that was maintained after reoxygenation in MCF-7 and MDA-MB-468, whereas the SUSD2 levels remained constant during hypoxia and re-oxygenation in CTC-ITB-01 (Fig. 5C and D, Supplementary Figure S7). Similarly, PDCD4 was strongly downregulated under hypoxia and reoxygenation in MCF-7 and MDA-MB-468, whereas such downregulation of PDCD4 was less pronounced in CTC-ITB-01 cells (Fig. 5C and D).

#### Validation of clinical breast cancer samples

Next, we analyzed the SUSD2 expression in CTC of patients with breast cancer. An immunofluorescent staining suitable for the CTC detection using the CellSearch system was established (Supplementary Figure S8). Overall, 51 blood samples of 43 different patients with metastatic breast cancer were analyzed. Of those, samples of 23 metastatic patients were positive for at least one CTC in 7.5 ml blood (Fig. 5E), and from five patients, samples were analyzed at two different time points (Supplementary Table S5). The samples from these five patients were from patients with metastatic breast cancer, thus in a stage where the disease progresses fast. Repeated analysis of the same patients offered the possibility to find changes in the proportion of SUSD2 positive CTCs compared with the number of all intact CTCs. In our small group (*n* = 5) the proportion of SUSD2 positive CTCs compared with all intact CTCs remained constant (*p* = 0.533 for a difference; Student´s paired two-sided *t*-Test), suggesting that a SUSD2 positive CTC phenotype is continuously spread during metastasis.

Seven of the 17 hormone receptor positive cases and two of the five triple negative cases contained at least one SUSD2 positive CTC. The one HER2 positive case was negative for SUSD2 positive CTCs. Hence, 39% of the 23 CTC positive cases or 21% of all 43 analyzed cases contained SUSD2 positive CTCs.

The CellSearch approach is a valuable tool for screening of patient samples. However, the signal intensity for SUSD2 was lower by CellSearch compared with direct immunofluorescent staining (Supplementary Figure S8). Therefore, we tried to establish alternative detection approaches with higher signal intensities of the target proteins in CTCs. The large extracellular domains of SUSD2 offers the possibility of CTC catching from the blood by MACS using their extracellular domain for antibody binding. Peripheral blood mononuclear cells (PBMCs) were negative for SUSD2 by Western Blot (Supplementary Figure S9), which is an important prerequisite for CTC isolation by MACS (Supplementary Figure S10A). Quality testing of the MACS by spiking 50 cells into the blood (Supplementary Figure S10B) yielded a recovery rate of 70.7% for CTC-ITB-01 (positive control) and 3.3% for T47D (negative control). The SUSD2-MACS was applied to blood samples of eight patients with metastatic breast cancer. Three samples contained no CTCs, two samples contained only SUSD2-negative CTCs, and in three samples a total of 46 SUSD2-positive CTCs were detected (Fig. 5F).

**Fig. 6:**
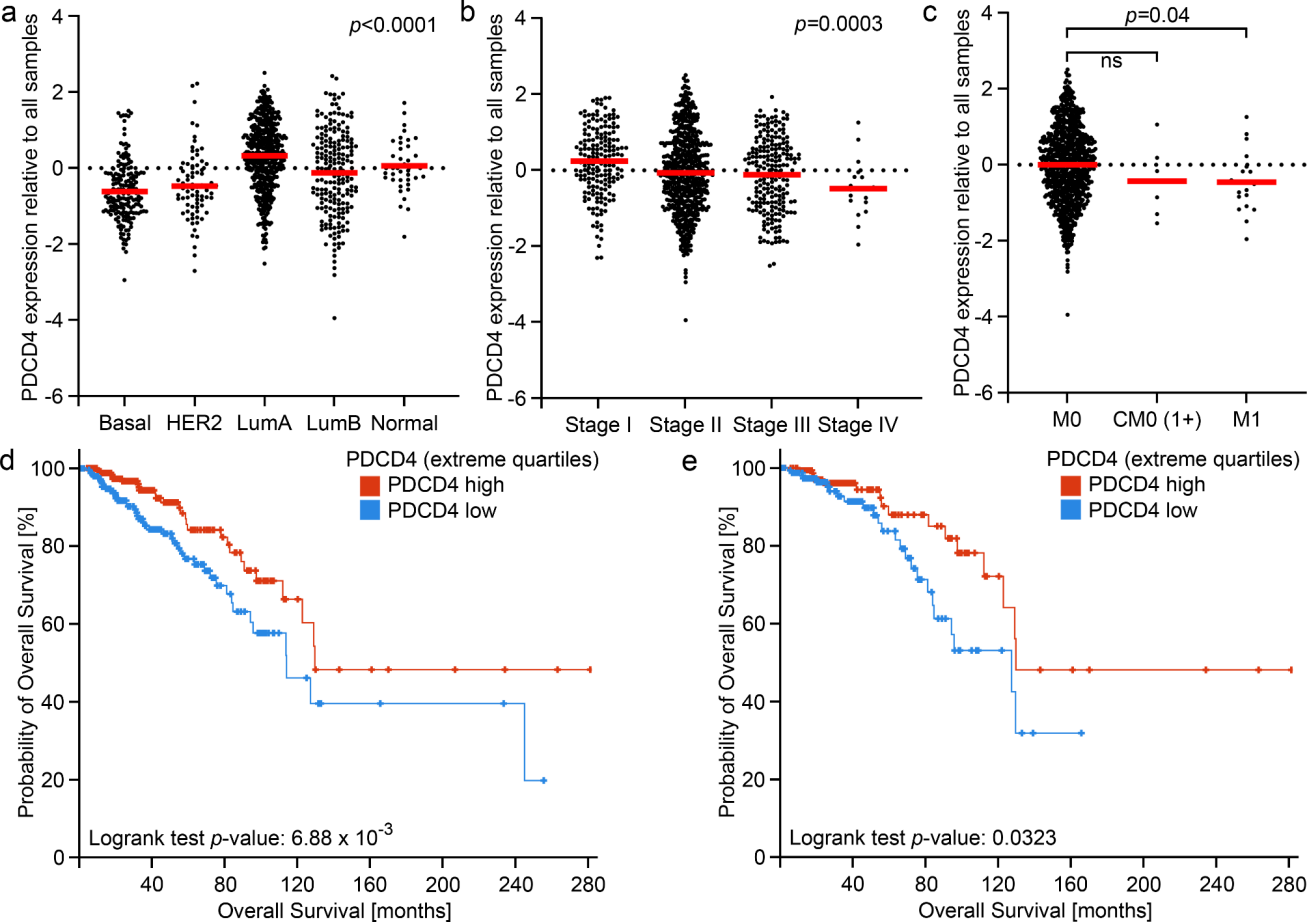
Correlation of PDCD4 levels with clinicopathological data. The data were taken from the breast invasive carcinoma TCGA PanCancer Atlas data set.**A**: Correlation with breast cancer subtypes (basal: *n* = 171, HER2 *n* = 78, LumA, luminal A: *n* = 499, LumB, lumimal **B**:*n* = 197, normal: *n* = 36). B: Correlation with staging (stage I: *n* = 180, stage II: *n* = 615, stage III: *n* = 213, stage IV *n* = 19).**C**: Correlation with dissemination status (M0: *n* = 895, CM0, M0 with CTC in the blood (cM0(1+)): *n* = 6, M1: *n* = 21).**D**: Survival analysis of all breast cancer patients. PDCD4 high: *n* = 271, PDCD4 low: *n* = 271. E: Survival analysis of the luminal subtypes. PDCD4 high: *n* = 174, PDCD4 low: *n* = 174.

Since modulation of SUSD2 levels in cell lines resulted in downregulation of PDCD4, we searched The Cancer Genome Atlas (TCGA)^29,30^ for clinic-pathological data of PDCD4 in breast cancer. The highest PDCD4 levels were present in Luminal A primary breast cancers, whereas basal breast cancers displayed the lowest PDCD4 levels (Fig. 6A). Moreover, PDCD4 levels decreased with increased staging (Fig. 6B) and with metastasis (M0 vs. M1, Fig. 6C). Analysis of the overall survival of all breast cancer cases revealed a significant (*p* = 6.88 × 10^− 3^) reduced survival in cases with low PDCD4 levels (Fig. 6D). Focusing on luminal subtypes only, patients with low PDCD4 levels displayed a significant (*p* = 0.0323) reduced survival (Fig. 6E).

## Discussion

CTC-ITB-01 offers the exceptional possibility to gain insights into the proteome of CTCs from very late stage breast cancer. While usual CTC approaches detect just one (or few) proteins in a number of patients (usually without quantification), the proteome analysis of CTC-ITB-01 reported here provides the quantification of almost 8,000 proteins. MCF-7 is available world-wide and its cultivation is unproblematic, so that every researcher can use our quantitative SILAC data using MCF-7 as an in-house reference. If the relative protein expression value for a given protein is determined in MCF-7 in any laboratory world-wide, one can calculate the corresponding expression value in CTC-ITB-01 using the SILAC fold change we deposited in the publicly accessible database. Apart from targeted protein analyses, it is possible to compare the entire proteomic data set we deposited using a tool we have developed^32^. The inherent limitation of this approach is having analyzed the CTC population of only one patient, which required the presented validation on clinical samples from additional patients.

Even though previous research has clarified some aspects about SUSD2 in breast cancer^16,33^, it remained unclear if SUSD2 positive cells can be released into in the blood stream. Here, we found that 39% of CTC positive cases contained SUSD2-positive CTCs. Whether these SUSD2-positive cells represent a more aggressive subset of CTCs with metastasis-initiating capacities remains subject of future investigations.

Moreover, the large extracellular domain of SUSD2 was suitable for the specific enrichment of SUSD2 positive CTC by MACS. This approach allows now in future studies the characterization of SUSD2-positive CTCs by molecular genetic approaches. The present observation that SUSD2 expression remained unchanged in our hypoxia/re-oxygenation experiments suggests that SUSD2 might be a stable CTC marker reflecting the expression in the tumor tissue(s).

In a general image, it appeared that the originating tumor cells of CTC-ITB-01 were at least temporarily confronted with adverse microenvironmental conditions, which also had an effect on SUSD2. The maturation of SUSD2 results in a heterodimer comprising two interconnected polypeptide chains of SUSD2, which is transported to the cell surface and interacts there with galectin-1^15^. The exact mode of interaction is not clear; probably SUSD2 is either a chaperone for galectin-1 or it sequesters galectin-1^16^. If located on the cell surface, galectin-1 can introduce T cell apoptosis, so that SUSD2 contributes to the evasion of tumor cells from targeting by the immune system^15,34^.

We have found very high levels of SUSD2 in CTC-ITB-01, which principally enables this cell line to use galectin-1 for escape from immune surveillance. However, the endogenous galectin-1 synthesis is very low, so that CTC-ITB-01 is dependent on external galectin-1 sources. Since CTC-ITB-01 was derived from CTCs of the blood circuit, either the surrounding tissue where CTC-ITB-01 has resided before dissemination or the blood itself are potential sources where it can obtain external galectin-1. In healthy individuals, a galectin-1 serum concentration of 8.9 ± 1.3 ng/ml was reported^35^, which might be one source for galectin-1 of CTC-ITB-01. Since the expression of PD-L1 is very low in CTC-ITB-01, the SUSD2/galectin-1 complex might be an alternative for the escape from immune recognition.

Under such circumstances, CTC-ITB-01 is strongly dependent on a continuous expression of SUSD2, even under adverse microenvironmental conditions. While MCF-7 and MDA-MB-468 strongly downregulate SUSD2 under hypoxic conditions, SUSD2 levels remained constant in CTC-ITB-01. This points towards the acquisition of regulatory changes in CTC-ITB-01 to maintain high levels of SUSD2 under hypoxia for survival. It appears to be unlikely that CTC-ITB-01 has acquired these regulatory changes during its passage through the blood, since the half-life of CTCs in the blood is with 2–3 h^5^ by far too short. Moreover, such an optimization process is more efficient when phenotypic advantages occur, which requires the presence of galectin-1 in the near microenvironment. At the time of blood sampling, the breast cancer patient from which CTC-ITB-01 was derived, had metastases in the lymph nodes, bones, spleen, liver and vagina^13^ Among those, galectin-1 mRNA is detectable in considerable amounts in the spleen and the liver of healthy persons^36^. In the normal spleen, oxygen concentrations of approx. 1–6%, and in the normal liver oxygen concentrations of 1–12% are detected^37^, which might contain starting conditions for a metastasis that is confronted with low oxygen concentrations during outgrowth. Hence, both organs might be considered as the originating tissue, which harbored the metastasis where tumor cells changed the SUSD2 regulation towards stable SUSD2 expression under hypoxia. Later on some of the cells disseminated into the blood and became the ancestors of CTC-ITB-01. This assumption is supported by analyses of copy number alterations where samples of CTC-ITB-01 DNA cluster separately to the original primary tumors and the vaginal metastasis^13^ so that these tissues are unlikely originating sites.

The maturation of SUSD2 results in a heterodimer comprising two interconnected polypeptide chains of SUSD2, which is transported to the cell surface and interacts there with galectin-1.

Subsequently, we performed functional in vitro studies to assess the role of SUSD2. Remarkably, PDCD4 appeared as prominent target of SUSD2 with a dual role depending on the cellular background. PDCD4 inhibits the initiation of the protein translation by binding to eukaryotic initiation factor 4 A (eIF4A) thereby linking the mTOR pathway with the control of the protein synthesis machinery^38^. In tumor cells, PDCD4 inhibits the neoplastic transformation, thus PDCD4 is regarded as a tumor suppressor^39^. Clinically, low levels of PDCD4 are associated with poor prognosis in hormone receptor-positive breast cancer^40,41^.

In our experiments, PDCD4 was downregulated after SUSD2 overexpression (MCF-7), SUSD2 downregulation by siRNA (CTC-ITB-01) and also by hypoxia (CTC-ITB-01, MCF-7, MDA-MB-468). Albeit a triple negative cell line, MDA-MB-468 is positive for the processed 55-kDa SUSD2, suggesting that these effects can be also relevant beyond the ER-α positive subtype. The observed relation between SUSD2 and PDCD4 might be due to different underlying mechanisms disturbing cellular homeostasis.

In that context, decrease of PDCD4 levels enables the synthesis of proteins by the cap-independent translation^42^. Second, alteration of the SUSD2 levels leads to perturbation of the endoplasmic reticulum homeostasis as seen by the induction of the endoplasmic stress sensor GRP78 in OE1 and OE2, as well in the SUSD2 kd cells of CTC-ITB-01. A contribution of PDCD4 to an endoplasmic reticulum stress response with the activation of GRP78 appears to be reasonable, since these programs frequently overlap with the mTOR pathway, for example under hypoxia^43^.

Since we could not find a previous report about the presence of PDCD4 in CTCs, we confirmed PDCD4 in CTCs of breast cancer patients in our work. It turned out that the distribution of PDCD4 in CTCs displayed a considerable intra-patient heterogeneity. The detection of PDCD4 low expressing CTCs may be due to the downregulation of PDCD4 in hypoxic areas of tumor cell colonies, followed by release of such CTCs into the blood. In the ground state, the PDCD4 expression is 4.2 fold higher in CTC-ITB-01 than in MCF-7, and under hypoxia the PDCD4 level decreased to about 30% in MCF-7. That would make a range of factor 14 from normoxic CTC-ITB-01 to hypoxic MCF-7. If a patient contains cancer cells like MCF-7 and CTC-ITB-01 at different sites, which are subjected to hypoxia to varying degrees, then these conditions may explain the variability of the PDCD4 levels in CTCs of the patients.

The exact molecular mechanism how SUSD2 affects PDCD4 levels remains to be investigated. In colorectal cancer, PDCD4 inhibits SIN1 (MAPKAP1, Fig. 3A) translation, to reduce cell invasion^44^. SIN1 is a critical component of mTORC2, and inhibition of SIN1 translation by PDCD4 leads to inactivation of AKT^40^. We observed a downregulation of pAKT (S473) in OE1 and OE2, but this change did not reach statistical significance due to large deviations in the biological replicates.

Another interesting protein affected by overexpression of SUSD2 was GRP78, a chaperone resident in the endoplasmic reticulum that supports the proper folding of nascent proteins and belongs to the cytoprotective program of the unfolded protein response (UPR). Previously, we found that GRP78 supports the survival of disseminated tumor cells in the bone marrow of breast cancer patients^17^. In the function of re-establishing cellular homeostasis, GRP78 exhibits anti-apoptotic activity^45^ or mediates resistance to chemotherapy, including to anti-estrogen resistance^45,46^. These findings might suit to our finding of downregulation of RCN1 after the overexpression of SUSD2 in MCF-7. Few is known about the role of RCN1 in cancer, yet downregulation of RCN1 has been implicated in the suppression of apoptosis after activation of the UPR^47^.

After SUSD2 overexpression in MCF-7, we observed a twin band for ER-α in OE1 and OE2. Several mRNA splice variants of ER-α have been described varying in the molecular mass of the synthesized protein^48^, of which the variant ER-αδE3 (62.3 kDa) is only 4.3 kDa smaller than the wild type ER-α (66.6 kDa), which suits best to our findings in OE1 and OE2. ER-αδE3 is unable to bind to the DNA due to partial loss of the DNA binding domain, but the ligand-binding domain is functional^48^. ER-αδE3 is a naturally form of ER-α, and the loss of ER-αδE3 in breast cancer may lead to uncontrolled estrogen stimulation^49^. Artificial overexpression of ER-αδE3 in MCF-7 yielded in a dominant negative ER-α phenotype with diminished tumorigenic attributes^49^. This reduced ability to respond to 17β-estradiol was also visible in our work in OE2 by negligible induction of progesterone receptor A/B and 5.6-fold weaker induction of BCL2 than in the control cells. BCL2 is an anti-apoptotic protein and has been linked to the ER-α positive breast cancer subtype and was associated with favorable outcomes^50^. Since in patients CTCs with higher BCL2 levels correlated with better outcomes^51^, SUSD2 mediated attenuation of BCL2 might complement the pro-survival effect of GRP78 in CTC under therapy conditions.

## Electronic supplementary material

Below is the link to the electronic supplementary material.

Supplementary Material 1

Supplementary Material 2

Supplementary Material 3

## Data Availability

All data generated or analysed during this study are included in this published article (and its Supplementary Information files). The mass spectrometry proteomics data have been deposited to the ProteomeXchange Consortium via the PRIDE^52^  partner repository (https://www.ebi.ac.uk/pride/) with the dataset identifier PXD047444.
