# Supplementary material for: Discovery of a sushi domain-containing protein 2-positive phenotype in circulating tumor cells of metastatic breast cancer patients
Source: Sci Rep. 2025 Jan 31;15:3913. doi: 10.1038/s41598-025-87122-4 (PMC11785953; doi:10.1038/s41598-025-87122-4)

Discovery of a sushi domain-containing protein 2-positive phenotype in circulating tumor cells of metastatic breast cancer patients

Kai Bartkowiak, Parinaz Mossahebi Mohammadi, Paula Nissen, Stefan Werner, David Agorku, Antje Andreas, Maria Geffken, Sven Peine, Karl Verpoort, Thomas M. Deutsch, Laura L. Michel, Andreas Schneeweiss, Verena Thewes, Andreas Trumpp, Olaf Hardt, Volkmar Müller, Sabine Riethdorf, Hartmut Schlüter, Klaus Pantel

Supplementary Information

This article is accompanied by the following Supplementary Information items:

Supplementary Information File including:

- Supplementary Table S1: Primary antibodies and dilutions used for Western Blot
- Supplementary Table S4: Western Blot analysis details for the SUSD2 overexpressing MCF-7 cell lines
- Supplementary Table S5: Clinicopathological parameters, CTC and SUSD2 detection rates
- Supplementary Figure S1: Relation between fold change and its statistical significance of proteins detected in CTC-ITB-01 and MCF-7
- Supplementary Figure S2: Gene set enrichment plots for the differentially expressed proteins between CTC-ITB-01 vs. MCF-7
- Supplementary Figure S3: Western Blot analysis for technical validation proteins
- Supplementary Figure S4: Quantitative analysis of reticulocalbin-1
- Supplementary Figure S5: Gene set enrichment plots for Clone OE2 vs. control expressed in MCF-7

- Supplementary Figure S6: Western Blot analysis for proteins in the SUSD2 overexpressing clones
- Supplementary Figure S7: Quantitative Western Blot analysis of SUSD2 and PDCD4 in MDA-MB-468
- Supplementary Figure S8: Establishment of the immunofluorescent staining of SUSD2
- Supplementary Figure S9: Validation of SUSD2 in PBMC
- Supplementary Figure S10: Evaluation of the MACS system using SUSD2 MicroBeads
- Uncropped Western Blots

Separate Excel Files:

- Supplementary Table S2: Differential quantitative proteomics result details for test proteins and transmembrane proteins
- Supplementary Table S3: Differential quantitative proteomics result details for the proteins of the mTOR pathway

Supplementary Figures

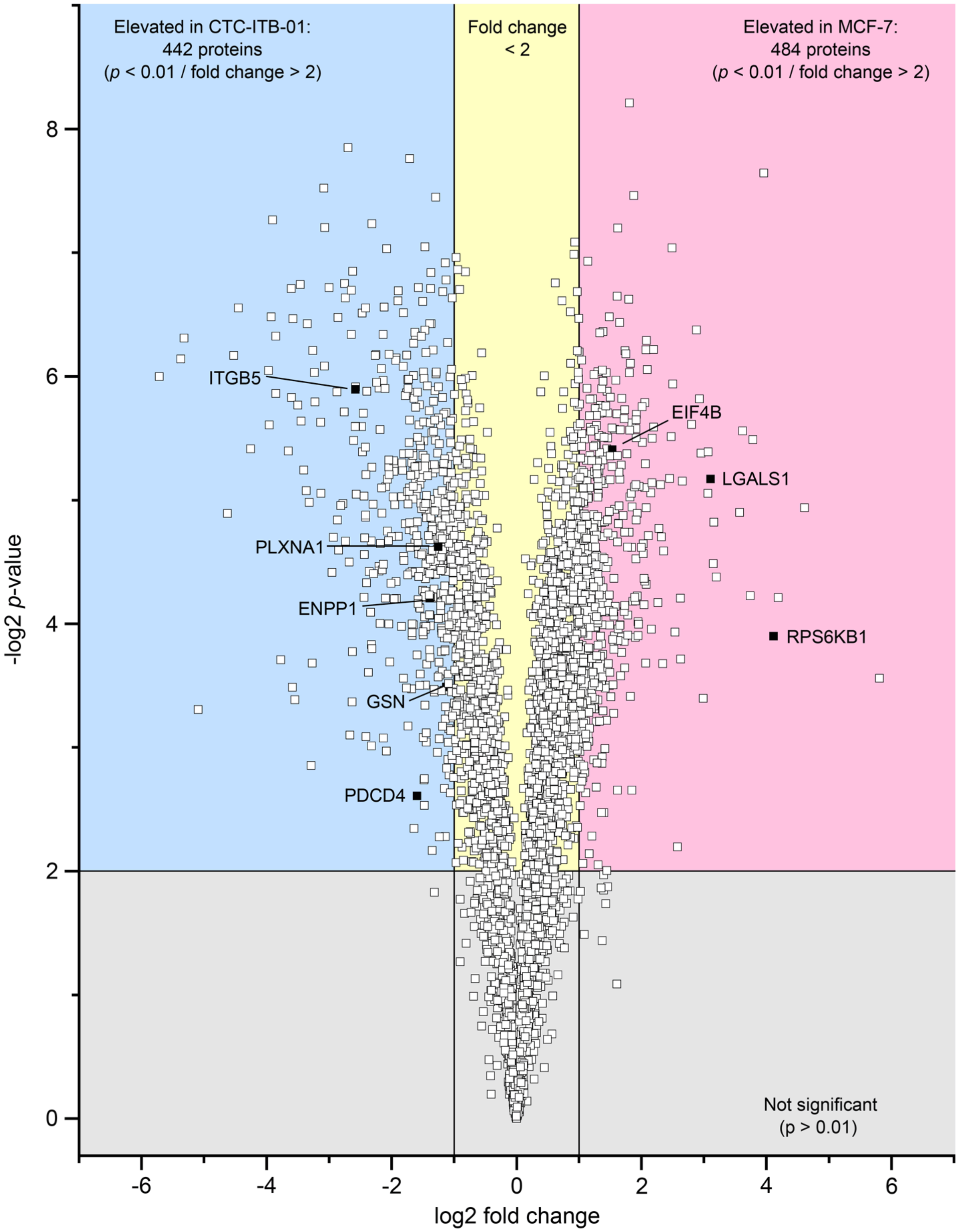

Supplementary Figure S1. Relation between fold change and its statistical significance of proteins detected in CTC-ITB-01 and MCF-7 by SILAC LC-MS/MS. Only proteins that were quantified with at least five peptides are shown (3692 proteins). Significantly differentially

expressed proteins presented in the main text (Table 1 and Figure 3a) are assigned with their gene names**.**

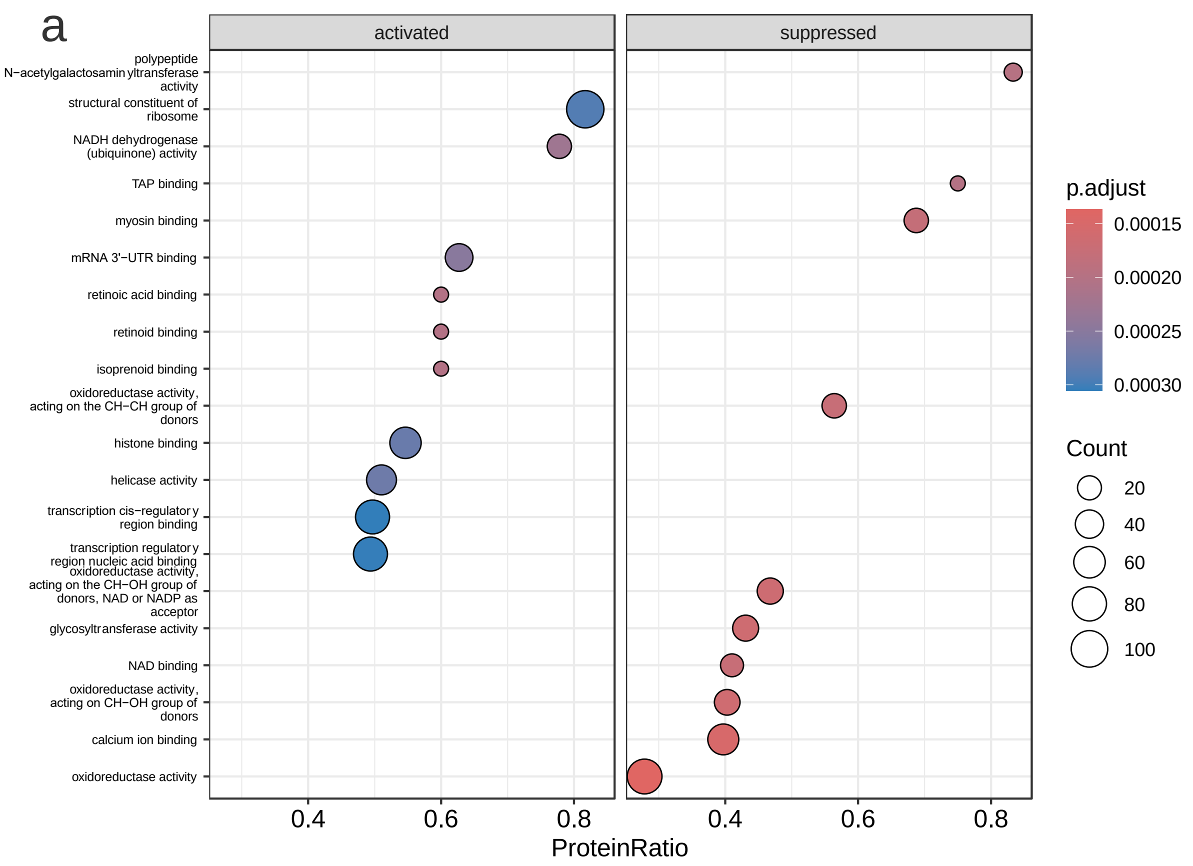
a
activated
suppressed
polypeptide N−acetylgalactosaminyltransferase activity
structural constituent of ribosome
NADH dehydrogenase (ubiquinone) activity
TAP binding
myosin binding
mRNA 3'−UTR binding
retinoic acid binding
retinoid binding
isoprenoid binding
oxidoreductase activity, acting on the CH−CH group of donors
histone binding
helicase activity
transcription cis−regulatory region binding
transcription regulatory region nucleic acid binding
oxidoreductase activity, acting on the CH−OH group of donors, NAD or NADP as acceptor
glycosyltransferase activity
NAD binding
oxidoreductase activity, acting on CH−OH group of donors
calcium ion binding
oxidoreductase activity
0.4
0.6
0.8
ProteinRatio
p.adjust
0.00015
0.00020
0.00025
0.00030
Count
20
40
60
80
100

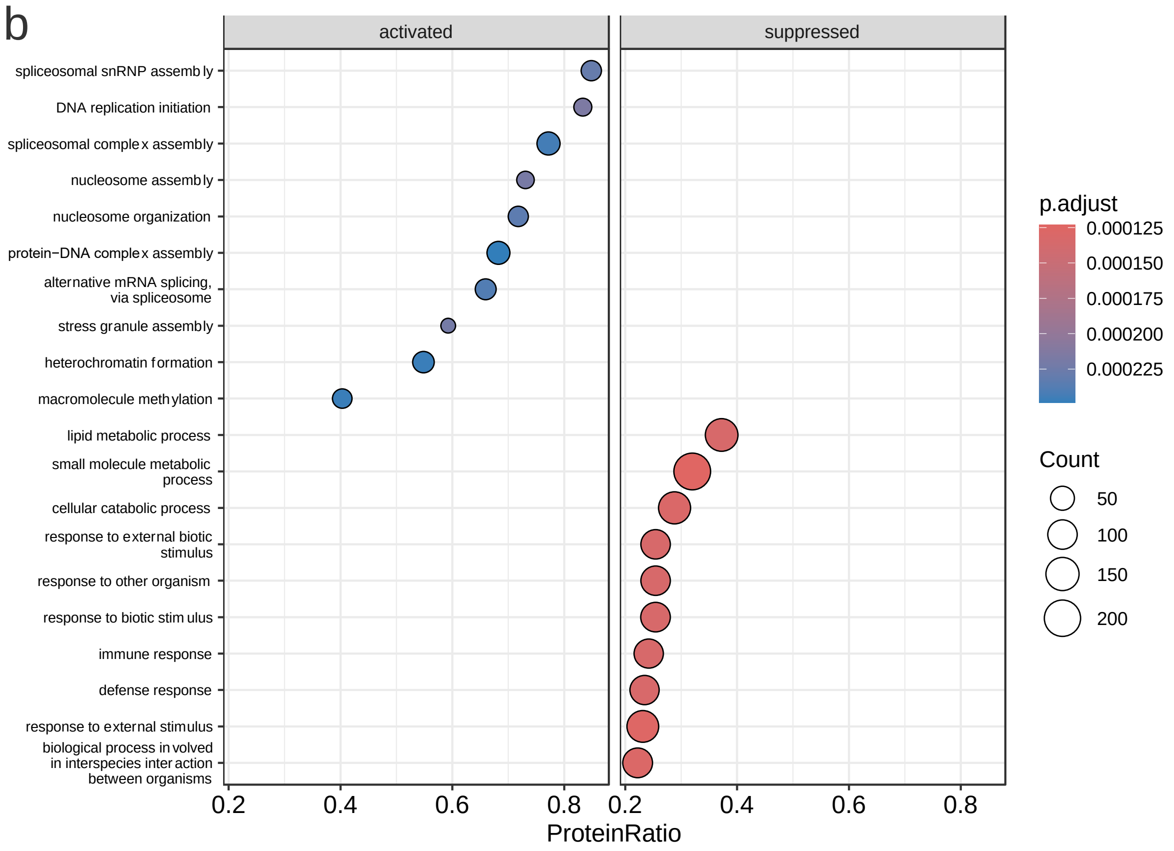
b
activated
suppressed
spliceosomal snRNP assembly
DNA replication initiation
spliceosomal complex assembly
nucleosome assembly
nucleosome organization
protein−DNA complex assembly
alternative mRNA splicing, via spliceosome
stress granule assembly
heterochromatin formation
macromolecule methylation
lipid metabolic process
small molecule metabolic process
cellular catabolic process
response to external biotic stimulus
response to other organism
response to biotic stimulus
immune response
defense response
response to external stimulus
biological process involved in interspecies interaction between organisms
0.2
0.4
0.6
0.8
ProteinRatio
p.adjust
0.000125
0.000150
0.000175
0.000200
0.000225
Count
50
100
150
200

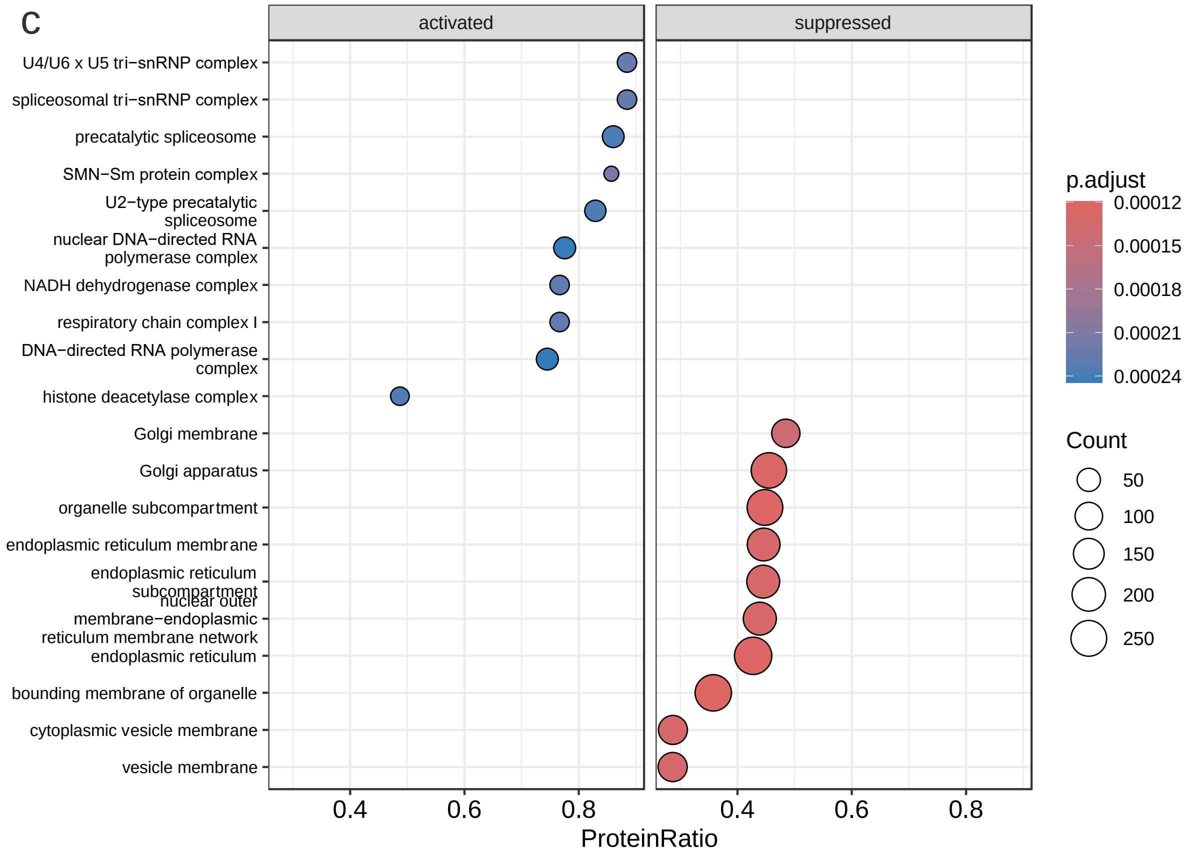

Supplementary Figure S2. Gene set enrichment plots for the differentially expressed proteins between CTC-ITB-01 vs. MCF-7 using SILAC LC-MS/MS. Presented are the results for all T-test significantly abundant proteins (*p*-value < 0.05) between heavy/light ratio and mean with a fold change of more than 2. The gene names were searched against GO molecular function (a) biological pathways (b) and cellular compartments (c) gene sets. Activated: higher intensity in MCF-7 than in CTC-ITB-01. Suppressed: lower intensity in MCF-7 than in CTC-ITB-01. The image was created using RStudio, version 2024.09.1 (https://rstudio-education.github.io/hopr/starting.html).

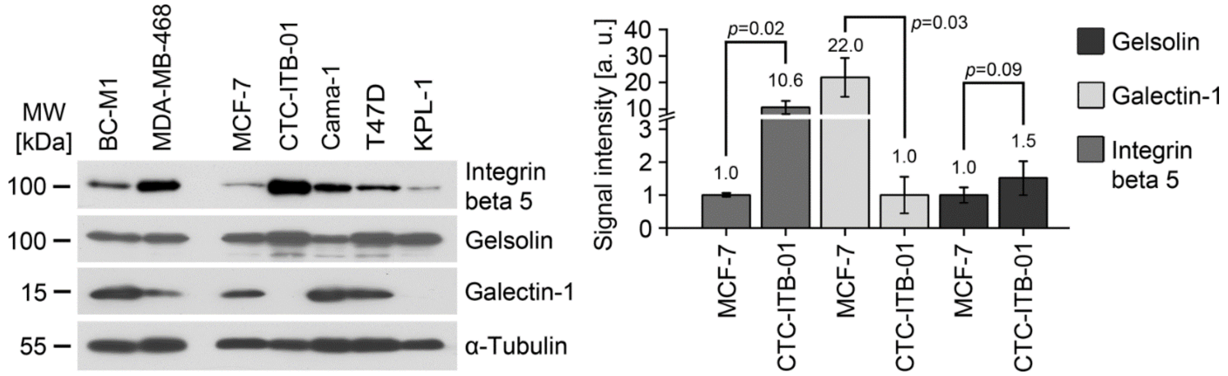

Supplementary Figure S3. Analysis of differentially expressed proteins between MCF-7 and CTC-ITB-01 by Western Blot (left). These proteins were differentially expressed in these two cell lines in the SILAC approach. Quantitative analysis of the signal intensities of the denoted proteins (right). The *p*-values were calculated using Student's two-sided *t*-test where $p<0.05$ was considered significant. For direct assessment of the fold change, the average values were set to 1 for each protein. Vertical error bars show the standard deviation of three independent measurements. Uncropped X-ray film images are shown in supplementary file F1.

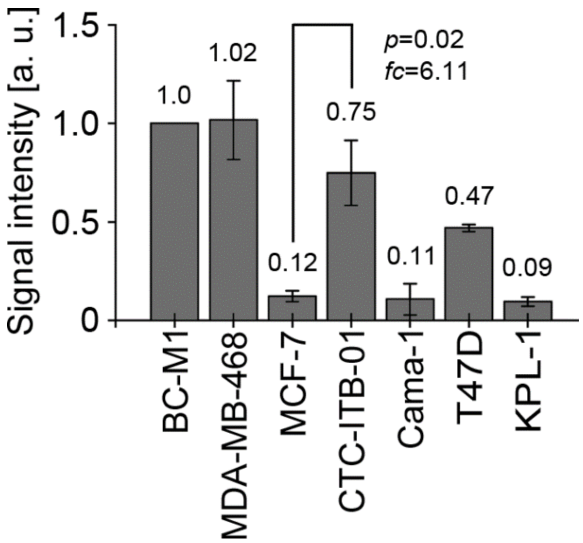

Supplementary Figure S4. Quantitative Western Blot analysis of reticulocalbin-1. Alpha-tubulin served as a loading control. The *p*-value was calculated using Student's two-sided *t*-test where $p<0.05$ was considered significant. For direct assessment of the fold change, the average values were set to 1 for BC-M1. Vertical error bars show the standard deviation of three independent measurements.

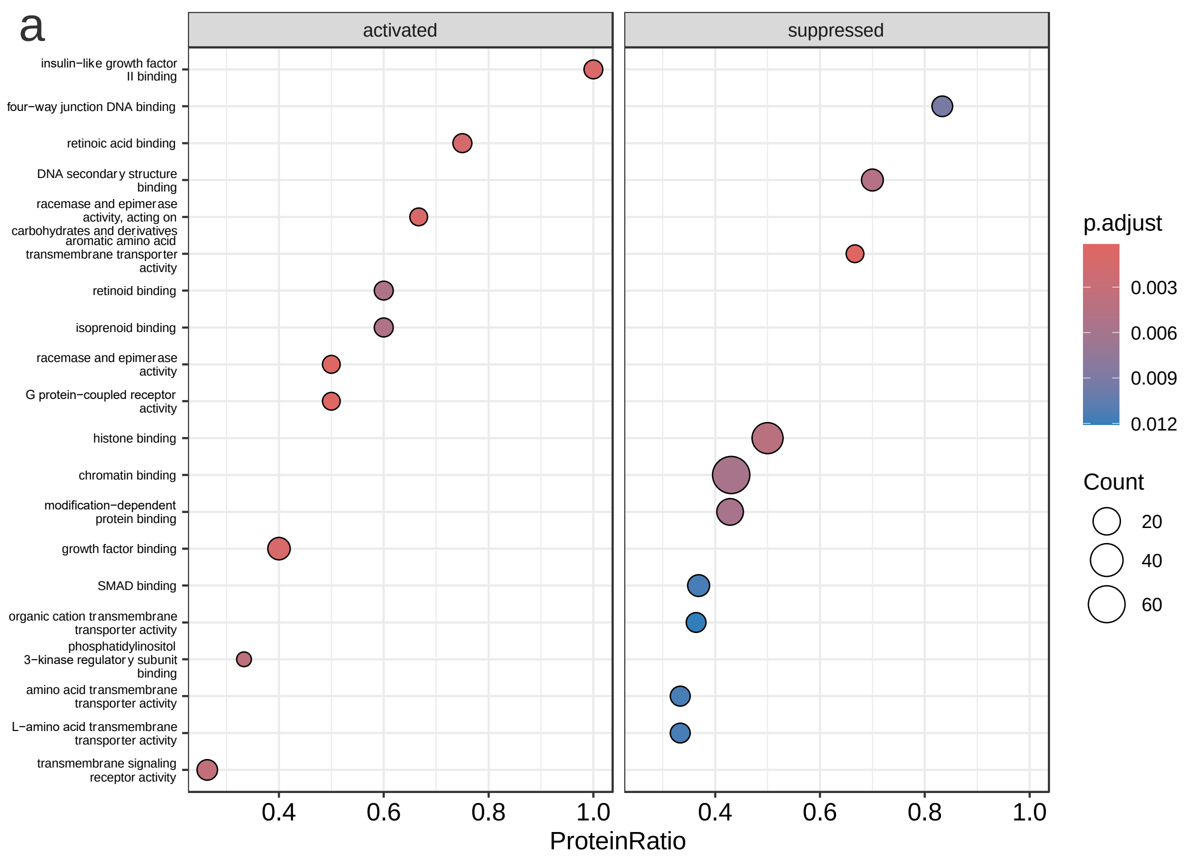
a
activated
suppressed
insulin−like growth factor II binding
four−way junction DNA binding
retinoic acid binding
DNA secondary structure binding
racemase and epimerase activity, acting on carbohydrates and derivatives
aromatic amino acid transmembrane transporter activity
retinoid binding
isoprenoid binding
racemase and epimerase activity
G protein−coupled receptor activity
histone binding
chromatin binding
modification−dependent protein binding
growth factor binding
SMAD binding
organic cation transmembrane transporter activity
phosphatidylinositol 3−kinase regulatory subunit binding
amino acid transmembrane transporter activity
L−amino acid transmembrane transporter activity
transmembrane signaling receptor activity
0.4 0.6 0.8 1.0
0.4 0.6 0.8 1.0
ProteinRatio
p.adjust
0.003
0.006
0.009
0.012
Count
20
40
60

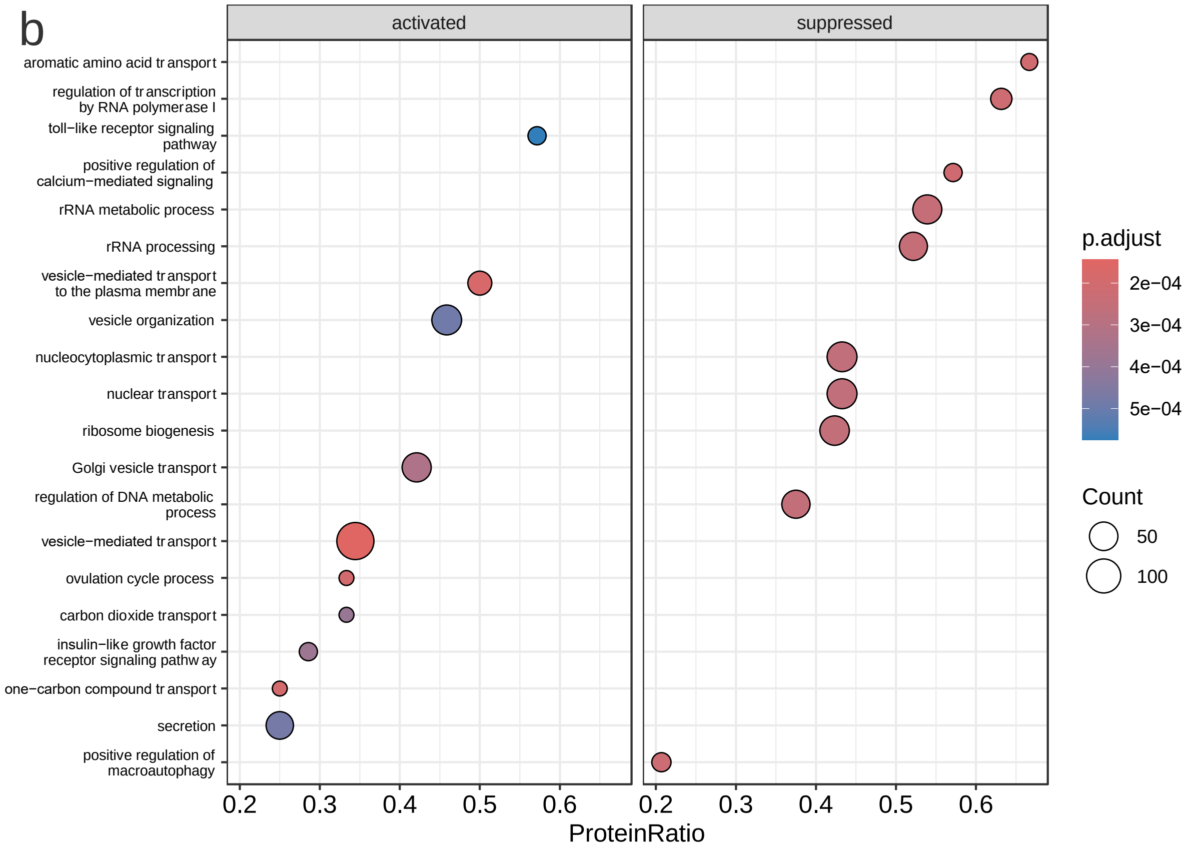
b
activated
suppressed
aromatic amino acid transport
regulation of transcription by RNA polymerase I
toll−like receptor signaling pathway
positive regulation of calcium−mediated signaling
rRNA metabolic process
rRNA processing
vesicle−mediated transport to the plasma membrane
vesicle organization
nucleocytoplasmic transport
nuclear transport
ribosome biogenesis
Golgi vesicle transport
regulation of DNA metabolic process
vesicle−mediated transport
ovulation cycle process
carbon dioxide transport
insulin−like growth factor receptor signaling pathway
one−carbon compound transport
secretion
positive regulation of macroautophagy
0.2 0.3 0.4 0.5 0.6
0.2 0.3 0.4 0.5 0.6
ProteinRatio
p.adjust
2e−04
3e−04
4e−04
5e−04
Count
50
100

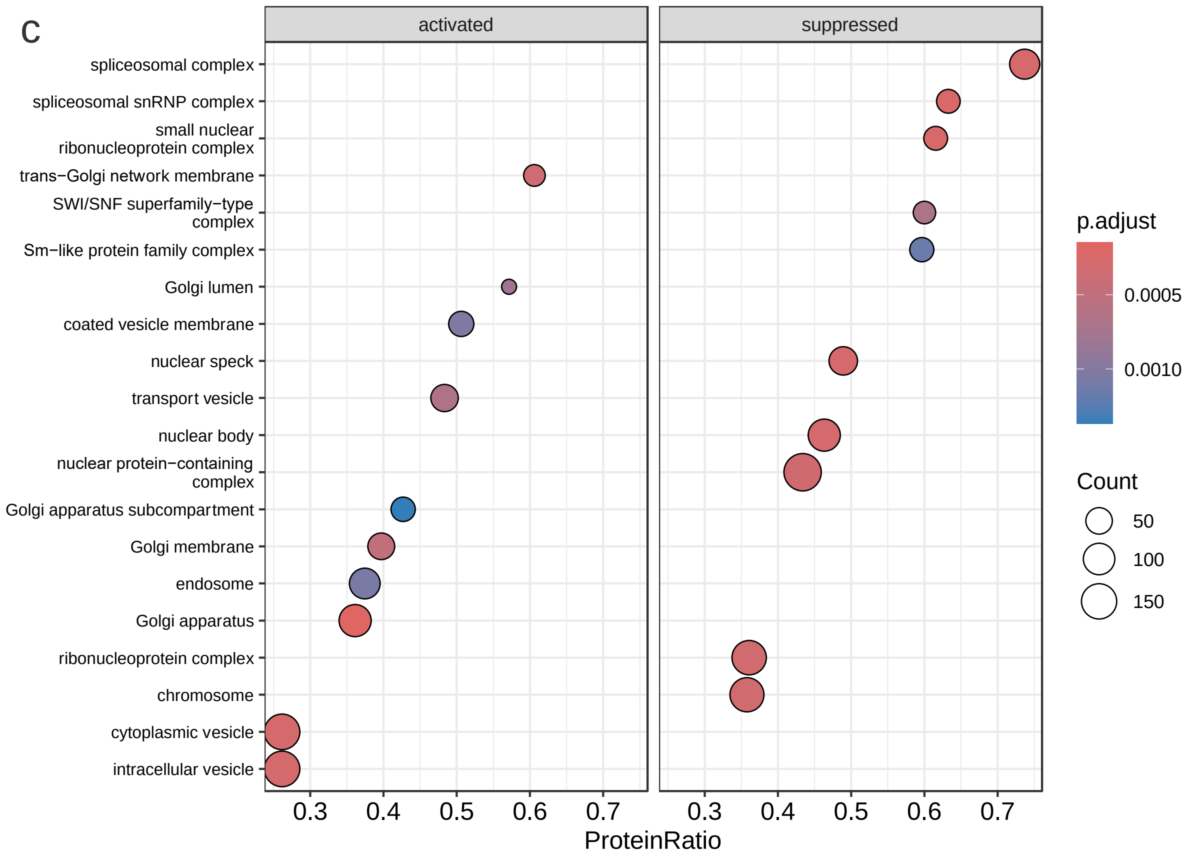

Supplementary Figure S5. Gene set enrichment plots for the differentially expressed proteins between Clone OE2 vs. control expressed in MCF-7 using LFQ LC-MS/MS. Presented are the results for all T-test significantly abundant proteins (*p*-value < 0.05) between the three biological replicates of OE2 and two biological replicates of the vector control with a fold change of more than 1.5. The sample F3 Ctrl (see Figure 4A of the main text) was excluded from the analysis. The gene names were searched against GO molecular function (a) biological pathways (b) and cellular compartments (c) gene sets. Activated: higher intensity in Clone OE2 than in control. Suppressed: lower intensity in Clone OE2 than in control. The image was created using RStudio, version 2024.09.1 (https://rstudio-education.github.io/hopr/starting.html)

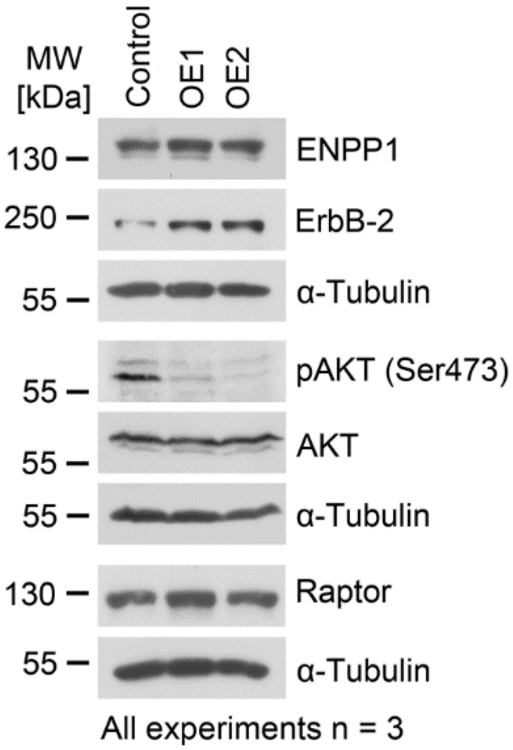

Supplementary Figure S6. Western Blot analysis for proteins in the SUSD2 overexpressing clones of MCF-7 (OE1 and OE2). The control cell line is MCF-7 carrying the SUSD2 vector without insert. Alpha-tubulin served as a loading control. For details, see Supplementary Table S4. Uncropped X-ray film images are shown in supplementary file F1.

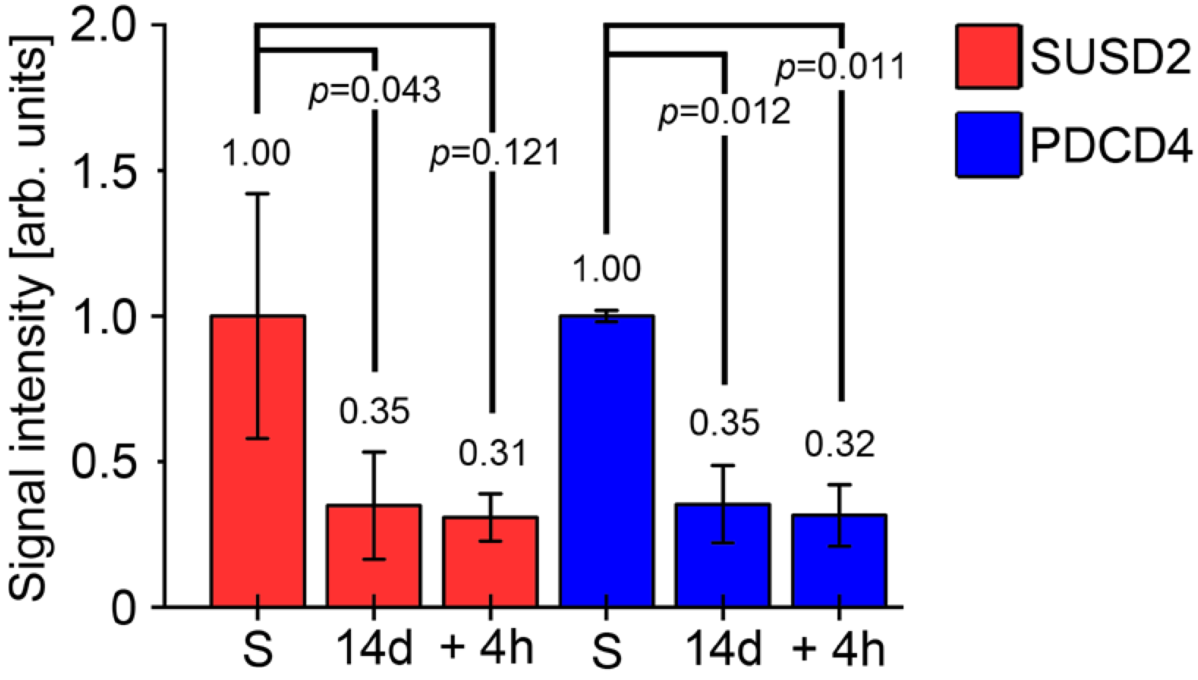

Supplementary Figure S7. Quantitative Western Blot analysis of SUSD2 and PDCD4 in MDA-MB-468 from Figure 5C of the maintext. S: standard cell culture condition. 14d: 14 days 1 % O_2. + 4 h: 14 days 1 % O_2, followed by 10 % O_2 for 4 h. Alpha-tubulin served as a loading control. The *p*-value was calculated using Student's two-sided *t*-test where $p<0.05$ was considered significant. For direct assessment of the fold change, the average values were set to 1 of condition S. Vertical error bars show the standard deviation of three independent measurements.

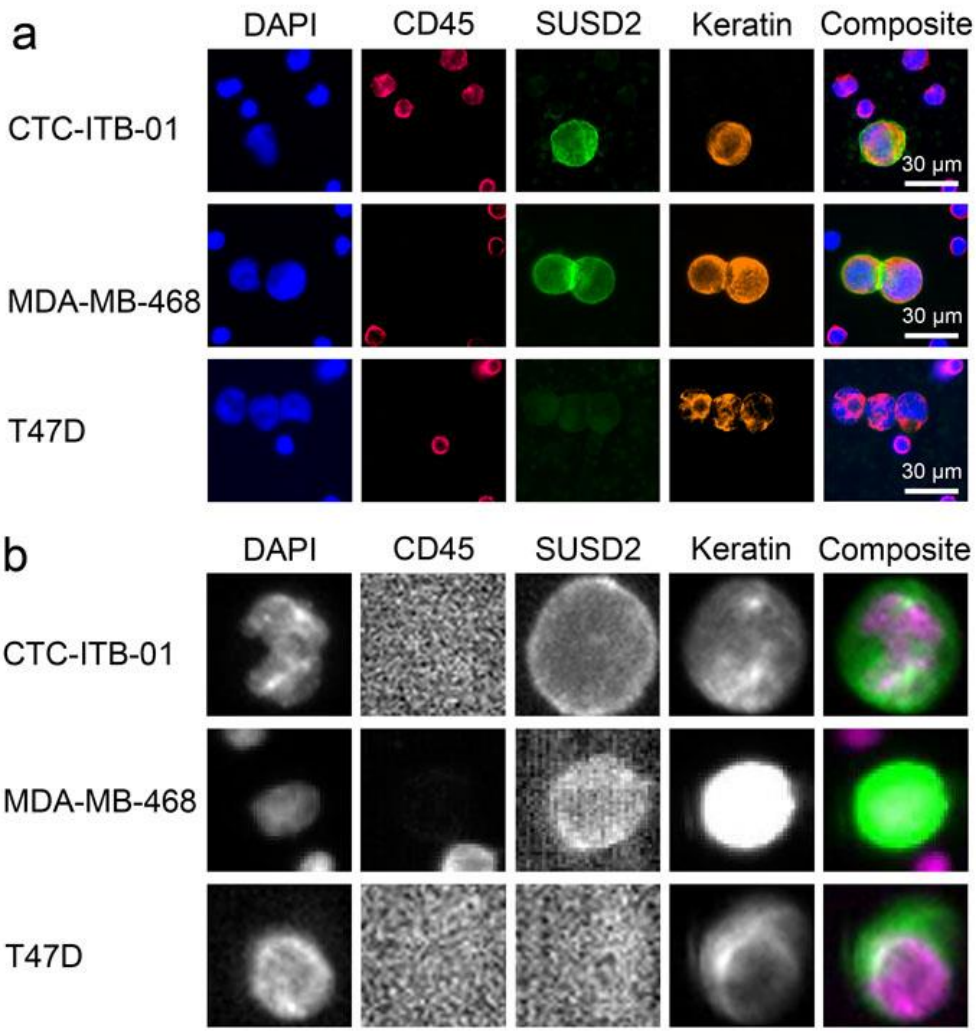

Supplementary Figure S8. Establishment of the immunofluorescent staining for the detection of SUSD2 in CTC by the CellSearch System. CTC-ITB-01 was applied as a strongly SUSD2 positive cell line, MDA-MB-468 as a cell line mainly positive for the 55-kDa variant, and T47D as a SUSD2 weakly positive cell line. A: Establishment of the staining using cell lines spiked into the blood of healthy women. B: Application of the optimized SUSD2 staining protocol to cell lines spiked into the blood of healthy women followed by detection using the CellSearch system. The composite images show overlays of the DAPI, CD45, SUSD2 and the keratin signals. The experiments were performed in biological triplicates (A, B)

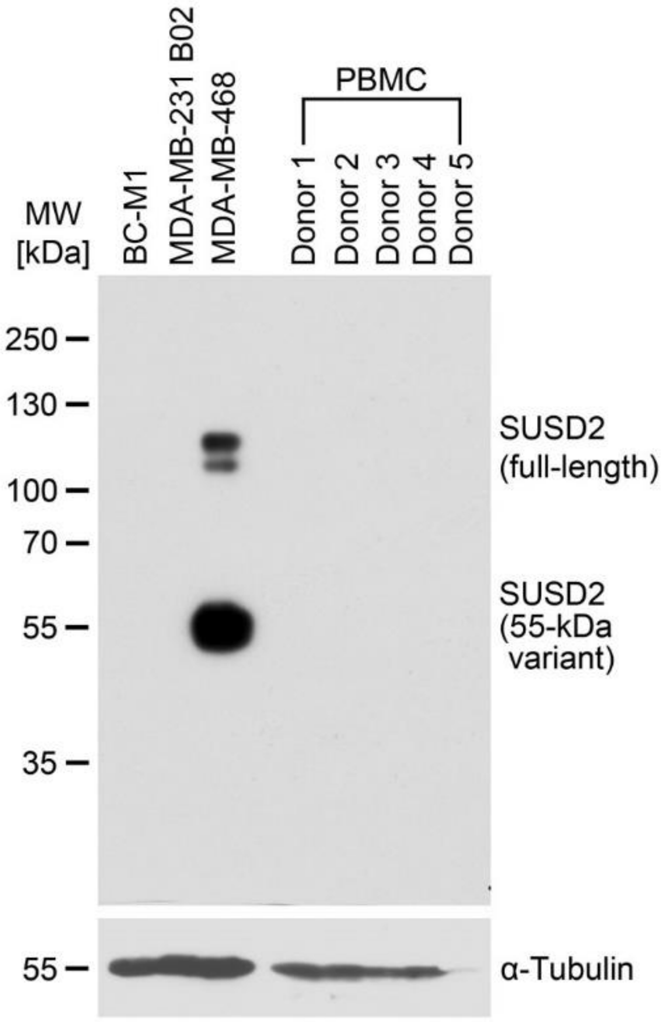

Supplementary Figure S9. Analysis of peripheral blood mononuclear cells of five healthy women (PBMC) for SUSD2 by Western Blot. Alpha-tubulin served as a loading control. Uncropped X-ray film images are shown in supplementary file F1.

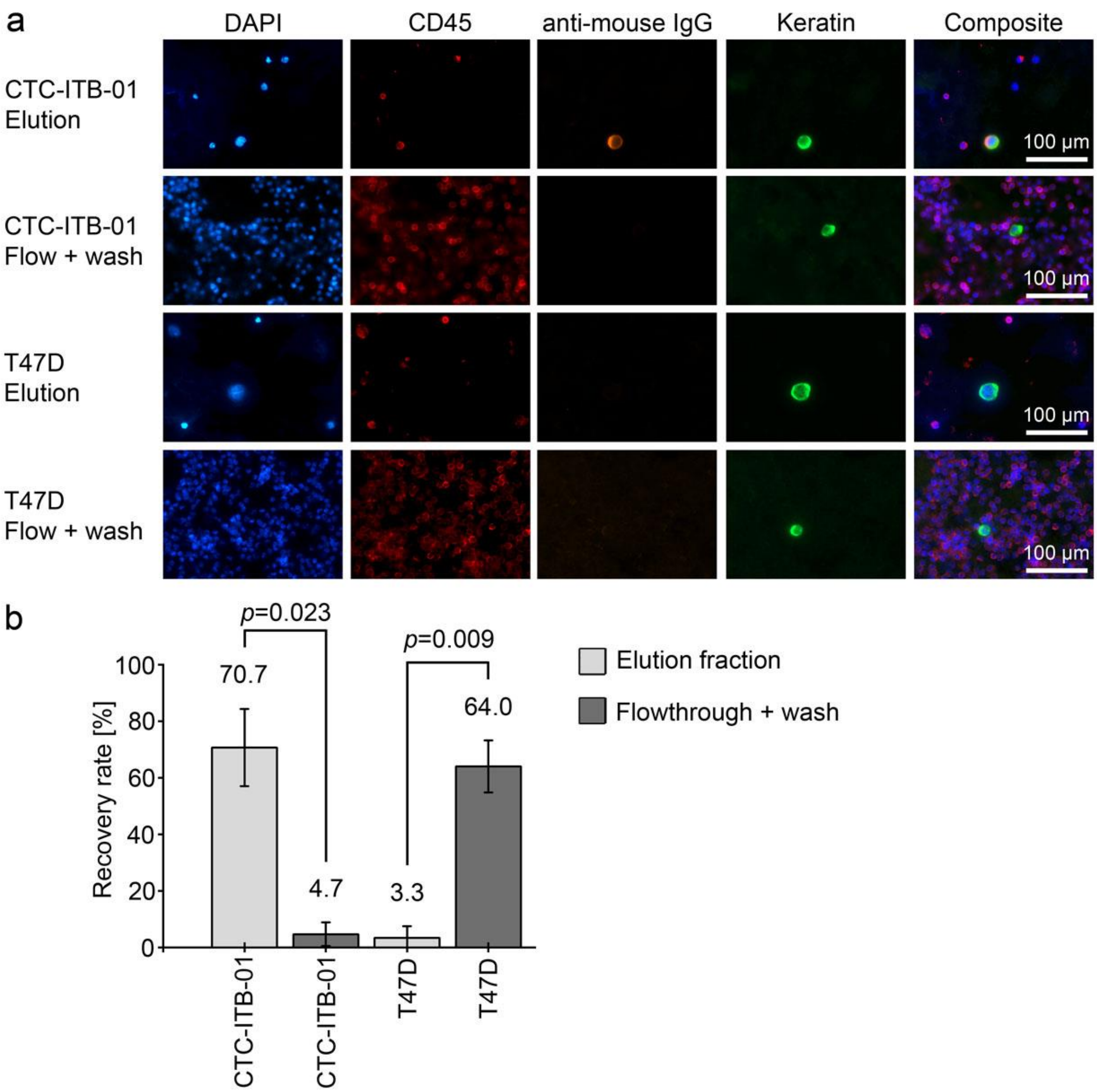

Supplementary Figure S10. Evaluation of the MACS system using SUSD2 MicroBeads. Breast cancer cell lines were spiked into the blood of healthy women and isolated by MACS. The cell line CTC-ITB-01 was used as a SUSD2 positive control, and T47D as a negative control. Slides were stained with anti-mouse IgG to detect the MicroBeads bound tumor cells. Pan-keratin was applied as tumor cell marker, CD45 as leukocyte marker and nuclei were visualized with DAPI. The composite image shows an overlay of the DAPI, CD45, keratin and the anti-mouse IgG. A: Immunocytochemical detection the MACS elution fraction containing the SUSD2 positive cells and the pooled flow through (flow) and wash fraction. In case of T47D, SUSD2 negative tumor cells were occasionally detected in the elution fraction. B: Determination of the recovery rate of the SUSD2 MACS. Fifty cells of the denoted cell lines

were spiked into the blood of healthy women and isolated by the SUSD2 MACS. The values are the average of three independent experiments, and the vertical error bars represent the standard deviation. The *p*-values were calculated using Student's two-sided t-test where $p<0.05$ was considered significant.

Supplementary Tables

Supplementary Table S1. Primary antibodies and dilutions used for Western Blot. mc: monoclonal; pc: polyclonal

Primary antibody	Company	Clone	Clonality	Dilution	Species
anti-E-Cadherin	Epitomics, Burlingame, USA	EP700Y	mc[a]	1:4,000	Rabbit
anti-CXCR4	Abcam, Cambridge, United Kingdom	-	pc[b]	1:4,000	Rabbit
anti-N-Cadherin	Cell Signaling Technology, Danvers, USA	D4R1H	mc	1:10,000	Rabbit
anti-Vimentin	BD Pharmingen, Erembodegem, Belgium	RV202	mc	1:200,000	Mouse
anti-Keratin-8	Progen Biotechnik, Heidelberg, Germany	Ks8.7	mc	1:5,000	Mouse
anti-Keratin-18	Progen Biotechnik, Heidelberg, Germany	Ks18.04	mc	1:10,000	Mouse
Anti-Keratin-19	Antibodies-online, Atlanta, USA	BA-17	mc	1:100,000	Mouse
anti-EGFR	Cell Signaling Technology, Danvers, USA	D38B1	mc	1:1,000	Rabbit
anti-CD44	Cell Signaling Technology, Danvers, USA	-	pc	1:5,000	Rabbit
anti-α-Tubulin	Cell Signaling Technology, Danvers, USA	11H10	mc	1:20,000	Rabbit
anti-Grp78	Cell Signaling Technology, Danvers, USA	C50B12	mc	1:1,000	Rabbit
anti-pAKT (S473)	Cell Signaling Technology, Danvers, USA	D9E	mc	1:1,000	Rabbit
anti-AKT	Cell Signaling Technology, Danvers, USA	-	pc	1:1,000	Rabbit
anti-HIF1α	Cell Signaling Technology, Danvers, USA	D2U3T	mc	1:1,000	Rabbit
anti-pERK (T202/Y204)	Cell Signaling Technology, Danvers, USA	-	pc	1:4,000	Rabbit
anti-ERK	Cell Signaling Technology, Danvers, USA	-	pc	1:1,000	Rabbit
anti-ErbB2	Novocastra/Leica Biosystems, Nussloch, Germany	CB11	mc	1:10,000	Mouse
anti-EpCAM	Cell Signaling Technology, Danvers, USA	VU-1D9	mc	1:1,000	Mouse
anti-pSTAT3 (Y705)	Cell Signaling Technology, Danvers, USA	D3A7	mc	1:1,000	Rabbit
anti-STAT3	Cell Signaling Technology, Danvers, USA	79D7	mc	1:2,000	Rabbit
anti-ERα	Abcam, Cambridge, United Kingdom	SP1	mc	1:1,000	Rabbit
anti-PD-L1	Cell Signaling Technology, Danvers, USA	E1L3N	mc	1:2,000	Rabbit
anti-Integrin beta 5	Cell Signaling Technology, Danvers, USA	D24A5	mc	1:1,000	Rabbit
anti-Gelsolin	DAKO, Glostrup, Denmark	-	pc	1:10,000	Rabbit
anti-SUSD2	Abcam, Cambridge, United Kingdom	EPR8913(2)	mc	1:1,000	Rabbit
anti-ENPP1	Abcam, Cambridge, United Kingdom	EPR22262-22	mc	1:5,000	Rabbit
anti-PR	Abcam, Cambridge, United Kingdom	-	pc	1:1,000	Rabbit
anti-Plexin-A1	Cell Signaling Technology, Danvers, USA	-	pc	1:1000	Rabbit
anti-BCL-2	Abcam, Cambridge, United Kingdom	EPR17509	mc	1:5000	Rabbit
anti-PDCD4	Cell Signaling Technology, Danvers, USA	D29C6	mc	1:5000	Rabbit
anti-Raptor	Cell Signaling Technology, Danvers, USA	24C12	mc	1:1000	Rabbit
anti-Reticulocalbin-1	Abcam, Cambridge, United Kingdom	EPR17163-117	mc	1:1000	Rabbit
anti-p70 S6 kinase	Cell Signaling Technology, Danvers, USA	-	pc	1:10,000	Rabbit
anti-phospho-p70 S6 kinase (Thr389)	Cell Signaling Technology, Danvers, USA	1A5	mc	1:1000	Mouse
anti-Galectin-1	Pierce Biotech., Rockford, USA	-	pc	1:3,000	Rabbit

Supplementary Table S4. Western Blot analysis details for the SUSD2 overexpressing MCF-7 cell lines (clones OE1, OE2). *p*-value: Student's two-sided *t*-test, with $p<0.05$ was considered significant.

Analyzed protein (Uniprot acc. no.)	Comparison control versus	Normalized to	Ratio ± standard deviation	*p*-value	Associated pathway
SUSD2 (Q9UGT4)	OE1, OE2	-	-	-	Input parameter
PDCD4 (Q53EL6)	OE1	α-tubulin	0.914 ± 0.318	0.663	mTOR
PDCD4 (Q53EL6)	OE2	α-tubulin	0.976 ± 0.291	0.912	mTOR
Estrogen Receptor α (P03372)	OE1	α-tubulin	2.884 ± 0.266	0.016	Steroid receptor superfamily
Estrogen Receptor α (P03372)	OE2	α-tubulin	3.194 ± 0.370	0.015	Steroid receptor superfamily
Reticulocalbin-1 (Q15293)	OE1	α-tubulin	0.147 ± 0.089	0.044	Endoplasmic reticulum stress
Reticulocalbin-1 (Q15293)	OE2	α-tubulin	0.128 ± 0.113	0.048	Endoplasmic reticulum stress
p70 S6 Kinase (P23443)	OE1	α-tubulin	0.966 ± 0.087	0.425	mTOR
p70 S6 Kinase (P23443)	OE2	α-tubulin	1.154 ± 0.180	0.139	mTOR
Phospho-p70 S6 Kinase, Thr389 (P23443)	OE1	p70 S6 Kinase	0.145 ± 0.209	0.030	mTOR
Phospho-p70 S6 Kinase, Thr389 (P23443)	OE2	p70 S6 Kinase	1.088 ± 0.304	0.824	mTOR
Raptor (Q8N122)	OE1	α-tubulin	1.080 ± 0.121	0.587	mTOR
Raptor (Q8N122)	OE2	α-tubulin	0.983 ± 0.205	0.935	mTOR
GRP78 (P11021)	OE1	α-tubulin	2.523 ± 0.286	0.037	Endoplasmic reticulum stress
GRP78 (P11021)	OE2	α-tubulin	3.316 ± 0.672	0.044	Endoplasmic reticulum stress
ENPP1 (P22413)	OE1	α-tubulin	1.075 ± 0.268	0.418	Stimulator of interferon genes (STING); innate immune signalling
ENPP1 (P22413)	OE2	α-tubulin	0.744 ± 0.122	0.335	Stimulator of interferon genes (STING); innate immune signalling
ErbB-2 (P04626)	OE1	α-tubulin	1.760 ± 0.172	0.173	ErbB family
ErbB-2 (P04626)	OE2	α-tubulin	1.998 ± 0.508	0.234	ErbB family
AKT (P31749, P31751, Q9Y243)	OE1	α-tubulin	1.084 ± 0.193	0.597	Link mTOR-ErbB-2
AKT (P31749, P31751, Q9Y243)	OE2	α-tubulin	1.271 ± 0.395	0.324	Link mTOR-ErbB-2
Phospho-AKT, Ser473 (P31749, P31751, Q9Y243)	OE1	AKT	0.569 ± 0.124	0.302	Link mTOR-ErbB-2
Phospho-AKT, Ser473 (P31749, P31751, Q9Y243)	OE2	AKT	0.424 ± 0.284	0.370	Link mTOR-ErbB-2

Supplementary Table S5. Clinicopathological parameters, CTC and SUSD2 detection rates in blood samples from breast cancer patients by CellSearch.

Patient ID	Time interval	CTC count intact	CTC count apoptotic	ER	PR	HER2	Sushi 1Plus	Sushi 2Plus	Sushi 3Plus
1		17	8	pos.	pos.	neg.	1	0	
2		5	7	12/12	2/12	0	0	0	
3		2	3	0%	0%	1+	2	1	
4		19	10	12/12	6/12	0	0	0	
5		40	26	12/12	2/12	1+	0	0	
6		2	6	12/12	12/12	neg.	0	0	
7		1	1	0%	0%	pos.	0	0	
8		0	10	85%	90%	0	0	0	
9		0	1	60%	70%	neg.	0	0	
10		1	4	0%	0%	1+	0	0	
11		0	3	100%	100%	1+	0	0	
12		18	48	100%	10%	0	3 very weak	0	
13		0	4	7/8	7/8	1+	0	0	
14		2	0	0%	0%	neg.	0	0	
15		6	13	11/12	2/12	1+	4	0	
16		3	8	0%	0%	0	0	0	
17		10	36	neg.	neg.	neg.	5	0	
18		98	396	pos.	pos.	neg.	0	0	
19		3	10	100%	5%	0	1 (weak)	0	
19	35 days later	2	5	100%	5%	0	0	0	
20		10	5	8/12	9/12	neg.	8	1	
20	28 days later	6	11	8/8	6/8	1+	4	1	
21		212	351	80%	20%	1+	0	0	
21	112 days later	304	630	80%	20%	1+	36	0	1
22		24	142	8/12	9/12	neg.	3	0	
22	14 days later	35	82	8/12	9/12	neg.	5	0	
23		11	3	100%	80%	3+	0	0	
23	14 days later	9	10	100%	80%	3+	0	0	
23	14 days later	2	10	100%	80%	3+	0	0	

Uncropped Western Blot x-ray film images

For Figure 1:

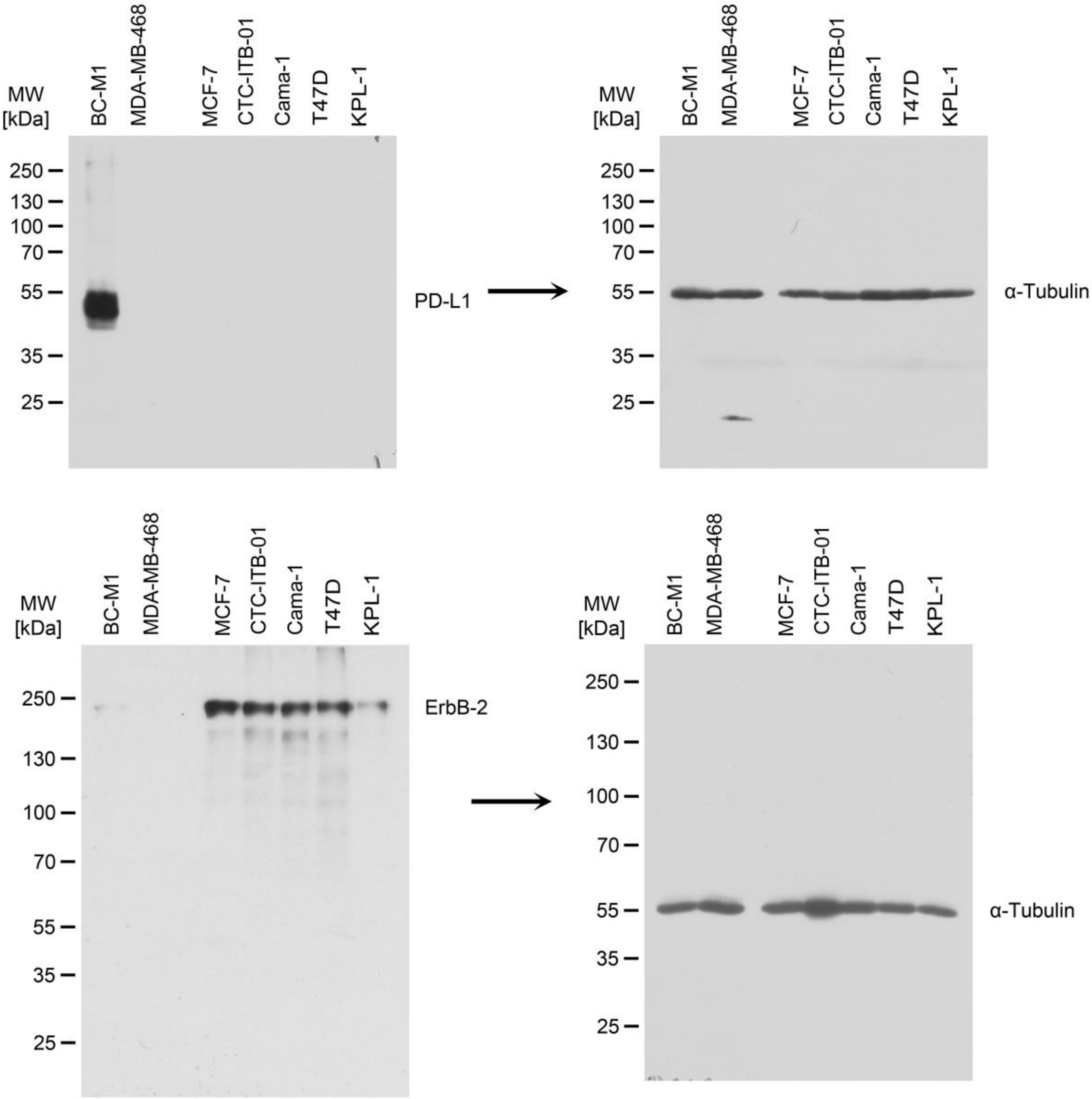

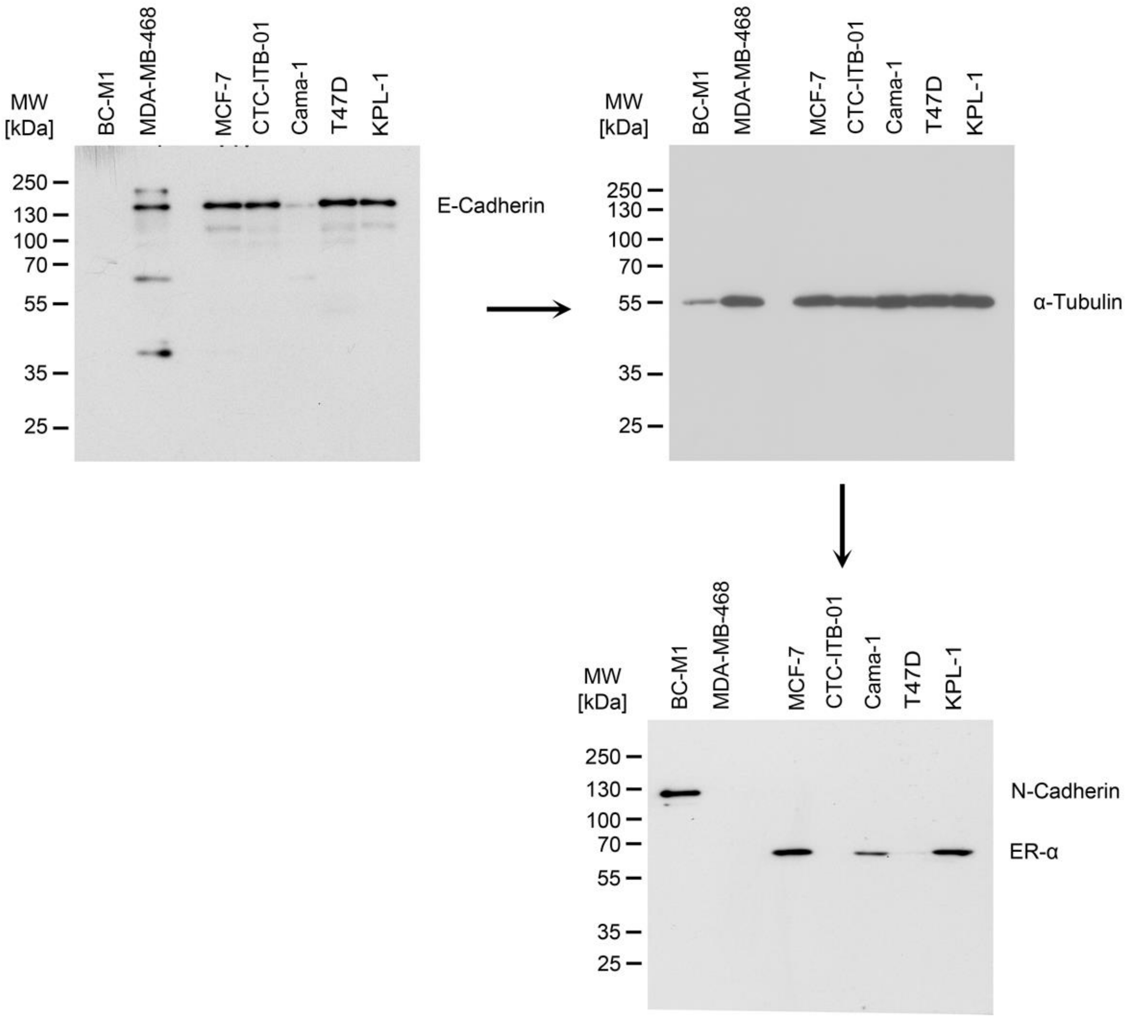
MW [kDa]
BC-M1
MDA-MB-468
MCF-7
CTC-ITB-01
Cama-1
T47D
KPL-1
250
130
100
70
55
35
25
E-Cadherin
α-Tubulin
N-Cadherin
ER-α

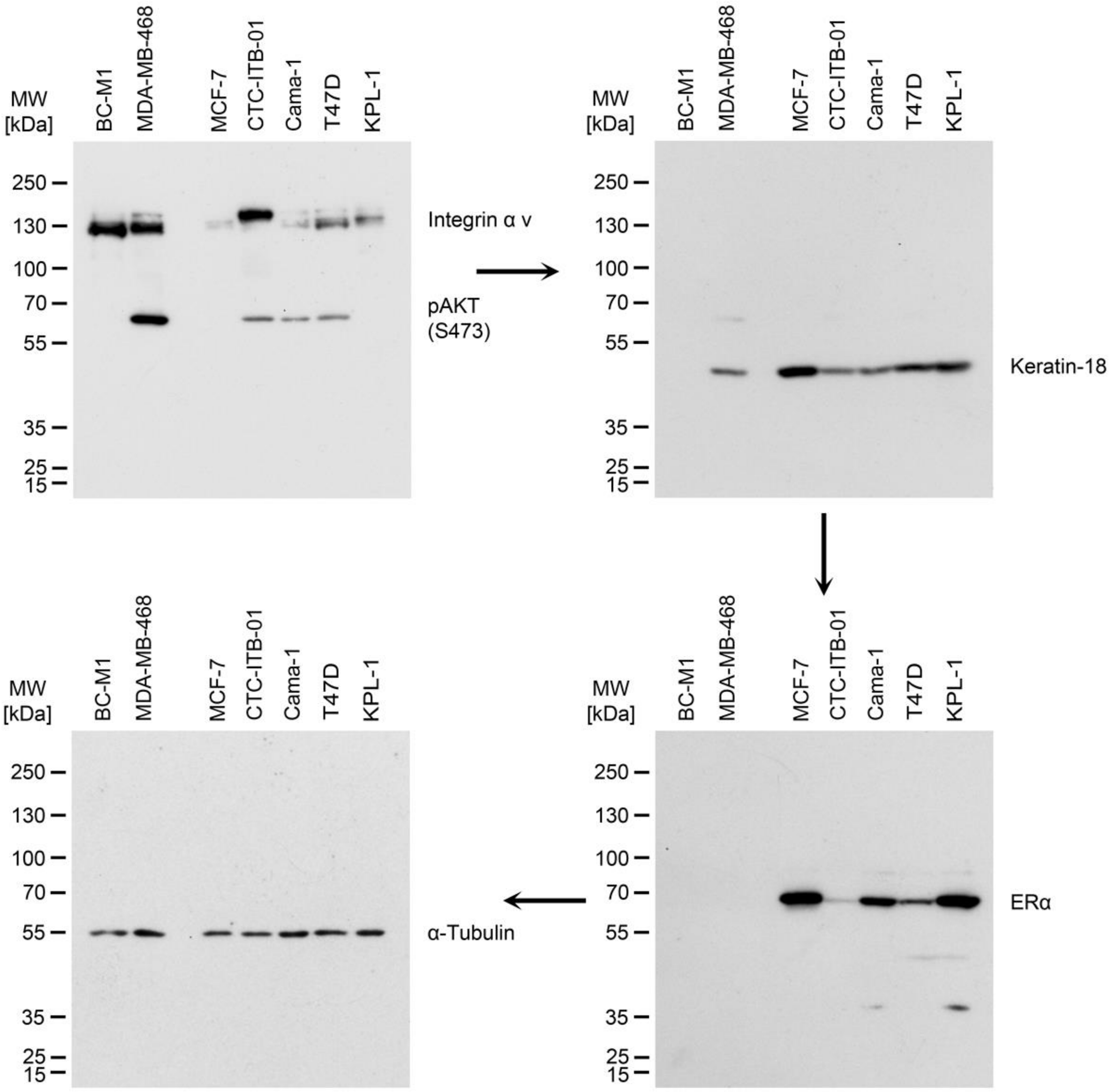

MW [kDa]
BC-M1
MDA-MB-468
MCF-7
CTC-ITB-01
Cama-1
T47D
KPL-1
250
130
100
70
55
35
25
15
Integrin α v
pAKT (S473)
Keratin-18
ERα
α-Tubulin

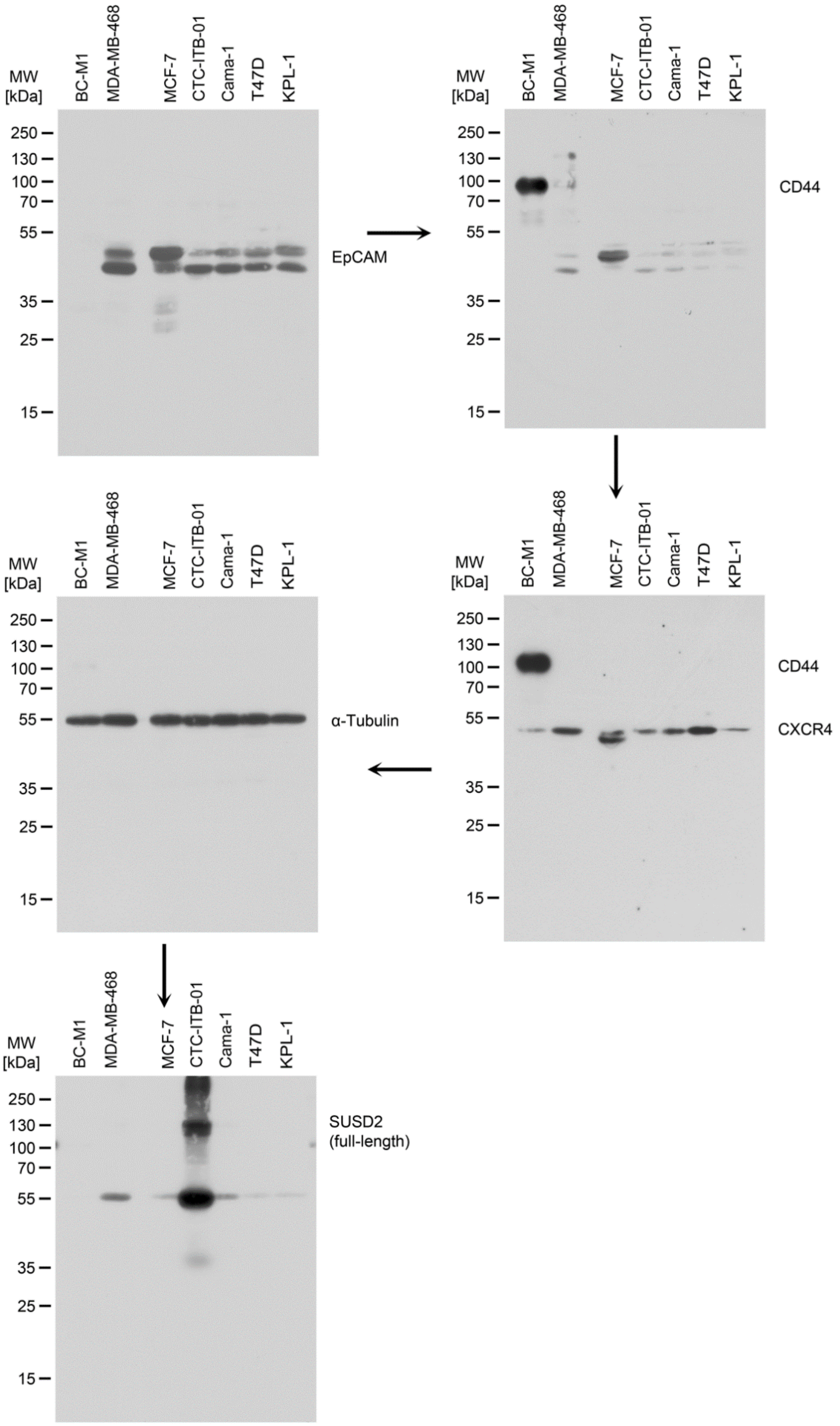
MW [kDa]
BC-M1
MDA-MB-468
MCF-7
CTC-ITB-01
Cama-1
T47D
KPL-1
250
130
100
70
55
35
25
15
EpCAM
CD44
CXCR4
α-Tubulin
SUSD2 (full-length)

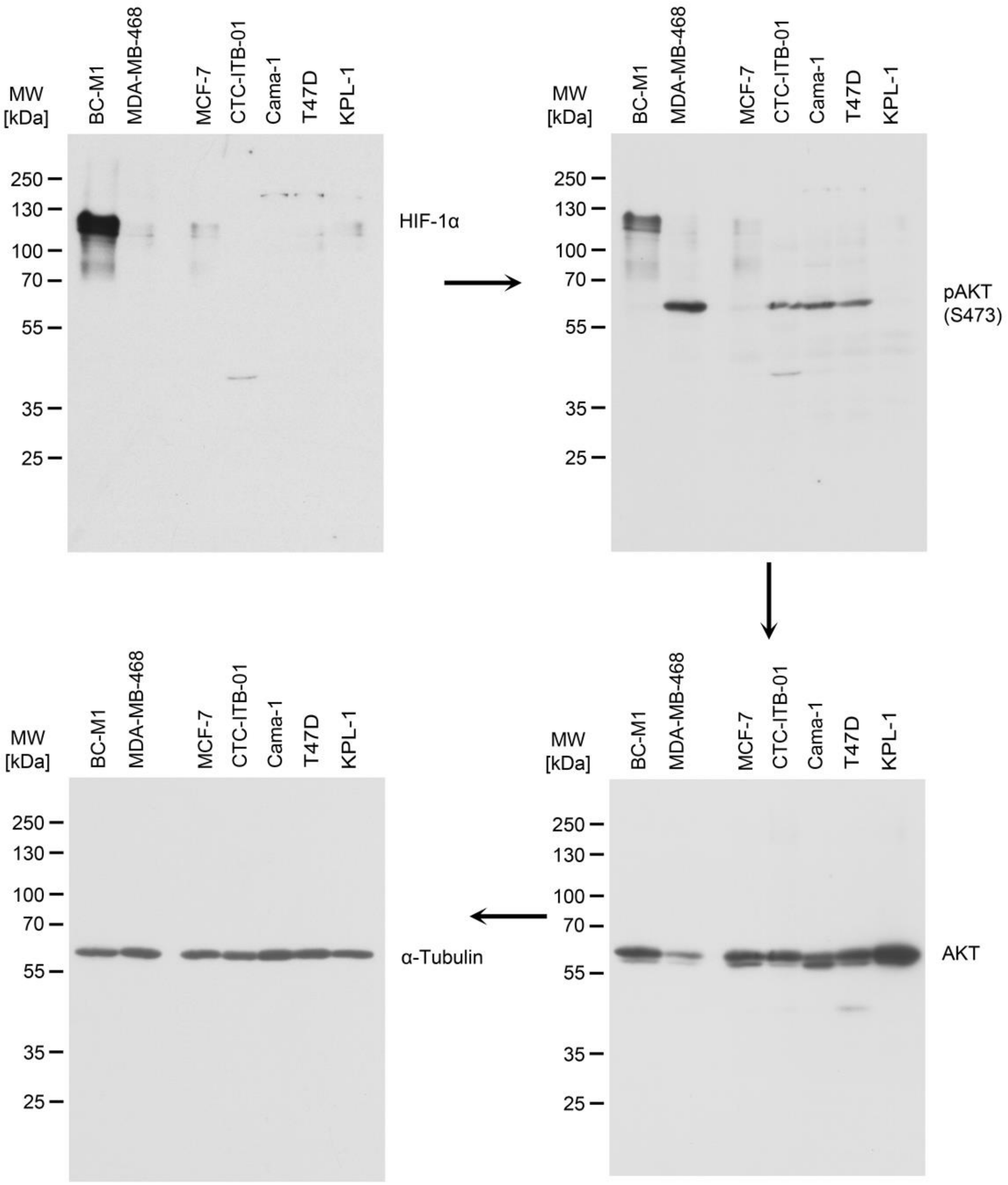
MW [kDa]
BC-M1
MDA-MB-468
MCF-7
CTC-ITB-01
Cama-1
T47D
KPL-1
250
130
100
70
55
35
25
HIF-1α
MW [kDa]
BC-M1
MDA-MB-468
MCF-7
CTC-ITB-01
Cama-1
T47D
KPL-1
250
130
100
70
55
35
25
pAKT (S473)
MW [kDa]
BC-M1
MDA-MB-468
MCF-7
CTC-ITB-01
Cama-1
T47D
KPL-1
250
130
100
70
55
35
25
α-Tubulin
MW [kDa]
BC-M1
MDA-MB-468
MCF-7
CTC-ITB-01
Cama-1
T47D
KPL-1
250
130
100
70
55
35
25
AKT

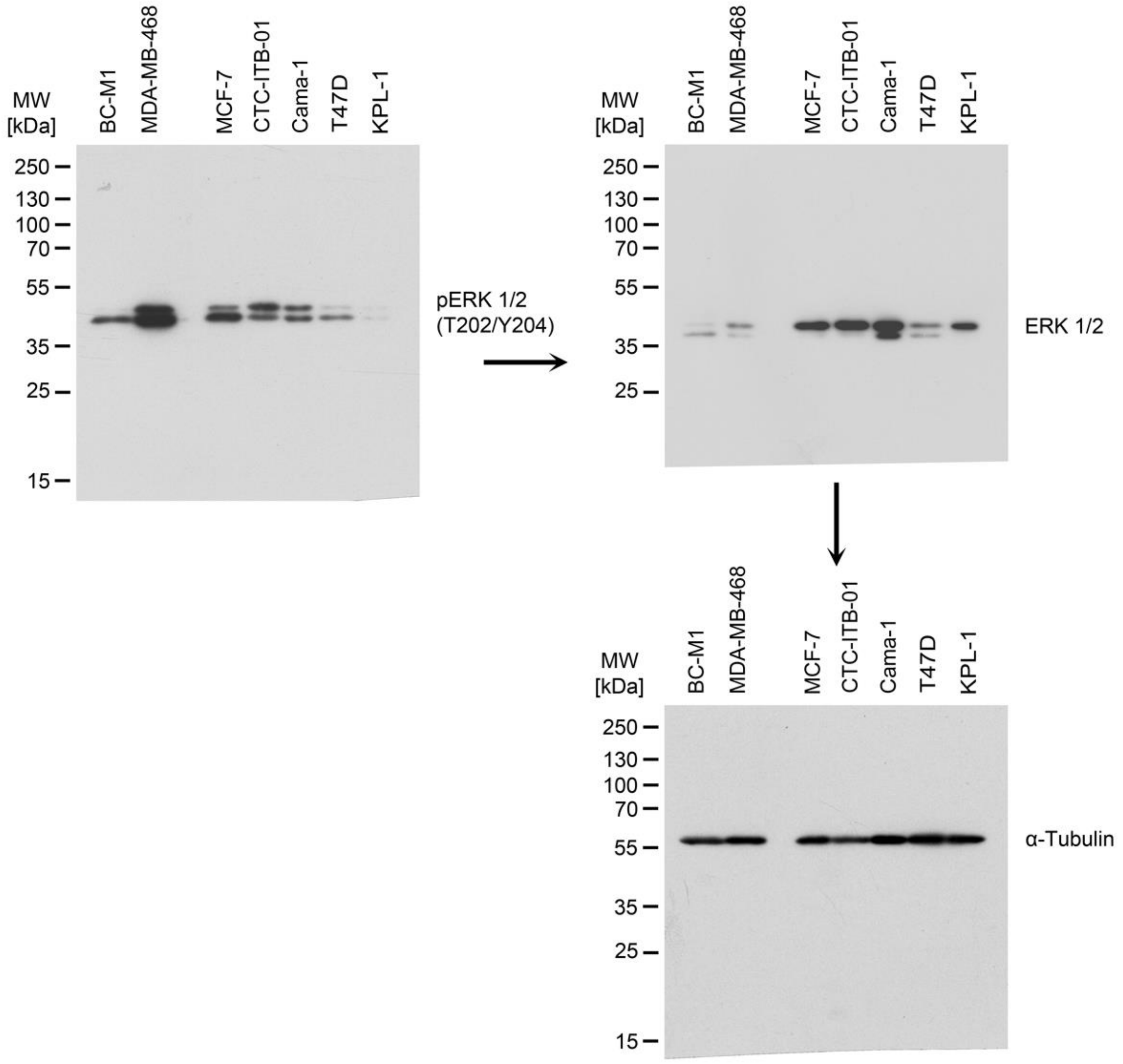
MW [kDa]
BC-M1
MDA-MB-468
MCF-7
CTC-ITB-01
Cama-1
T47D
KPL-1
250
130
100
70
55
35
25
15
pERK 1/2 (T202/Y204)
ERK 1/2
α-Tubulin

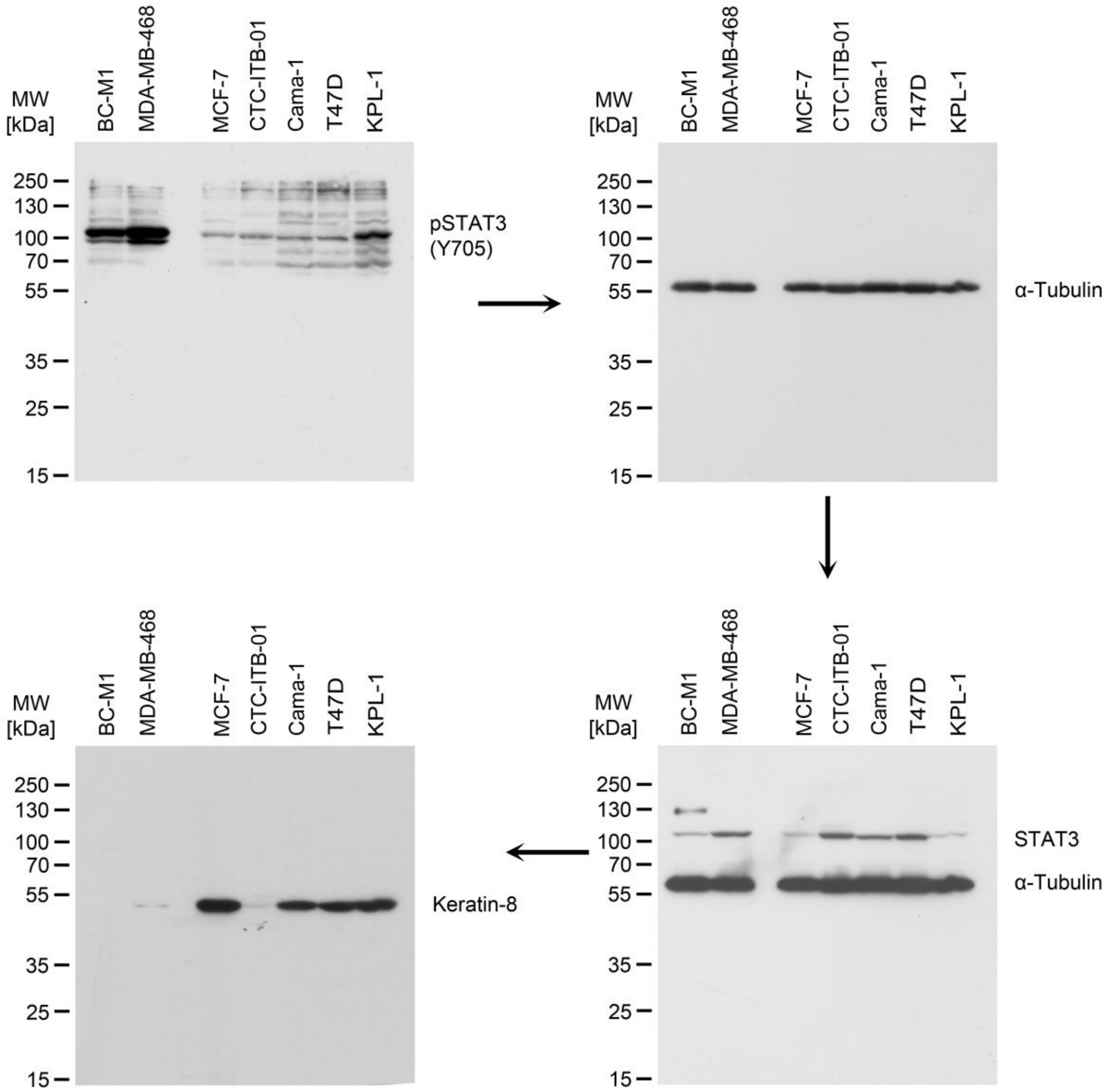
MW
[kDa]
BC-M1
MDA-MB-468
MCF-7
CTC-ITB-01
Cama-1
T47D
KPL-1
250
130
100
70
55
35
25
15
pSTAT3
(Y705)
α-Tubulin
STAT3
α-Tubulin
Keratin-8

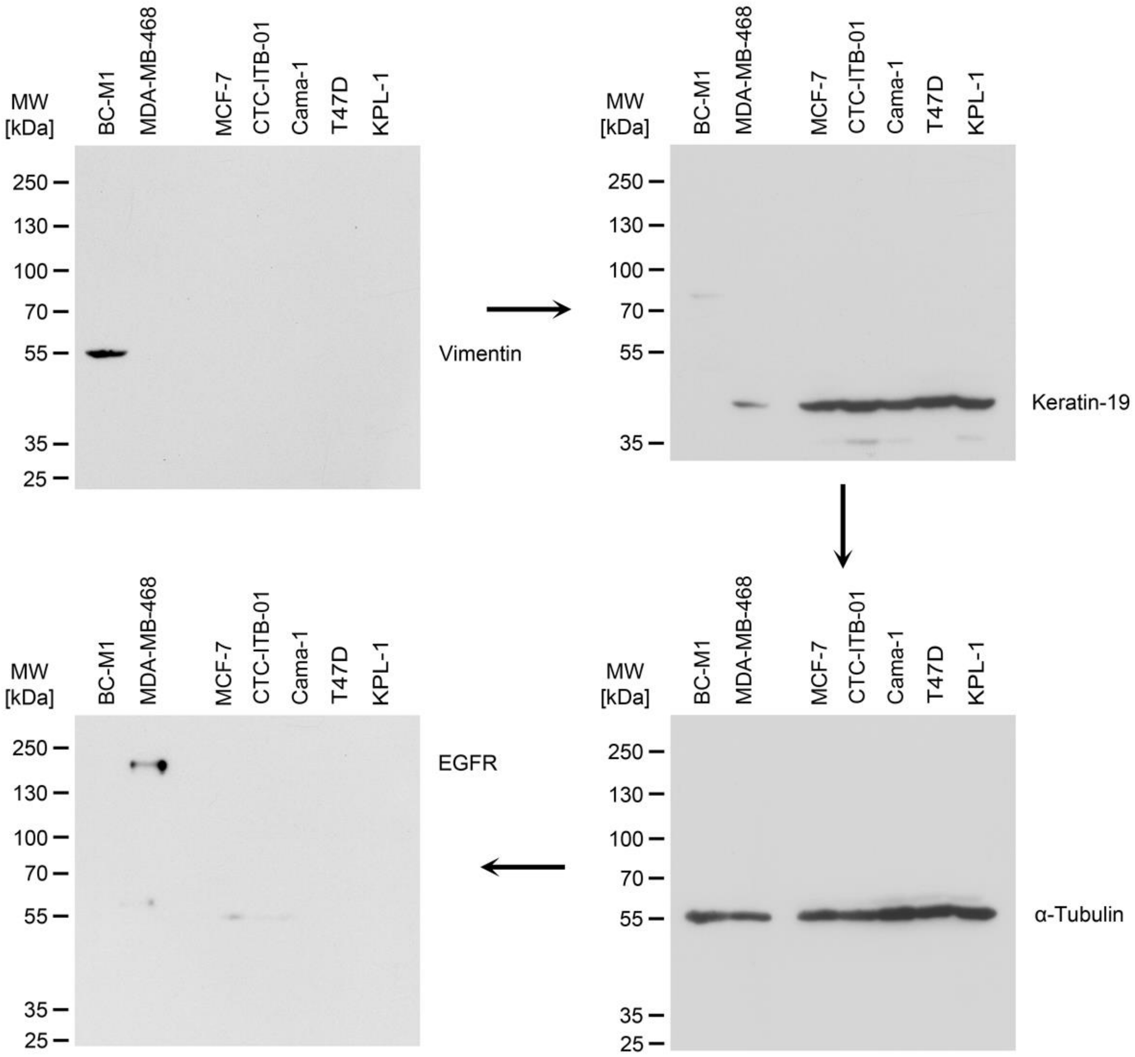
MW [kDa]
BC-M1
MDA-MB-468
MCF-7
CTC-ITB-01
Cama-1
T47D
KPL-1
250
130
100
70
55
35
25
Vimentin
Keratin-19
α-Tubulin
EGFR

For Figure 2 and 3:

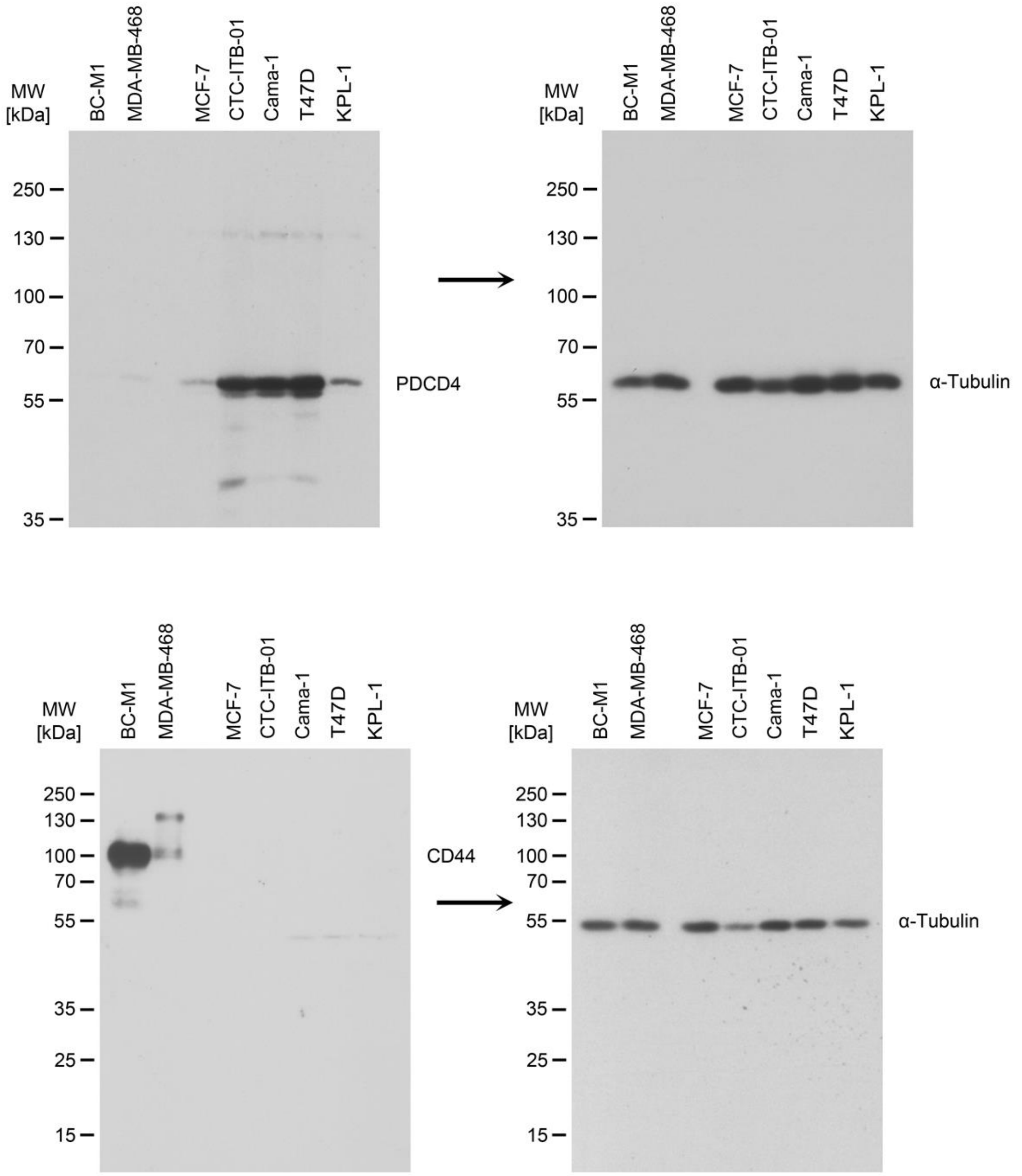

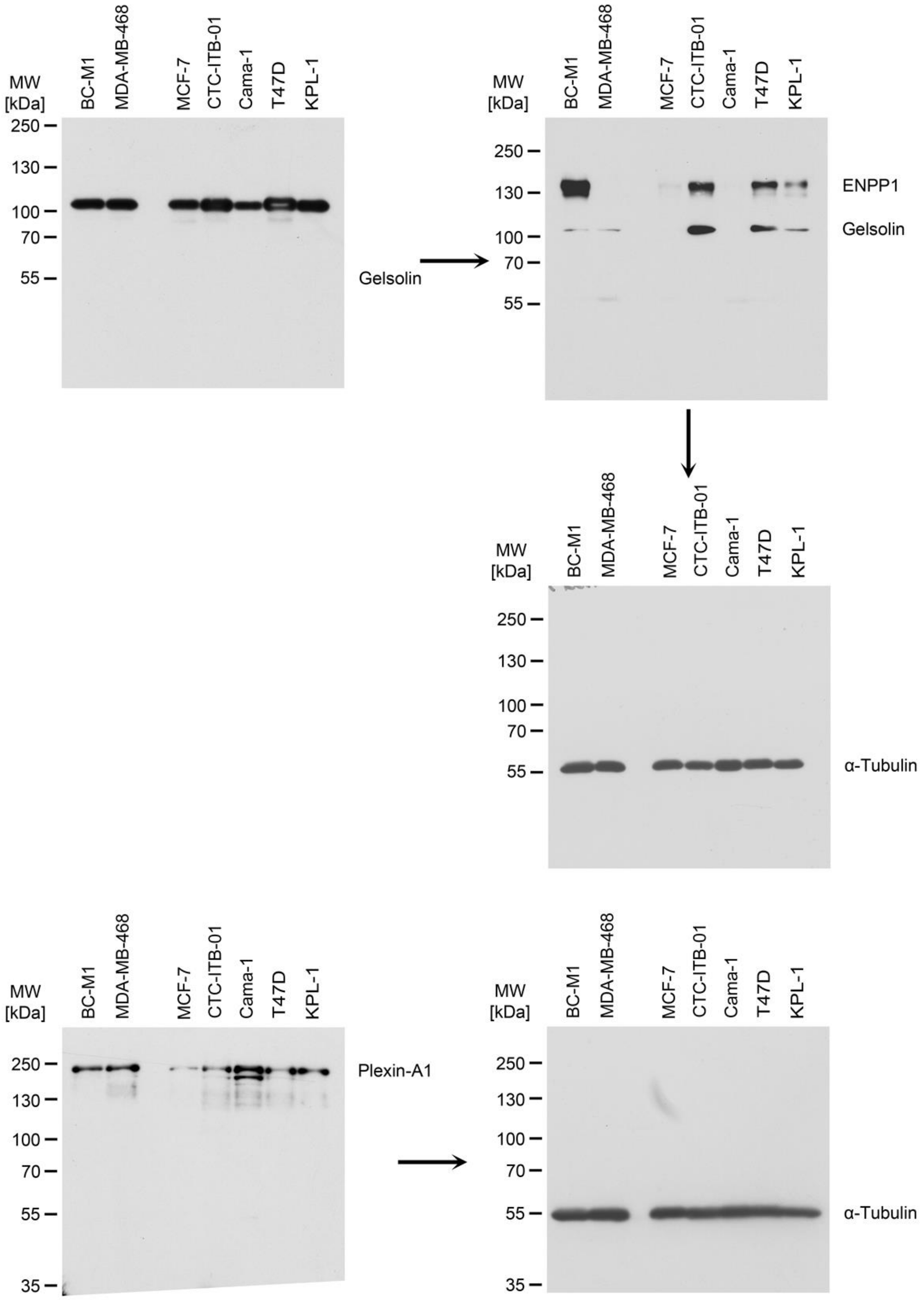
MW [kDa]
BC-M1
MDA-MB-468
MCF-7
CTC-ITB-01
Cama-1
T47D
KPL-1
250
130
100
70
55
35
Gelsolin
ENPP1
Gelsolin
α-Tubulin
Plexin-A1
α-Tubulin

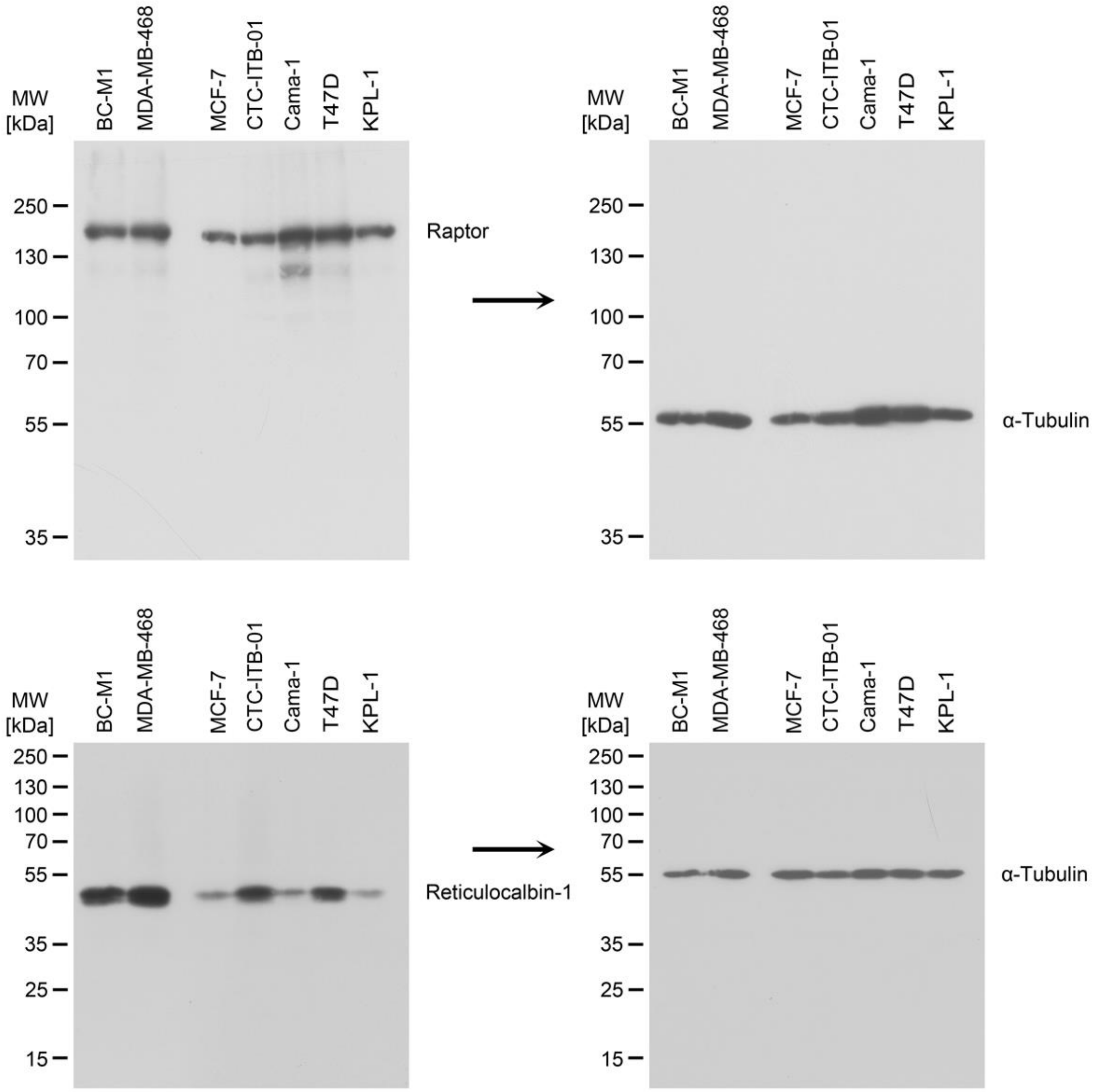
MW
[kDa]
BC-M1
MDA-MB-468
MCF-7
CTC-ITB-01
Cama-1
T47D
KPL-1
250
130
100
70
55
35
Raptor
α-Tubulin
25
15
Reticulocalbin-1

For Figure 4C Maintext and Figure S3

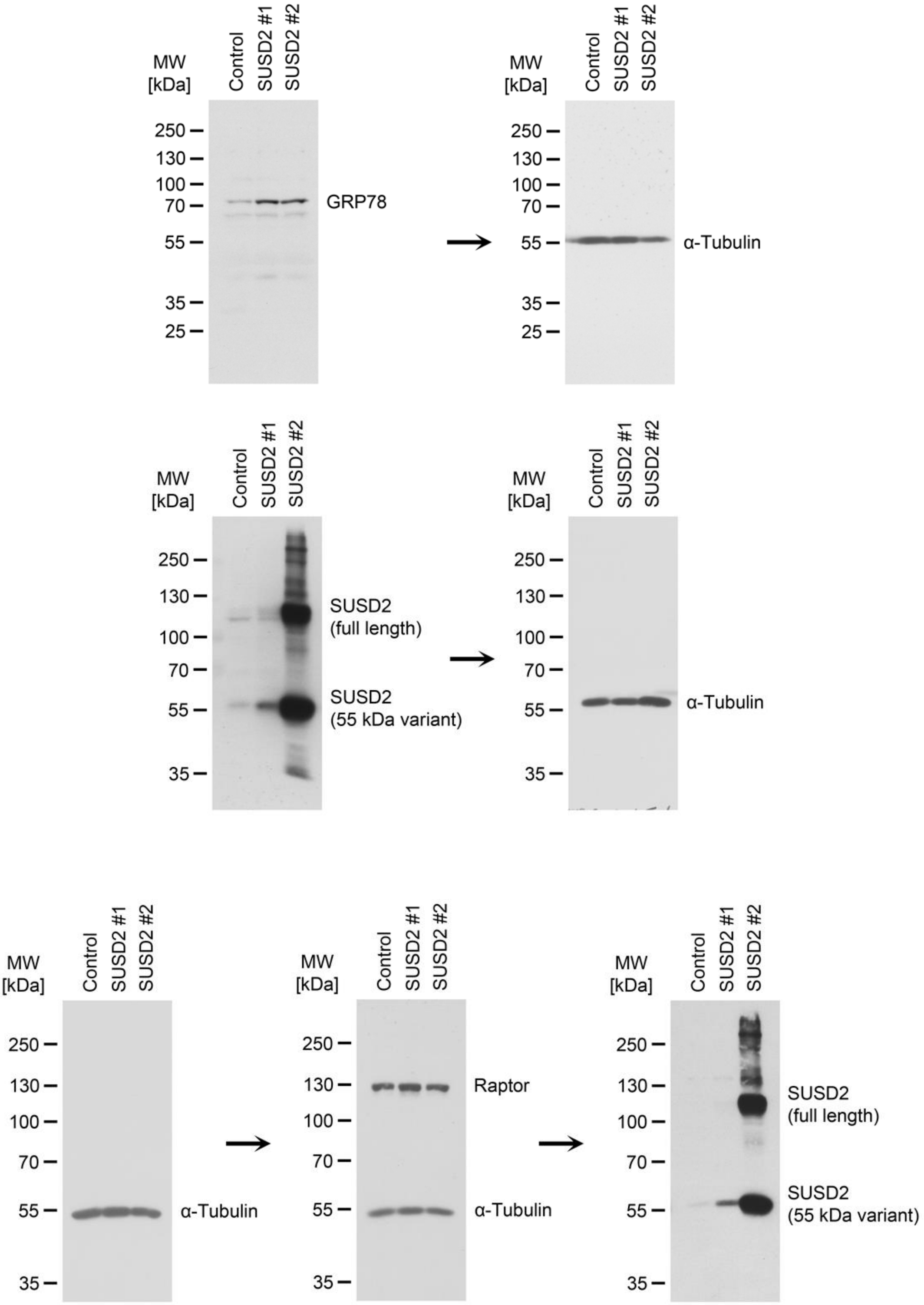

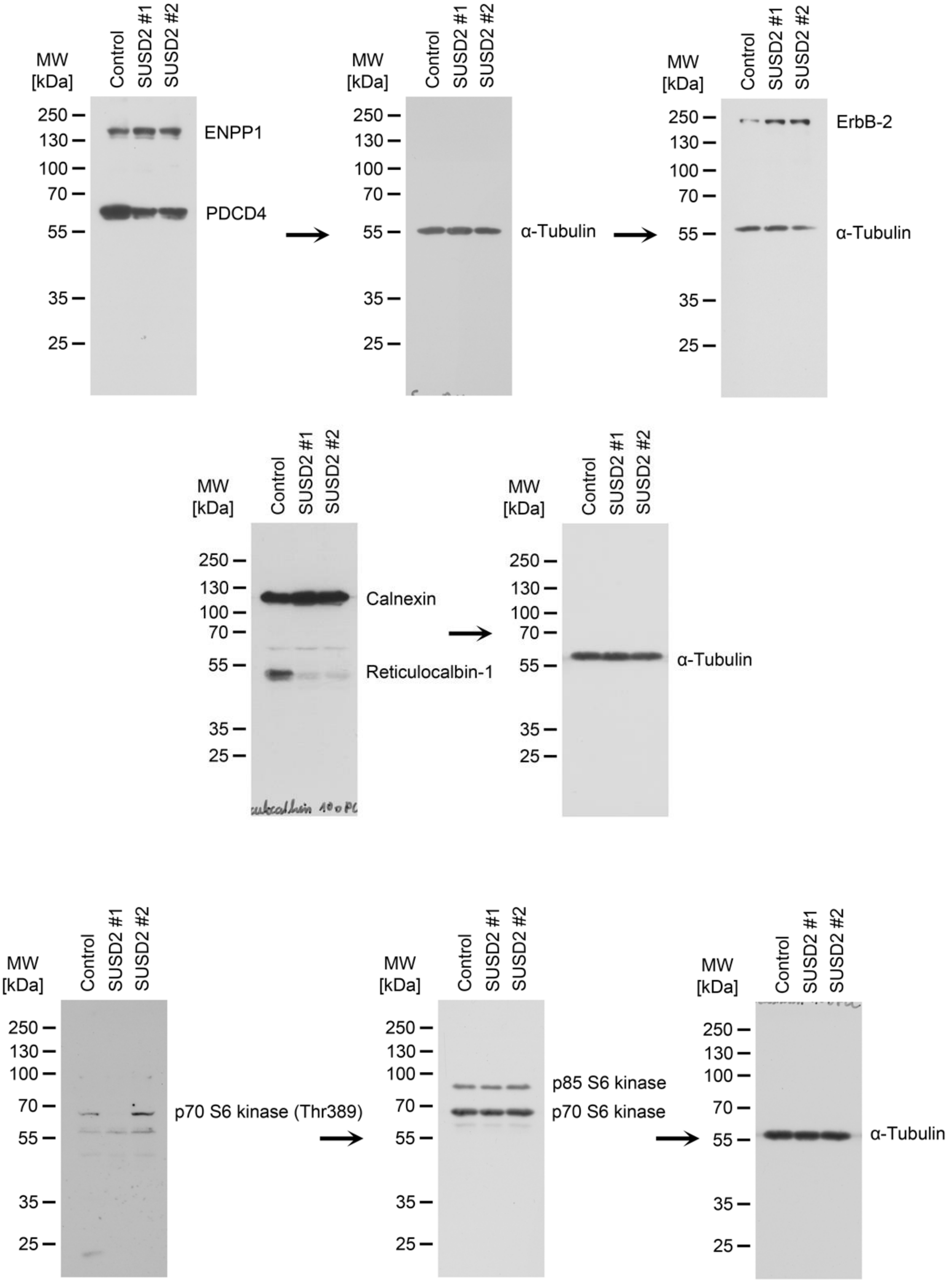
MW [kDa]
Control
SUSD2 #1
SUSD2 #2
250
130
100
70
55
35
25
ENPP1
PDCD4
α-Tubulin
ErbB-2
α-Tubulin
Calnexin
Reticulocalbin-1
α-Tubulin
p70 S6 kinase (Thr389)
p85 S6 kinase
p70 S6 kinase
α-Tubulin

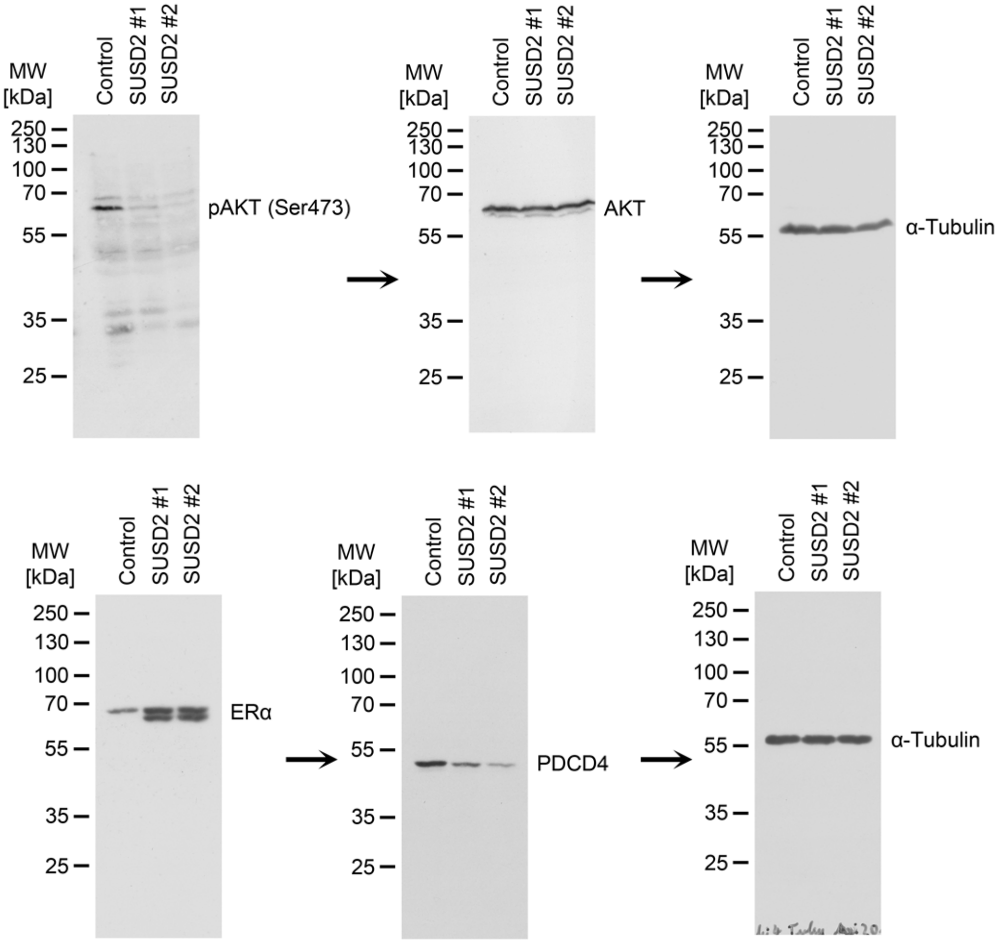
MW [kDa]
Control
SUSD2 #1
SUSD2 #2
250
130
100
70
55
35
25
pAKT (Ser473)
AKT
α-Tubulin
ERα
PDCD4
α-Tubulin

For Figure 4E:

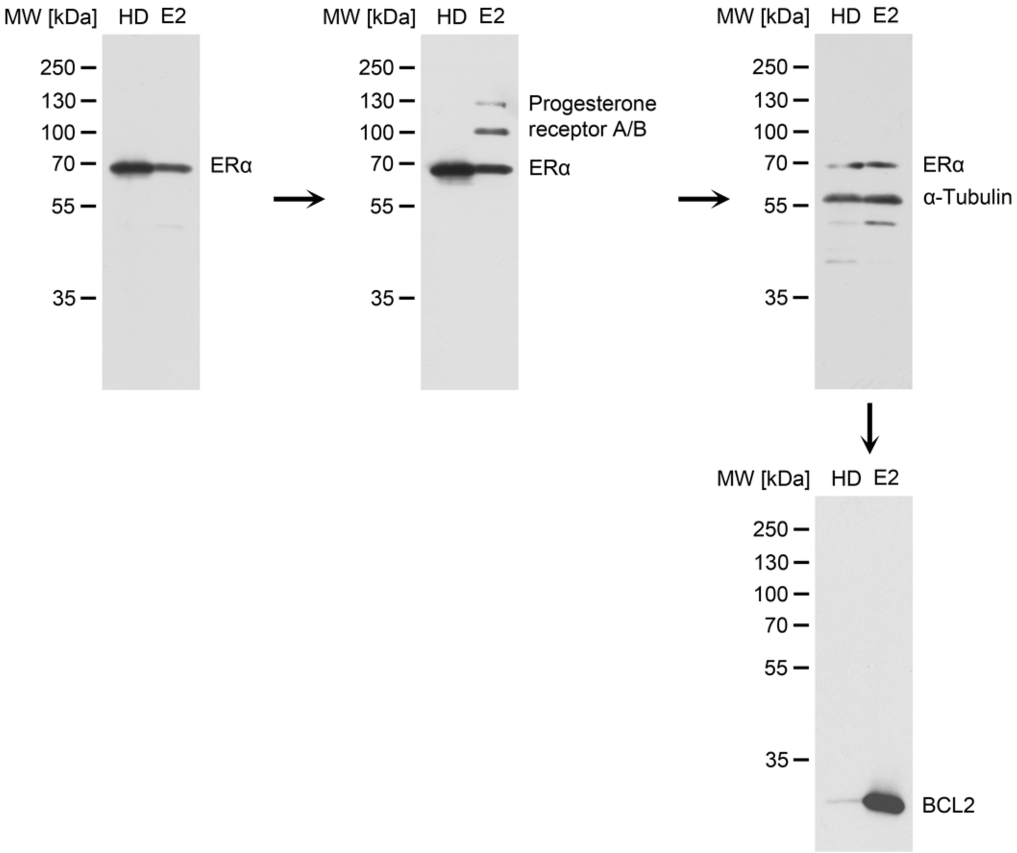

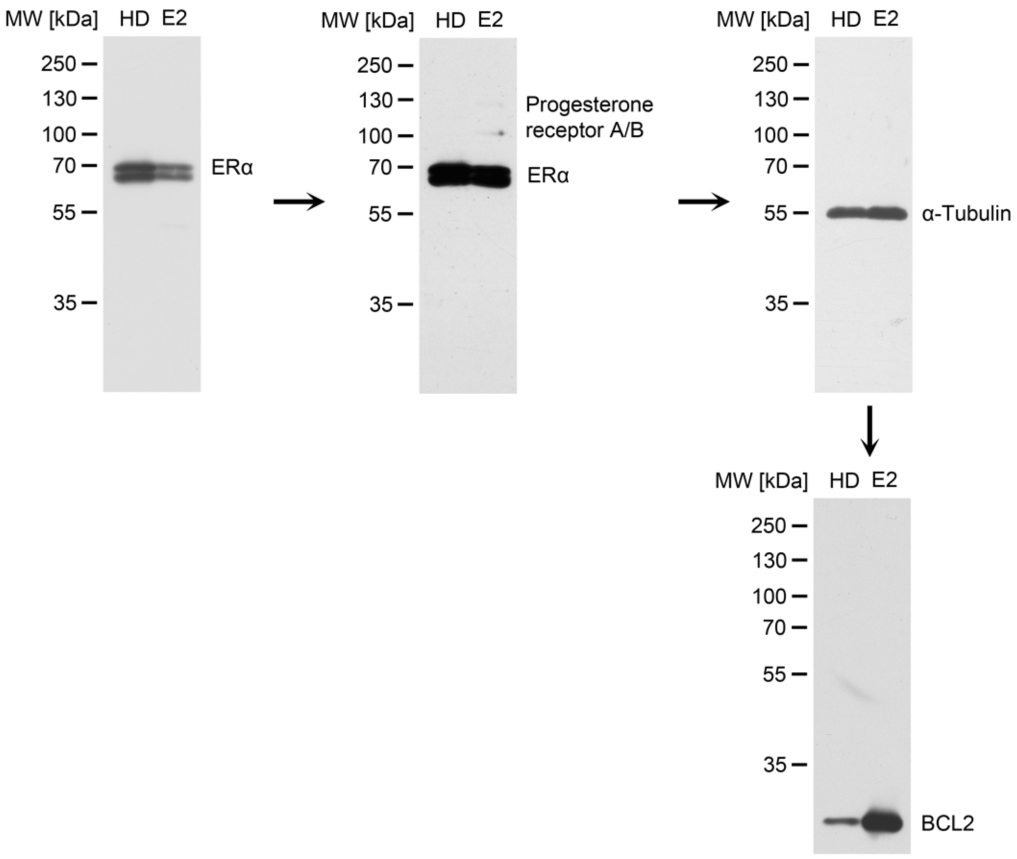

MW [kDa]
HD
E2
250
130
100
70
55
35
ERα
Progesterone receptor A/B
ERα
α-Tubulin
BCL2

For Figure 5A:

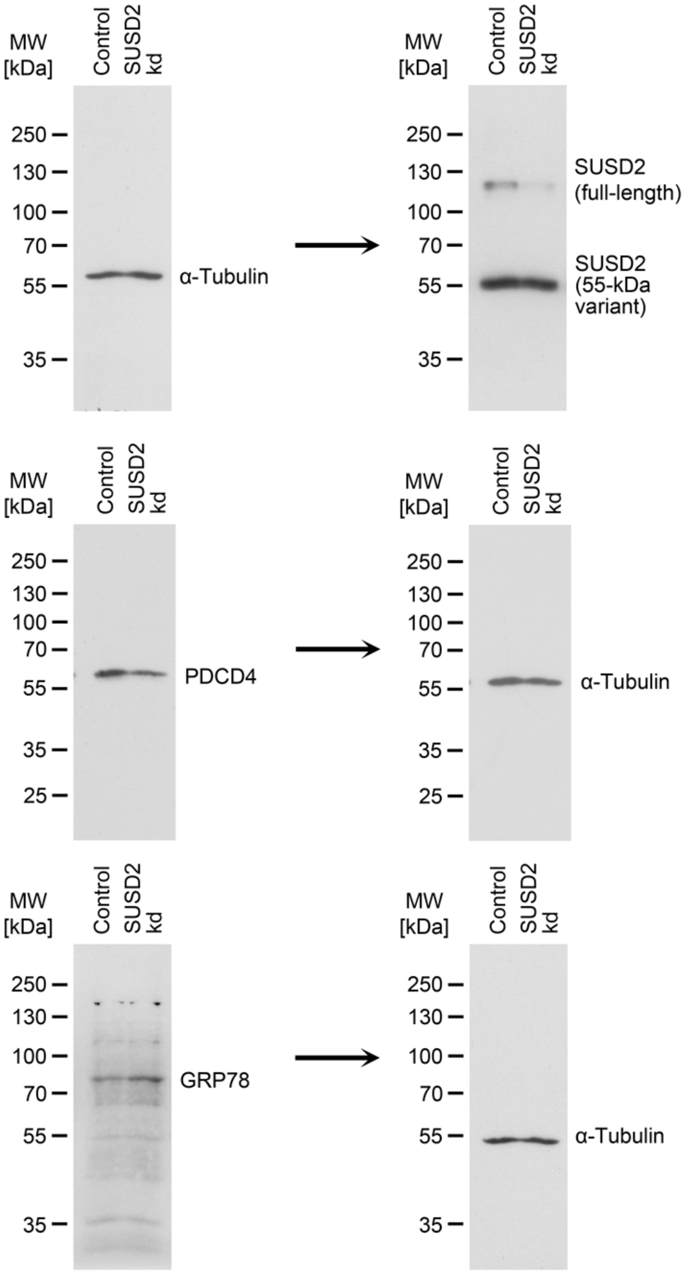

For Figure 5C:

CTC-ITB-01:

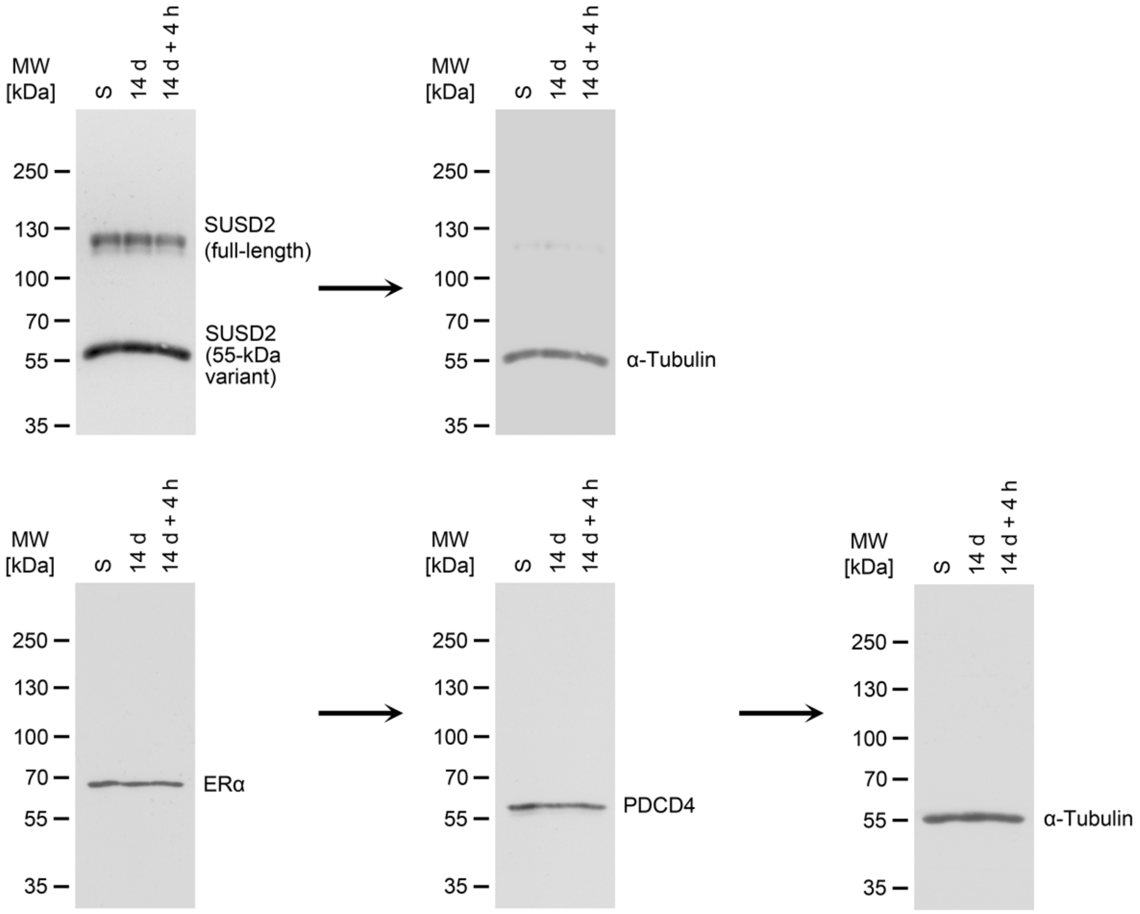

MCF-7:

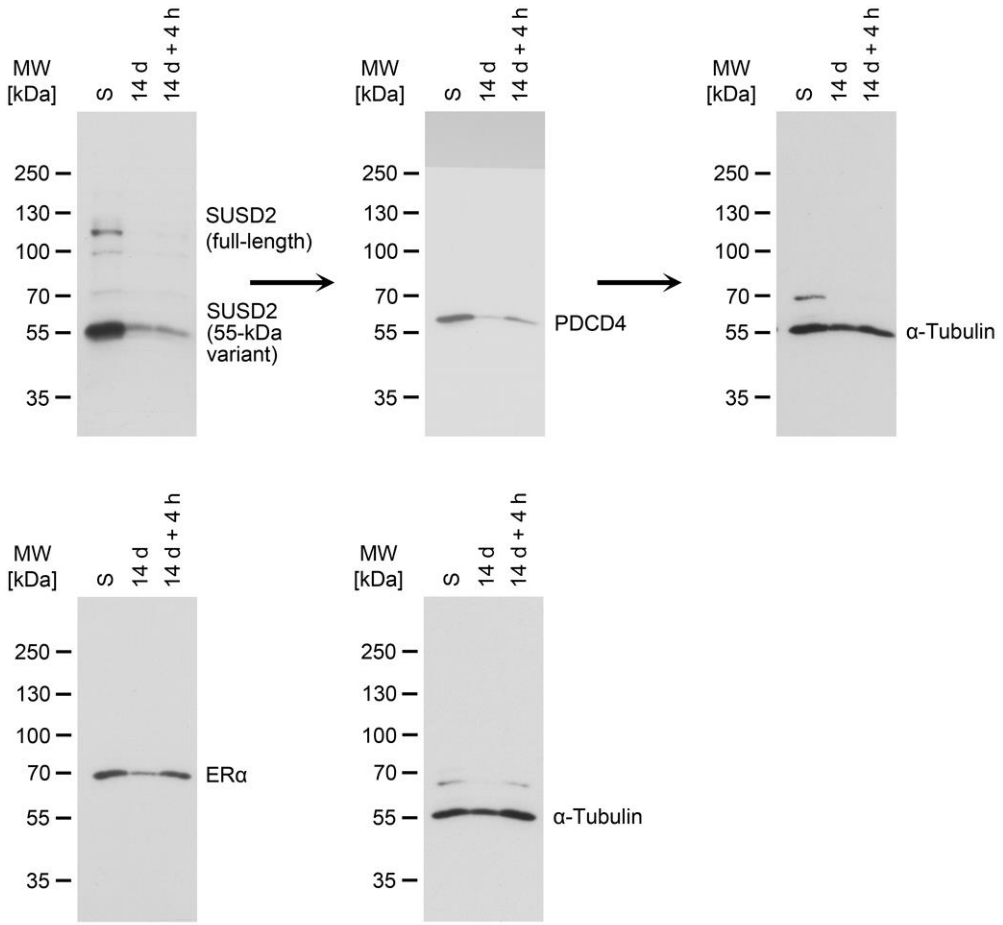

MDA-MB-468:

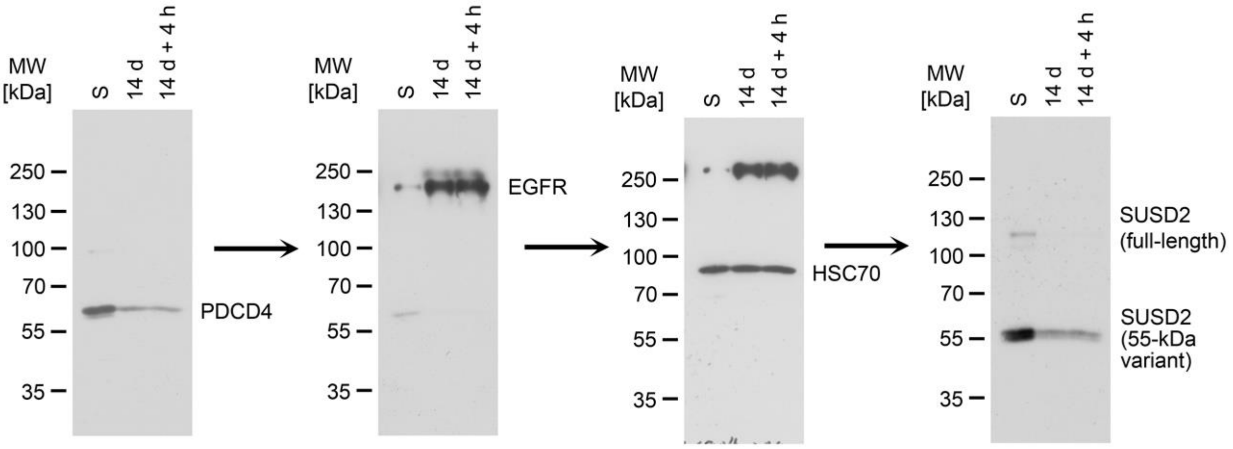

For Figure S1:

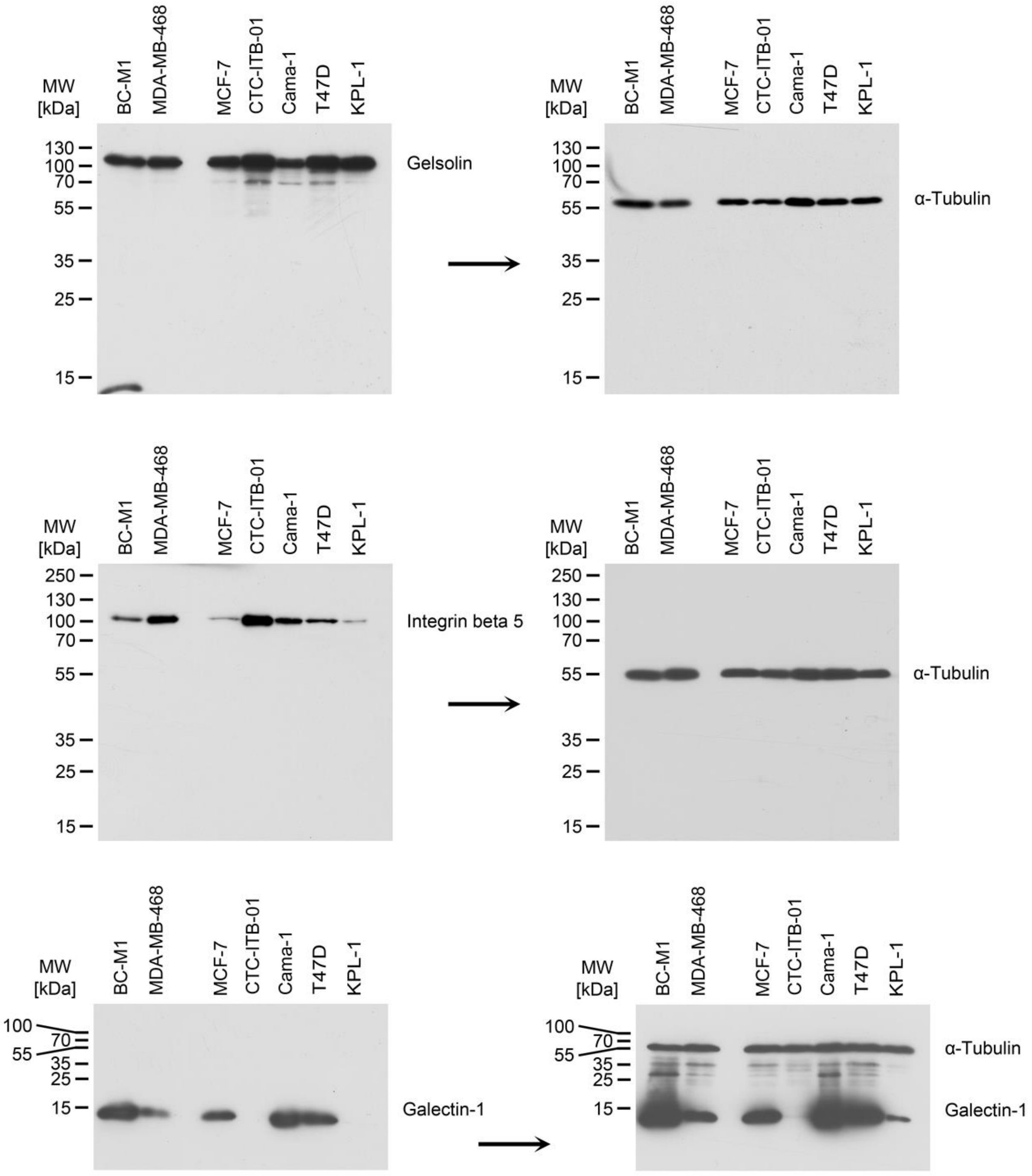

For Figure S6:

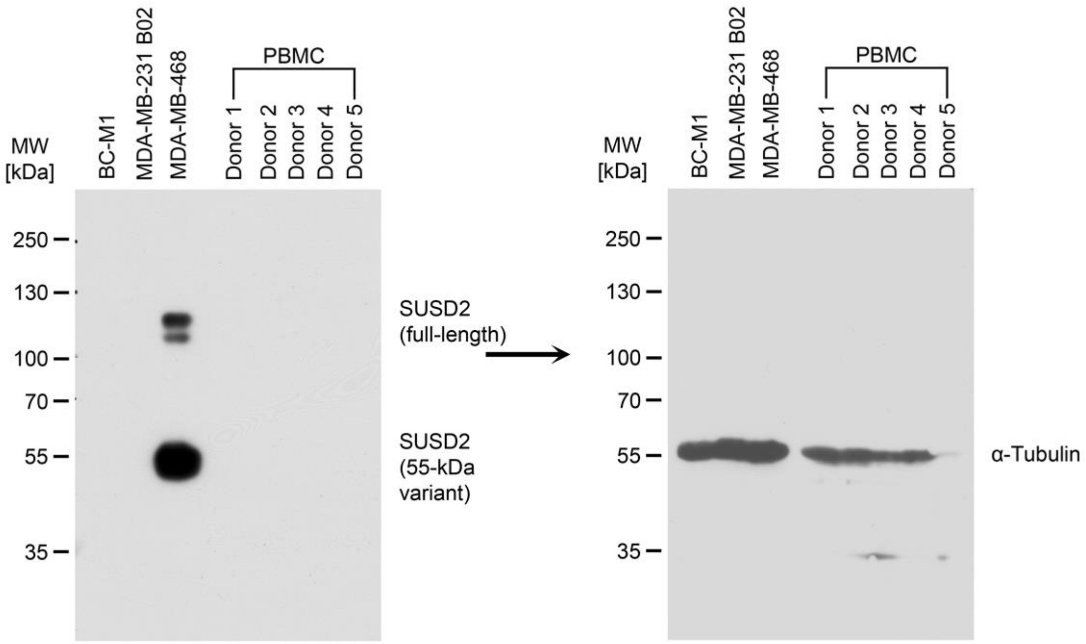

Supplement: Supplementary file 3 — Supplementary Material 3 [file 41598_2025_87122_MOESM3_ESM.pdf]
